# Revision of the *Megasoma* (*Megasoma*) *gyas* (Jablonsky in Herbst, 1785) species group (Coleoptera, Scarabaeidae, Dynastinae)

**DOI:** 10.3897/zookeys.999.53130

**Published:** 2020-11-30

**Authors:** Massimo Prandi, Paschoal C. Grossi, Fernando Z. Vaz-De-Mello

**Affiliations:** 1 Via Del Seminario 16, I-25087, Salò, BS, Italy Unaffiliated Salò Italy; 2 Universidade Federal Rural de Pernambuco, Departamento de Agronomia/Fitossanidade, Laboratório de Taxonomia de Insetos, Programa de Pós-graduação em Entomologia Agrícola-PPGEA, 52171-900, Recife, Pernambuco, Brazil Universidade Federal Rural de Pernambuco Recife Brazil; 3 Universidade Federal de Mato Grosso, Instituto de Biociências, Departamento de Biologia e Zoologia, Coleção Entomologica, 78060-900, Cuiabá, Mato Grosso, Brazil Universidade Federal de Mato Grosso Cuiabà Brazil

**Keywords:** Cerrado, Neotropical region, new species, Scarabaeoidea, South America

## Abstract

The taxa of the genus *Megasoma* Kirby, 1825 (Coleoptera, Scarabaeidae, Dynastinae) related to *M. gyas* (Jablonsky in Herbst, 1785) are revised. Megasoma (M.) gyas is recognized as a monotypic species restricted to the Caatinga biome of northeastern Brazil. *Megasoma gyas rumbucheri* Fischer, 1968, is considered as a new synonym of *M. gyas*. The “long-horned *M. gyas*” is recognized as a separate polytypic species M. (M.) typhon (Olivier, 1789) with the nominative subspecies occurring through the Mata Atlântica biome of Brazil, from Bahia to São Paulo states and M. (M.) typhon prandii Milani, 2008 restricted to a small area in the state of Santa Catarina, South Brazil. *Megasoma gyas porioni* Nagai is considered as a new synonym of *M. typhon typhon*. The “short-horned *M. gyas*” occurring in Minas Gerais, São Paulo, and southwestern Bahia, is recognized as a separate new species and described as M. (M.) hyperion**sp. nov.** The paper includes an historical research and the redescriptions of the other nominal species of the genus. Distribution maps and a key to species in the M. (M.) gyas species group (males and females) are also provided.

## Introduction

Megasoma (Megasoma) gyas (Coleoptera, Scarabaeidae, Dynastinae), locally known as “besouro de chifre” or “besouro com chifre” or “grande besouro”, is perhaps the most interesting species among all the large-sized South American *Megasoma*. Unlike its glabrous related species, i.e., the species of the *M. actaeon* (Linnaeus, 1758) group, it displays a thick cover of short shiny setae on the whole dorsum, a feature shared with *M. anubis* (Chevrolat in Guérin, 1836) and *M. joergenseni* Bruch, 1910. Through the examination of the type material as well as a large series of specimens from several localities, it became possible to re-define the species and to isolate three taxa that deserve a separate species or subspecies status. In this paper a new species distributed in open Cerrado areas, as in some transitional areas of Cerrado and Caatinga, ranging from São Paulo to Bahia states of Brazil, is described. Furthermore, we propose the new synonymy of M. (M.) gyas over *M. rumbucheri*, the use of M. (M.) typhon over *M. gyas* for the current “long-horned *M. gyas*”, and the new synonymy with *M. typhon* for the previous synonyms of *M. gyas*, *Scarabaeus entellus* Olivier, 1792, and *S. monoceros* Weber, 1801.

## Materials and methods

In total, 328 specimens were studied (all wild-collected). The specimens were examined through naked eye observation, or/and with a stereomicroscope. Pictures were taken with a digital camera Canon Powershot S50 and a Leica M5 stereomicroscope and focus stacked with the Combine ZP software. Dissection of male genitalia was made by extraction with forceps through an aperture between tergite VI and the propygidium. The parameres were then glued on a card and pinned below the specimens. The distribution maps were made using a base map suitable for the purpose and available on the Internet. Each specimen of the type series of the new species bears a red label: “*Megasoma hyperion* sp. nov. / [Holotypus] Paratypus / M. Prandi, P.C. Grossi & F.Z. Vaz-de-Mello det. 2020 [numbered from 1 to 154]”.

**List of abbreviations**

**CL** cephalic horn length measured along the external curve

**EL** elytral maximum length

**EW** elytral maximum width

**FL** fore tibia length

**HL** head length

**L** body length from the clypeal apex to the elytral apex

**PH** lateral pronotal horn length from base

**PL** pronotum maximum length

**PW** pronotum maximum width

**TF** fore tarsus length

**TH** lateral thoracic horn length from base

**TL** length from the tip of cephalic horn to the elytral apex

**Depositories of examined material**

**BMNH**The Natural History Museum, London, UK (Maxwell Barclay);

**CEMT** Universidade Federal do Mato Grosso, Instituto de Biociências, Cuiabá, Brazil (Fernando Z. Vaz-de-Mello);

**CERPE**Universidade Federal Rural de Pernambuco, Recife, Brazil (Paschoal C. Grossi);

**DZUP**Universidade Federal de Paraná, Centro Politécnico, Curitiba, Brazil (Lucia Massutti de Almeida);

**EPGC** Everardo and Paschoal C. Grossi collection, Nova Friburgo, Rio de Janeiro, Brazil;

**EUMJ**Ehime University, Entomological Department, Matsuyama, Japan (Hiroyuki Yoshitomi);

**INPA**Instituto Nacional de Pesquisa da Amazônia, Manaus, Brazil (Márcio L. de Oliveira);

**KKC** Kazuho Kobayashi private collection, Tokyo, Japan;

**LMC** Leonello Milani private collection, Calvignasco, Milano, Italy;

**MPC** Massimo Prandi private collection, Salò, Brescia, Italy;

**MPEG**Museu Paraense Emilio Goeldi, Belém, Brazil (Orlando Tobias);

**MSNM**Museo Civico di Storia Naturale, Milano, Italia (Fabrizio Rigato);

**MZC** Michele Zilioli private collection, Buguggiate, Varese, Italy;

**UBC** Ugo Bosia private collection, Asti, Italy;

**UNLP** Facultad de Ciencias Naturales y Museo de la Universidad Nacional de La Plata, La Plata, Argentina (Analia Lanteri).

## Nomenclatural and taxonomic history of the taxon *Megasoma gyas*

### Pre-Linnaean accounts

**Figures 1, 2.**
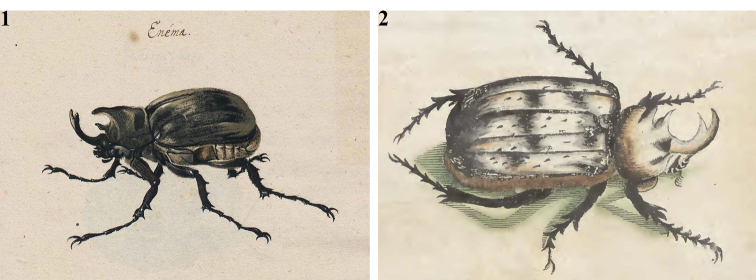
**1** George Marcgraf, “Libri Principis” of Libri Picturati, before 1648, page 477, “*Enéma*” (indigenous name) **2** W. Piso & G. Marcgraf, “Historia Naturalis Brasiliae” 1648, page 246. *Enéma*, *Enena*, *Escaravelha Lusitanis*, *Taurus Volans*.

In the year 1637, the Count Johan Maurits van Nassau-Siegen became the General Governor of the Dutch North and Northeast Brazil under the Geoctoyeerde Westindische Compagnie, (the Dutch West-Indies Company). He invited, among other scientists and artists, George Marcgraf (or Marcgrave) to Brazil for the first scientific expedition of the area, which took place between 1637 and 1644. During the expedition beautiful oil and watercolor plates, showing maps and scientific subjects, such as people, plants, animals, and insects, were executed by Albert Eckout, Frans Post, Zacharias Wagner, and by George Marcgraf himself (Boeseman et al. 1990; Sick 1997; Dantas Silva 2005; Corrêa do Lago 2010; Teixeira Leite 2014; Ossenbach 2017; Anderson 2019). A big part of those plates was rejoined into the “Libri picturati” now housed in Krakow, Poland. Among the subjects of the “Handbook” (“Libri principis”) of “Libri picturati” there is a beautiful plate showing a *Megasoma gyas* (Fig. 1). It was the first time that this species was brought to the attention of the Western world.

Subsequently, another illustration, a poorly executed drawing of the same beetle, was published in the “Historia Naturalis Brasiliae” of 1648 by Piso and Marcgraf (Fig. 2). This plate was the one seen by Linnaeus (1758) and listed by him among the references he provided with a description of *Scarabaeus actaeon* (see Prandi 2018a, 2018b; 2019). Despite the poor quality of the drawing, there is little doubt that it represents a *Megasoma gyas* specimen, an interpretation supported by the accompanying text: “the body is covered by yellowish pilosity…the first section of the body is three-horned…”. The chapter of the book dealing with *Megasoma gyas* is entitled “Enema rare conformationis” and the *Megasoma* beetle was called “Taurus volans” (flying bull) together with the other three beetles. Marcgraf’s first xylography at page 246 (Fig. 2), and the related gouache color image in “Libri picturati” (Fig. 1) show clearly a short-horned *Megasoma*: this is the most relevant character referring to *Megasoma gyas* s. str.

George Marcgraf (1610–1644) was a German naturalist and astronomer who, at the beginning of the Dutch expedition, was an attendant of the famous physician Wilhem Piso. Day after day, thanks to his enthusiastic work, he gained the favor of the Dutch Governor. Eventually, there were four books written by Piso on the traditional Brazilian medicine, and eight books on the natural sciences written by Marcgraf, all gathered in the “Historia Naturalis Brasiliae” (1648), funded by J. Maurits van Nassau after his return to Holland and four years after Marcgraf’s death.

“Historia Naturalis Brasiliae”, until the beginning of XIX century, represented a source of important information on Brazilian natural history for European scientists, including Linnaeus, who referred to it in the 10^th^ and 12^th^ editions of his “Systema Naturae”, often using Marcgraf’s or Piso’s descriptions, all validated for scientific purposes with binomial Latin names, as the only basis for establishing his species (Boeseman 1994). Marcgraf’s drawings and descriptions of course referred to the places that Marcgraf visited himself, i.e., the old Dutch “Captaincy” of the northeast, which included the current states of Paraíba, Pernambuco, Alagoas, Ceará, Piauí, and Rio Grande do Norte. In fact, during his seven-year stay, Marcgraf undertook various expeditions to visit the interior of northeastern Sertão to develop his detailed work on the natural history of Brazil (Alcantara-Rodriguez et al. 2019). At least three expeditions of forty, twenty, and eleven days’ duration between 1638 and 1640 were undertaken, most likely within the Pernambuco, Paraíba and the Rio Grande do Norte regions. These field expeditions allowed him and Piso to collect and catalog animals and plants from different environments, from the lagoons of the coast to the interior of the Caatinga (Teixeira 1995). Hence it is very likely that the information given by Marcgraf on *Megasoma gyas* refers to the specimens observed or collected in those regions and then given to Count J. Maurits. Indeed, Marcgraf confided to Nassau also dried plant specimens and the manuscripts about Brazilian natural history before leaving for Angola between 1643 and 1644 (Whitehead 1979).

### Post-Linnaean scientific reports

Jablonsky (1785) described *Scarabaeus gyas* following the binominal nomenclature and wrote: “...in the lower part of the head there is a horn, considerably wide but hidden, in the shape of a shovel, which the more it lengthens and the more it becomes wider, towards the end it ends in two long teeth…the armor is completely covered by yellow hair…in addition to this sickle-shaped horn the armor stretches downwards and both sides form a point…so it can also be called tricorn thorax…the elytra are thick and covered with yellow hair, which give to the insect an unusual sumptuous appearance…”. The description is enriched by a precious color plate by Jablonsky himself.

Carl Gustav Jablonsky (1756–1787) was a German scientist and illustrator, private secretary of the Queen of Prussia. Although “Natursystem alles bekannt....” is attributed to JFW Herbst (1743–1807), it must be noticed that Jablonsky was the author of the first volumes of that work (Bousquet 2016), i.e., the first volume on butterflies and the first volume on beetles. Herbst took over the job after the untimely death of Jablonsky in 1787, at the age of 31. It is important to note that also in Jablonsky’s plate the specimen of *Megasoma gyas* displays a short and wide cephalic horn. This particular shape of the cephalic horn matches exactly both the painted picture and the xylography in Marcgraf’s publication. This is a key point because in 1789 the French scientist Guillaume-Antoine Olivier (1756–1814) in his “Entomologie ou histoire naturelle des insectes” described *Scarabaeus typhon* and *Scarabaeus laniger*. The specimens of Olivier show two different features of the cephalic horn: long, thick, with a bifurcated apex the former, as shown in his plate XVI fig. 252 (reproduced in Fig. 5) and shorter, flatter and wider, with a very-well bifurcated apex the latter, as in his plate XXVIII fig. 247 (reproduced in Fig. 6). Olivier’s *Scarabaeus typhon* actually represents the species that up to now has been treated as *Megasoma gyas* s.l. by the majority of authors. Burmeister (1847) established the synonymy between *Megalosoma typhon* Olivier (*Megalosoma* Burmeister is a junior synonym of *Megasoma* Kirby) and *Scarabaeus gygas* Jablonsky, giving priority to the former. Later, Harold (1868) maintained the synonymy by Burmeister, but in the addendum (Harold 1871: 121–122) he suggested the priority of *M. gyas* over *M. typhon*. *Scarabaeus laniger* Olivier, 1789 was correctly synonymized by Burmeister (1846: 106–108) with *M. gyas*, with priority given to the latter. Burmeister (1847: 277–278) incorrectly treated *S. laniger* as a synonym of *M. typhon*, considered as “variation B”, a variation with a short horn similar to that of *S. gyas* and *S. esau*. Before him, Kirby and Spence (1826) cited *S. typhon* and *S. lanigerum* as separate species. But, taking into account Harold’s subsequent actions, now *S. laniger* is correctly considered a synonym of *Megasoma gyas*, with which it shares the same specific characters. As for the types of *S. typhon* and *S. laniger*, they were illustrated in the above-mentioned plates by Olivier (1789). To prepare his book, Olivier traveled through England and Holland to visit the private cabinets of collectors and to draw the species which were not available in Paris. Likely, the specimens of *S. typhon* and *S. laniger* illustrated by Olivier were kept in the collections he visited during the aforementioned trips. Olivier’s reference under the description of *S. laniger*, “du cabinet de Mr. Juliaans” (from the collection of Mr. Juliaans), clearly indicates a Dutch family name. As for *S. typhon*, Olivier reported no localities, apart from the indication “du Musée Britannique” (from the British Museum). A recent search by Kazuho Kobayashi at the Natural History Museum in London revealed some old specimens (from the Fry collection, dated approximately 1900) of classical “long-horned *M. gyas*” coming from Rio de Janeiro and Bahia, but not the specimens seen by Olivier.

Jablonsky (1785) cited three references. The first reference is “Fuessly Mag. I. p. 37”: Johan Caspar Fuessly (1778) referred to Voets (1766) and copied in his work (Hagen 1857). The Latin description of the beetle is the same as reported by Jablonsky; there is also a reference to illustration, “Tab. XVII fig. 114”. The second reference is “*ScarabaeusGoliath*. Voet. Scar, tab. 17. fig. 114”. The third reference is “Goeze Ent, Beytr. I. p. 56. n.11”: Johan August Ephraim Goeze (1777) also referred to Voets (1766), giving a short Latin description and the title “Goliath, der amerikanische gelbe Bar”. It is clearly the same insect.

**Figures 3, 4.**
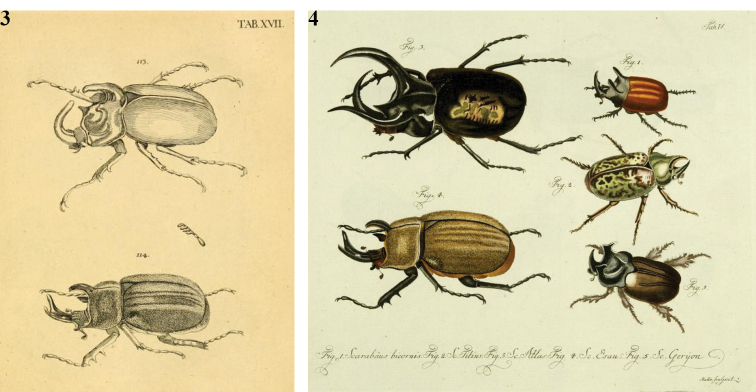
**3** Johann Euseb Voets, 1782 edition of “Kaferwerk”, fig. 114. *ScarabaeusGoliath*. **4** Carl Gustav Jablonsky 1785. “Natursystem aller bekannten…” Vol. I. Kafer, fig. 4. *Scarabaeus gyas*.

Johann Euseb Voets (1706–1778) died before the publication of Jablonsky, hence it is obvious that his description of *ScarabaeusGoliath* must be precedent, also because Fuessly (1778) and Goeze (1777) referred to him. Voets’ main work, the “Catalogue raisonné ou systématique du genre des insectes, qu’on appelle Coleoptrées”, was apparently published in parts starting from 1766. The first part was mentioned, without details, in April 1767 by the “Gazette littéraire de l’Europe”. Other parts were issued in 1776 and 1781. Finally, the work was completed and published by Bakhuysen in 1806, under the name “Catalogus systematicus Coleopterorum” (Bousquet 2016). In the work “Beschreibungen and Abbildungen…” (1785), published after his death and illustrated by GWF Panzer, the above-cited fig. 114 in plate XVII (reproduced in Fig. 3) perfectly matches Jablonsky’s image. In fact, Jablonsky wrote that he tried to find a good description of the insect drawn by Voets from the collection of Dr. Luchmann, but without result. Hence, he decided to copy the drawing, giving a better description and changing the name “*Goliath*”, already used by Linnaeus for another beetle without horns, with the name of a Titan, *Gyas*.

Therefore, Voets’ name *ScarabaeusGoliath* should have priority over *M. gyas*. However, Voets’ “Catalogus systematicus Coleopterorum” fails to fulfill the requirements of Article 11.4 of the ICZN, namely that for scientific names to be available the entire work in which they appear must be consistently binomial. Voets’ names varied from two to five names in series, thus violating this rule, so none of Voets’ names, even those which happened to be binomial, are available in the sense of the ICZN. The original Luchmann’s specimen is apparently lost. The iconography by Marcgraf and Voets/Jablonsky allows us to state that the first “*gyas*” described following the binominal nomenclature had a short, flat, and wide cephalic horn (Figs 3, 4). In this case, since Jablonsky’s type does not match Olivier’s *S. typhon*, necessary changes in nomenclature need to be made. In Fig. 4 (a reproduction of one of Jablonsky’s plates), the insect is named *Scarabaeus esau*. For this reason, *S. esau* is considered as a synonym of *M. gyas*. Some plates appeared with the name *S. gyas*, some other with the name *S. esau*. But no description of *S. esau* was provided.

**Figures 5–7.**
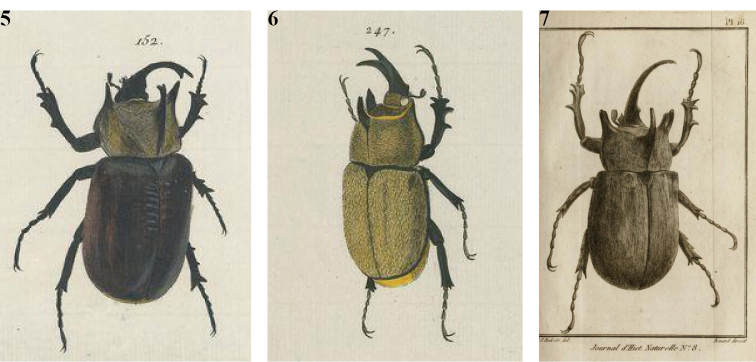
**5** G-A Olivier 1789. “Entomologie ou Histoire Naturelle des Insectes”, Tav. XVI fig. 252. *Scarabaeus typhon***6** G-A Olivier 1789. “Entomologie ou Histoire Naturelle des Insectes”, Tav. XXVIII fig 247. *Scarabaeus laniger***7** G-A Olivier 1792. “Journal d’Histoire Naturelle”, no. 8. *Scarabaeus entellus*

**Figure 8.**
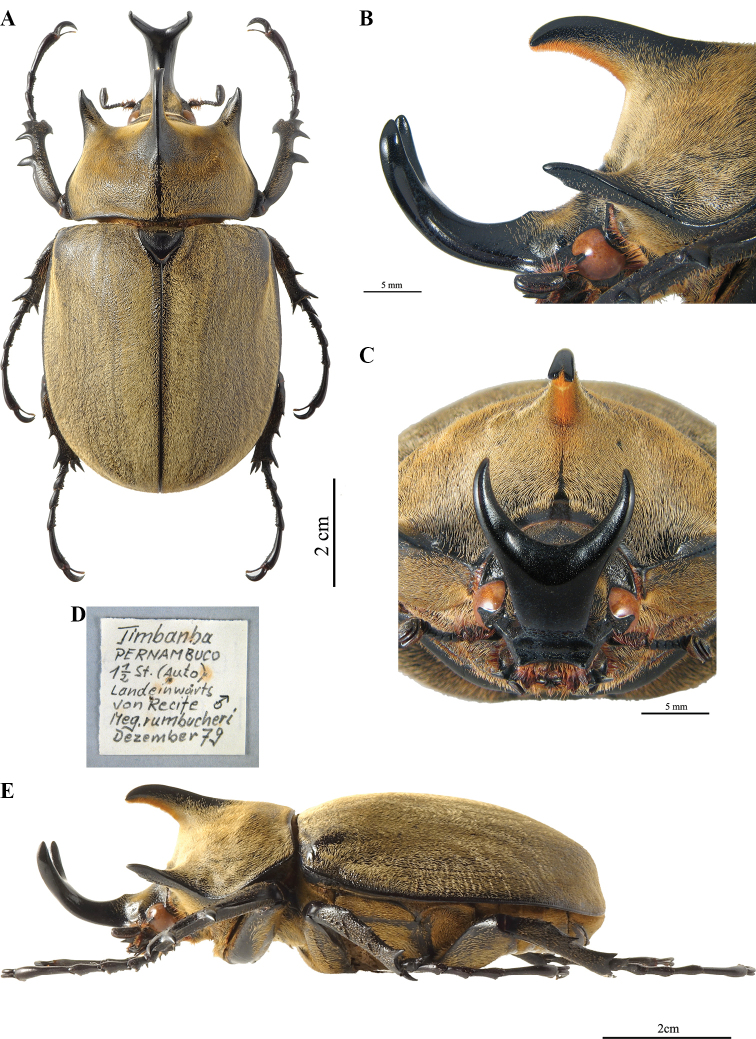
*Megasoma gyas* ♂ from Brazil, Pernambuco **A** dorsal habitus **B** cephalic horn lateral view **C** cephalic horn frontal view **D** original label **E** lateral habitus.

Fischer (1968) described *M. rumbucheri* (afterwards considered a subspecies of *M. gyas*: see Endrödi 1977) from Rio Pajeú, Planalto da Borborema, Pernambuco, Brazil. But this taxon actually displays the same characters of Marcgraf and Voets/Jablonsky original descriptions and is therefore a junior synonym of *M. gyas* (Fig. 8). Curiously, in 1991 Kurt Rumbucher, to whom the Fischer’s subspecies had been dedicated, suggested that *M. rumbucheri* fell within the variability of *M. gyas* s.l. This opinion was supported by some photos of *M. gyas* s.l. specimens of different sizes and tables with measurements. He did not examine the aedeagus nor assessed the geographical variability of the specimens he had studied.

Nagai (2003) described *M. gyas porioni* from Jaguaquara, Bahia state, Brazil, dedicated to the French entomologist Thierry Porion. The main character currently in use to distinguishing this subspecies is a long, normally straight, cephalic horn (although in the original description it is indicated as short and thicker in the middle area), showing in the majority of cases a medial depressed zone, and a normally bifurcate apex (Fig. 15). This character is found in the type of *S. typhon* Olivier. It can be concluded that no reasonable external differences between the “long-horned *M. gyas*”, *M. gyas porioni*, and Olivier’s *S. typhon* can be found (Figs 5, 7). Milani (2008: 120) reported the inconsistency of characters between the original description and the specimens from the type locality. The synonymy between *M. gyas porioni* and the “long-horned” *M. gyas* was also suggested by Grossi et al. (2008).

Grossi et al. (2008: 364–365) hypothesized the presence of *M. gyas* in the state of Santa Catarina and the same year Milani described *M. gyas prandii*, from Santa Catarina state, Brazil. This is the southernmost distribution record of *M. gyas*. Unlike the aforementioned subspecies, in this case, both the geographical isolation and the peculiar morphology of the taxon leave little doubt about its validity as a subspecies, although, due to the present new arrangement, it must be considered as a subspecies of *Megasoma typhon*. None of the specimens of *M. typhon prandii* we have examined thus far show a depressed area in the middle of the cephalic horn. All examined specimens show a thick long horn, often curved backwards, without any flattened area, with a distinctly bifurcate apex bent upwards. Besides the type locality, it was recently possible to find (authors’ unpublished data) other specimens of *M. typhon prandii* in old collections, from even more southern localities, labeled “Porto Alegre” (Rio Grande do Sul, Brazil) or “surroundings of Porto Alegre” (ca. 1930-40, collection of Ugo Bosia, Asti, Italy). Interestingly, one of those old specimens bears the label “*M. typhon*”.

## Taxonomic treatment

### Megasoma (Megasoma) gyas species group

The *Megasoma gyas* species-group consists of three species, including one polytypic, with an overall distribution range occupying most of eastern Brazil, extending northwards up to Ceará (estimated latitude 3°42'02"N) and southwards to Rio Grande Do Sul (estimated latitude 30°00'44"S) states.

#### Megasoma (Megasoma) gyas

Taxon classificationAnimaliaColeopteraDynastidae

(Jablonsky in Herbst, 1785)

65D234ED-642E-554F-846F-4A756C976EDF

Figures 8A–E , 13A–C

Scarabaeus gyas Jablonsky, 1785: 263–267

Scarabaeus Goliath Voets, 1766 (unavailable name).
Scarabaeus esau Jablonsky, 1785; synonym by Burmeister 1847: 277–278.
Scarabaeus laniger Olivier, 1789; synonym by Burmeister 1846: 106–108.
Megasoma gyas rumbucheri Fischer, 1968, syn. nov.

##### Type material.

The holotype is the specimen seen and illustrated by Voets, whose illustration was later copied by Jablonsky (1785: Plate 4 fig. 4) and is probably lost.

##### Distribution.

**Figures 9, 10.**
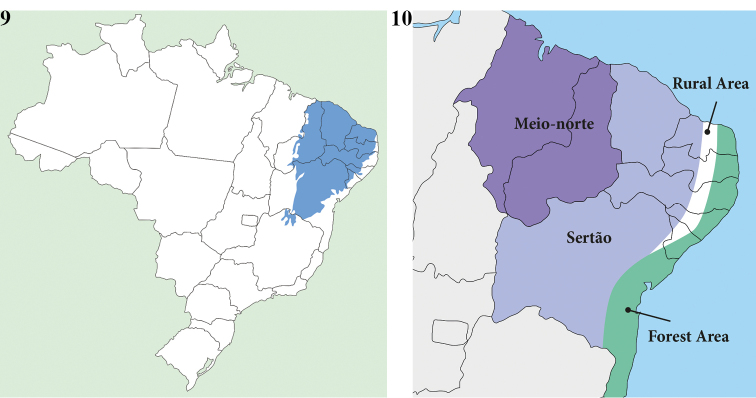
**9** Surface of bioma Caatinga in Brazil **10** The subregions in NE Brazil.

*Megasoma gyas* occurs in the Caatinga biome of the Brazilian states of Piauí, Ceará, Rio Grande do Norte, Paraíba, Pernambuco, Alagoas, and Sergipe (Fig. 9). The distribution range of this species overlaps a portion of the “subregioes nordestinas” (Vasconcellos et al. 2010; Beserra Nobre et al. 2014) of “Meio-norte” and “Sertao” regions (Fig. 10). The Caatinga biome (xeric shrubland and thorn forest) occupies an area of 497,000 sq. miles, i.e., 10% of Brazilian territory (Fig. 9); it is a recent biome located on an ancient seabed. This biome experiences long periods of drought, which can last up to eight months. It is mainly composed of dry Savannah (Coutinho 2016).

##### Material examined.

13 major ♂, 4 minor ♂, and 7 ♀ from the following Brazilian states: **Alagoas**, Canapi, X 2018, J. Dantas Leg. (1 ♂, CERPE, 1 ♀, MPC); **Pernambuco**, Timbaúba, with indication of 1 hour and half by car inland of Recife, XII 1979 (1 ♂ and 1 ♀, MPC); Areia, VI 1945 (1 ♂, MPC); Araripina, 1953 (2 ♂, LMC); Brazil, Estaçao …(not visible), G.L.Sladen Leg., 1925 (2 ♂, 1 ♀, BMNH); Custódia, X 2012, leg. C.E.B Nobre (1 ♂, MPC); São José do Egito (sitio Humaitá), IV 2010 Leg. R.M. Correia (1 ♂, CEMT); Igarassu, Três Ladeiras, Usina São José XII 2017, (1 ♀, CERPE); **Sergipe**, didactic collection of the University, no further data (1 ♂, CERPE, 1 ♂, MPC); Capela, Ref. Vida Silv. – Mata do Jumco Station (RUSMJ), III 2014 Leg. O.G. Moura (1 ♂, CERPE); **Ceará**, no data (1 ♂, CERPE); Ceará, no data (1 ♂, 1 ♀, EPGC); **Piauí**, didactic collection of the University, Teresina, São Francisco, IV 2001 (1 ♂, CERPE); **Rio Grande do Norte**, Tenente Laurentino Cruz, I 2015, leg. R. Andreazze (1 ♂, CERPE, 1 ♀, MPC); **Paraiba**, Patos, no further data, (1 ♂, CERPE, 1 ♀, MPC).

**Male (Fig. 8). *Size*.** L: 77 mm; TL: 88 mm; PL: 21 mm; PW: 35 mm; EL: 49 mm; EW: 42 mm; CL: 23 mm; PH: 8.5 mm. ***General appearance*.** Uniformly dark brown, covered by yellowish short, fine, dense pubescence; head, including horn, consistently black with yellowish sparse bristles at base. ***Head*.** Cephalic horn: short, projecting forwards and curved upwards. In lateral view flat, distally bent upwards. In dorsal view, narrower at base gradually broadened towards distinctly forked apex. Apex U-shaped, with slightly divergent, long, tips (Fig. 8C). Distance between tips 11.5 mm. Sides bordered with a weak, hardly detectable rim, from base to mid-length. Dorsal side at base with relief of an almost imperceptible tooth. ***Pronotum*.** Whole surface covered by fine, dense yellowish pubescence. Anterior angles projecting as small but elongate, sharp, parallel horns, slightly bent outwards; width of horn at base 4.5 mm; length from base to tip 8.5 mm; distance between apices of anterior horns 23 mm. Median thoracic horn longer than lateral ones, length 15 mm, with characteristic sickle-shaped form, dorsal side with glossy black line, ventral side of median horn with recumbent fine pubescence. PL/TH ratio 2.470. L/PL ratio 3.666, showing a fairly elongated feature of the body. ***Elytra*.** Surface covered by fine, dense, recumbent, yellowish pubescence apart from elytral suture and epipleura, glabrous; EL/EW ratio 1.166. Elytral surface covered by (two or three on each elytron) visible longitudinal ridges: sutural edge black, glabrous, punctate; other pubescent ridges spaced out. Elytra in lateral view more convex proximally and then gradually flattened towards apex. L/EL ratio 1.571, elongate.

***Abdomen*.** Sides covered with short, very fine, yellowish brown pubescence, medially almost glabrous. ***Legs*.** Fore tibia almost straight, inner apical edge strongly dilated inwards, 23 mm in length. Anterior edge of protibia V-shaped. Lateral edge with three strong teeth, decreasing in size proximally, from base to apex; basal tooth more distant from subapical tooth than the latter from the apical tooth. Basal and subapical teeth large, triangular, thick, sharp, pointing rearwards; apical tooth short, pointing forwards. Inner apical spur strongly curved downwards, as long as apical tooth. Fore tarsi length 25 mm. ***Aedeagus*.** Parameres of tegmen elongate and narrow, as in Fig. 31A, B. Thickness of section from anterior phallobase to median lobe also narrow.

##### Variation, males.

Major and medium males always with apex of cephalic horn U-shaped, with long tips. Minor males with cephalic horn length not more than twice head length, from vertex to clypeus, bear the apex of cephalic horn V-shaped with shorter tips and wider body. Dorsal tooth of cephalic horn absent in medium and minor males.

##### Measurements.

L: 53–77 mm; TL: 59–88 mm; PL: 14–21 mm; PW: 24–35 mm; EL: 38–49 mm; EW: 30–43 mm; CL: 10–23 mm; FL: 15–23 mm; TF: 17–25 mm.

**Female (Fig. 13). *Size*.** L: 54 mm; PL: 16 mm; PW: 23 mm; EL: 34 mm; EW: 30 mm. ***General appearance*.** Uniformly black; elytra with 6/7 of its surface covered by grey-brownish dense pilosity. ***Head*.** Fronto-clypeal suture with a double conical tubercle. ***Pronotum*.** Surface dull, coarsely punctate-rugose, strongly convex; posterior median carina 8 mm long, ½ of total PL. Anterior angles obtusely projecting, yet with sharp tips. Lateral edges with the presence of sparse bristles. ***Elytra*.** Surface with puncture mixed to wrinkles anteriorly, glossy black; punctate black surface extending for 8 mm. Elytral pubescence thick, uniform, with clearly visible longitudinal ridges, three or four for each elytron, almost equidistantly spaced out. Dorsal suture and lateral margins glossy black, with very fine punctation. ***Abdomen*.** Sternites finely punctate, covered by short, yellowish brown pilosity, except for a small central portion in the middle of sternites III–V. ***Legs*.** Fore tibiae shorter than in males, TL 15 mm, and shorter than tarsi, TF 17 mm; external sides with three strong teeth almost equal in length, with the subapical tooth a little longer. Lateral teeth and inner apical spur smaller than in males.

**Figure 11.**
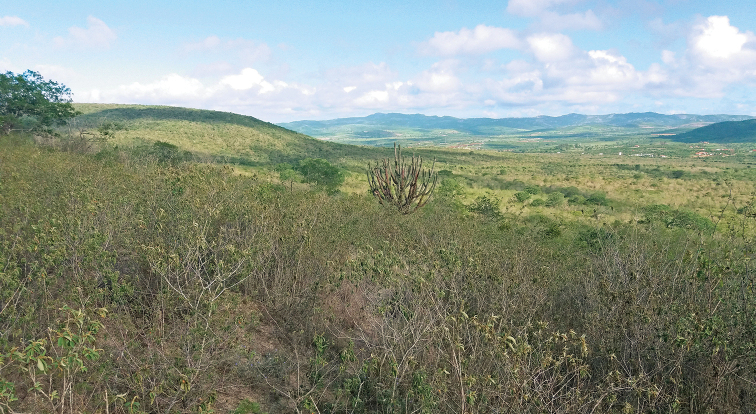
View of Caatinga biome near Sairé, Pernambuco (photograph P. Grossi).

##### Measurements.

L: 52–60 mm; PL: 15–16 mm; PW: 23–27 mm; EL: 34–39 mm; EW: 30–34 mm; FL: 15–16 mm; TF: 17–18 mm; HL: 6–8 mm.

**Figure 12.**
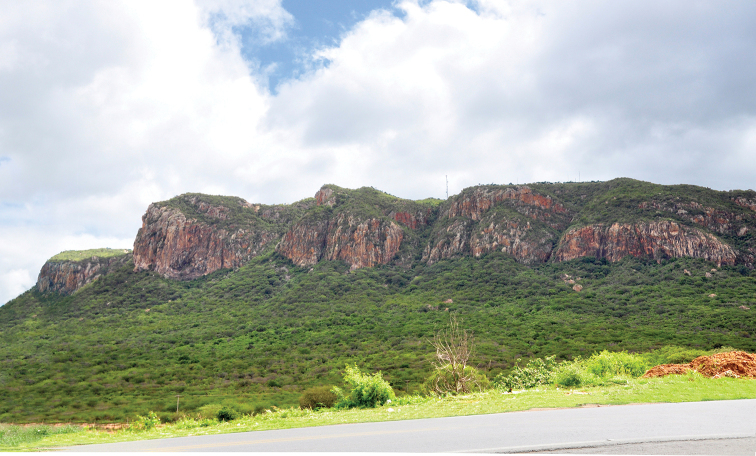
View of Serra Talhada, vale do Rio Pajeú, Pernambuco. (photograph P. Grossi).

**Figure 13.**
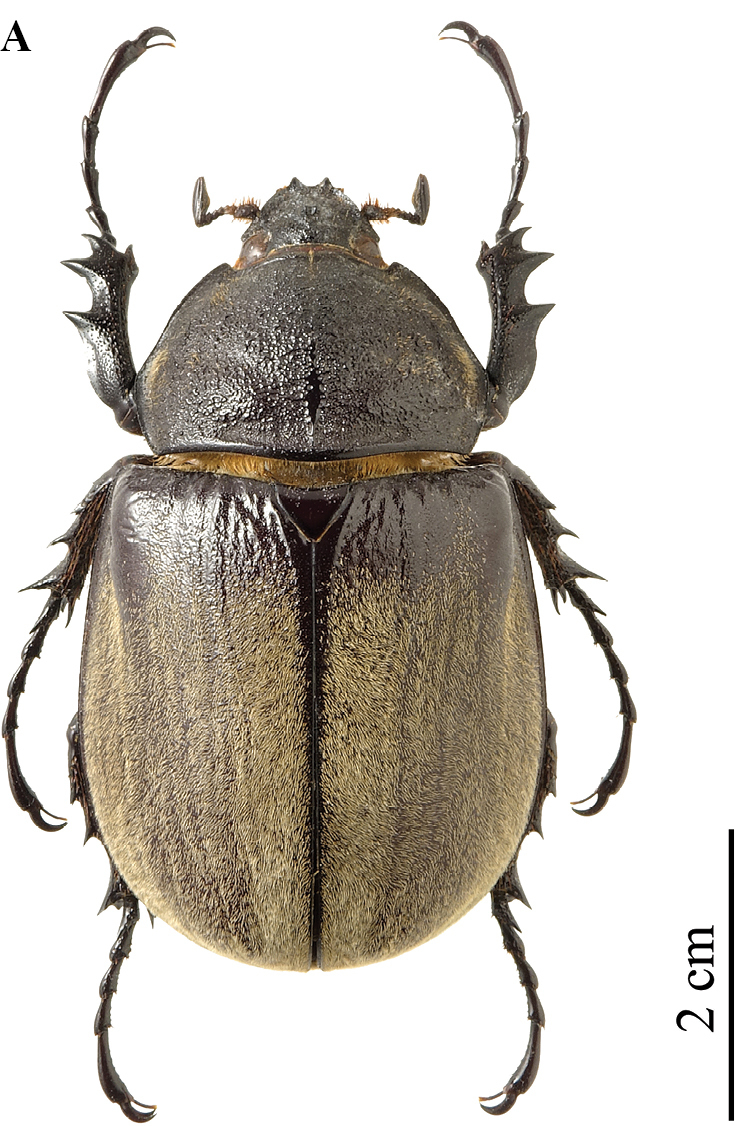
*Megasoma gyas* ♀ from Brazil, Rio Grande do Norte, Tenente Laurentino **A** dorsal habitus **B** double head's tubercle **C** pronotum with carina.

**Figure 14.**
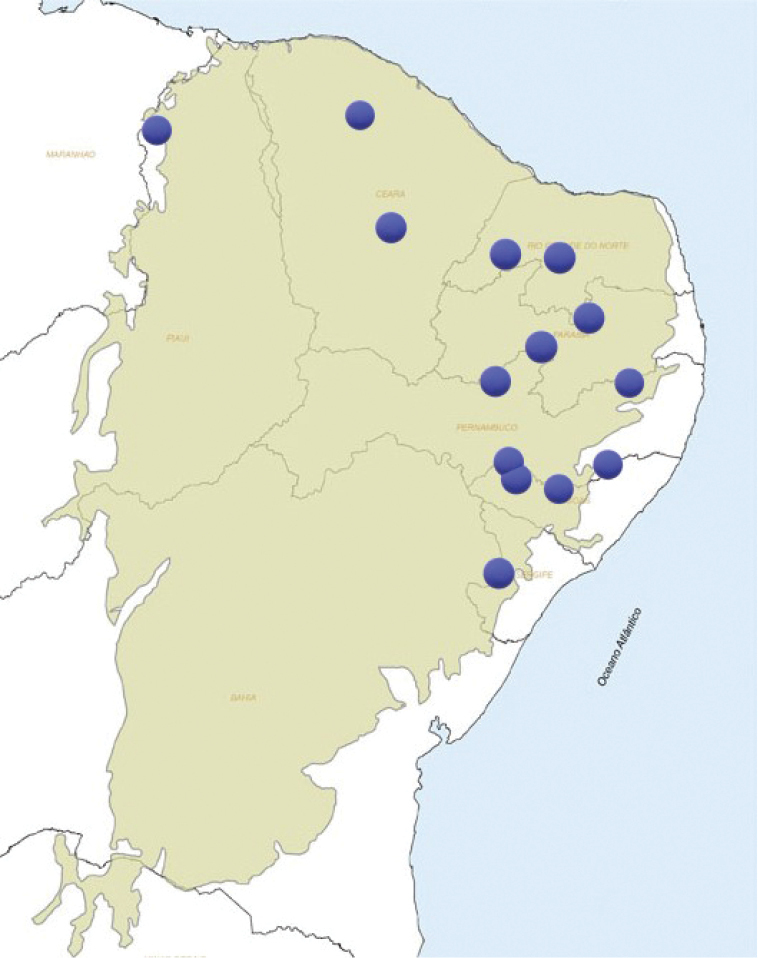
Map of Caatinga’s biome with collecting localities (grouped) of *Megasoma gyas* in northeastern Brazil.

#### Megasoma (Megasoma) typhon ssp.typhon

Taxon classificationAnimaliaColeopteraDynastidae

(Olivier, 1789)

7E9F975D-329C-540C-B14D-E1DF01FA5457

**Figure 15.**
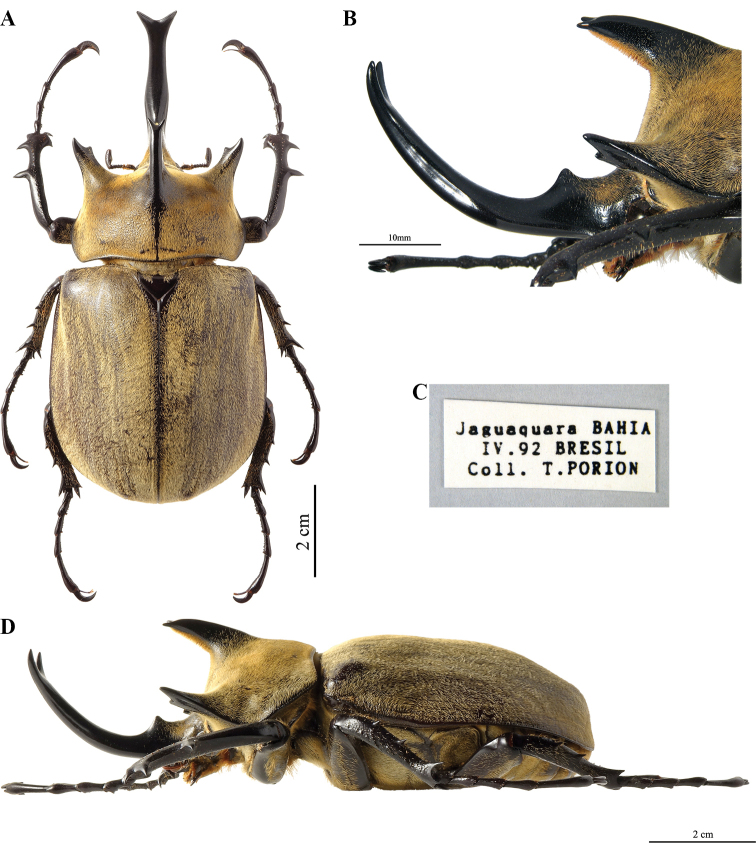
*Megasoma typhon typhon* ♂ from Brazil, Bahia, Jaguaquara **A** dorsal habitus **B** cephalic horn lateral view **C** original label **D** lateral habitus.

Figures 15A–D , 17A–C

Scarabaeus typhon Olivier, 1789: 12.
Scarabaeus entellus Olivier, 1792, syn. nov.
Scarabaeus monoceros Weber, 1801, syn. nov.
Megasoma gyas porioni Nagai, 2003, syn. nov.

##### Type material.

The holotype, i.e., the specimen illustrated by Olivier (1789: Tav. XVI fig. 252; reproduced in Fig. 5) is probably lost (Kobayashi, pers. comm., 2019). The designation of a neotype does not seem necessary since the species is well characterized and a search for the type in historical collections is still ongoing.

##### Distribution.

As explained above, the classical “long-horned” beetle up to now called *M. gyas gyas*, is actually a distinct species, *M. typhon* (Olivier, 1789). It occurs through the Mata Atlântica biome along the coastal areas of the Brazilian states of Bahia, Espirito Santo, Rio de Janeiro, São Paulo, and Minas Gerais. The biome Mata Atlântica (Atlantic Rain Forest) occupies an area equivalent to 622,000 sq. miles, i.e., 13% of Brazilian territory, and consists mainly of forests that run along the coastline from the State of Rio Grande do Norte to the State of Rio Grande do Sul. Due to its high human population density, it is one of the most deforested areas of Brazil. Only 7% of its original vegetation remains, scattered over hundreds of mostly small fragments. The Mata Atlântica presents a diversified group of forest ecosystems and a variety of floristic structures connected to specific different climatic conditions, all of them enjoying the humid winds that blow from the ocean (Coutinho 2016). We have no records of *M. typhon* from Paraná state, being São Paulo state (Antunes et al. 2007; Luzzi et al. 2016) the southernmost record. This species shows an interesting variability in the shape of cephalic and thoracic horns, mainly in the flat or thin section of the former and in the tips of the latter. This variability however is found all over the distribution range of the species and therefore is an individual variability without a geographical meaning. Based on this new interpretation, *M. gyas porioni* Nagai is a synonym of *M. typhon typhon*.

##### Material examined.

More than 100 specimens (major ♂ 80%, minor ♂ 10%, ♀ 10%) from the following Brazilian states: **Bahia**, Jaguaquara, IV 1992 from coll. T. Porion (3 ♂ 3 ♀, MPC), same locality, IV 1997 (1 ♂, KKC); IV 1997 (1 ♂, LMC); IV 1993 “Holotype Megasoma gyas porioni Shinji Nagai, 2003. Collection of S. Hisamatsu. (Brazil) Bahia, near Jaguaquara, ca. 800 m in alt., from T. Porion” (1 ♂, EUMJ); Paratypes, same data (1 ♂, 1 ♀, EUMJ); Amargosa, 20 XI 1988 (3 ♂, MPC), V 1989 (1 ♀, MPC), 20 XI 1988 (1 ♀, MPC); Salobrinho, 2000 (1 ♂, MPC), Ilhéus, Salobrinho, light, Atlantic forest, 14 VII 2016 (1 ♂, 2 ♀, MPC); Arataca, III 2013 (4 ♂, MPC); Ilhéus, Faz. Aliança 28 I 2017, leg. Souza (2 ♀, MPC) II 2013, same locality, II2019 (6 ♂, MPC), Bahia, XII 2002 Ceplac (1 ♂, EPCG); Porto Seguro, VIII 1970 (2 ♂, EPCG); Olivença, VI 2003 leg. R. Koike (2 ♂, EPGC); Una, VIII 2003 (1 ♂, EPCG); Itamajú, 100 m. II 2006 (3 ♀, MPC); Itabuna, II 2013 (3 ♂, MPC), Bahia, Fazenda de Cacau 10 XII 2010 (1 ♂, MPC); Itacaré, III 2012 (1 ♂, MPC); Jequié, no data (1 ♂, EPCG): **São Paulo**, Ubatuba VI 2013 (1 ♂, MPC); “Province” of SP, no further data (2 ♂, MPC); **Rio de Janeiro**, Teresópolis, I 2002 leg. Izabel (1 ♂, 1 ♀, EPCG); Rio das Ostras, VI 2011 leg. Igor (2 ♂, EPCG); Guapimirim, II 1980 leg. H.R. Pearson (1 ♂, EPCG); Xerém, VI 1999, 18.VII.1992 (2 ♂, MPC), same locality, VI 2001 (1 ♀, MPC); Nova Iguaçu, Res. Bio Tinguá VI 2009 leg. J.R. Mermudes (2 ♂, CEMT); **Espirito Santo**, Linhares II 1966 (1 ♂, MPC); **Minas Gerais**, Ipatinga, V 1987 leg. E.& P. Grossi (3 ♂, EPCG), same locality, V 2010, III 2016 (2 ♂, MPC), IV 1985 (3 ♂, LMC), V 1987 (1 ♂, LMC); V 2001, III 2016 (2 ♀, MPC), V 1995 leg. E.J. Grossi (1 ♂, 1 ♀, CEMT); Vale do Rio Doce, IV 1990 (1 ♀, MPC); Cataguases, VI 1995 leg. F.Z. Vaz-de-Mello (1 ♂, CEMT).

**Figure 16.**
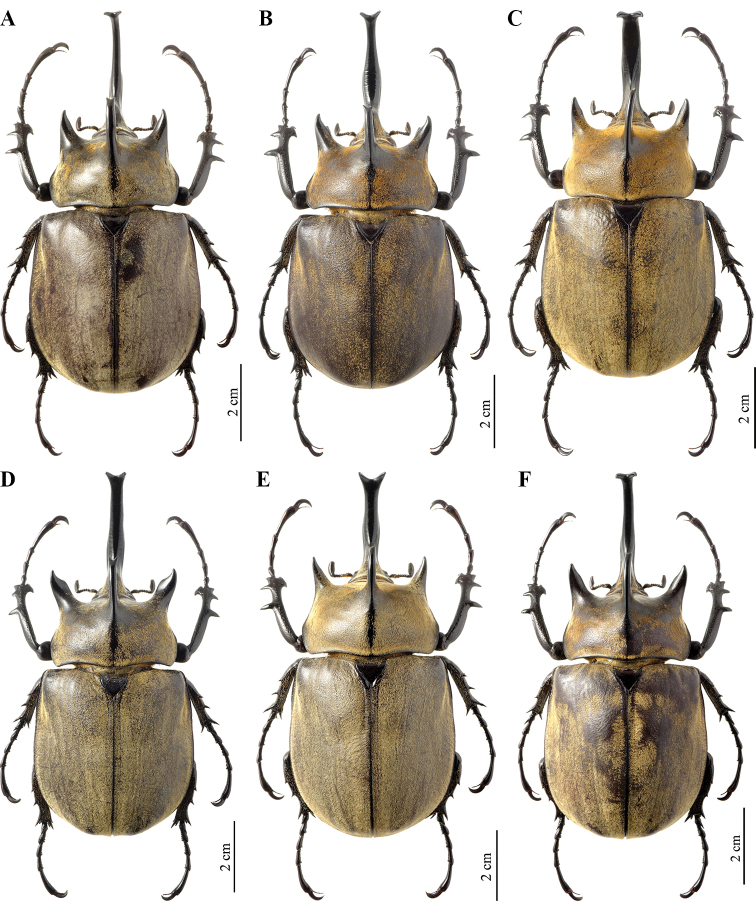
*Megasoma typhon typhon* gallery from different localities **A** Brazil, Bahia, Salobrinho **B** Brazil, São Paulo Province **C** Brazil, Bahia, Arataca **D** Brazil, Minas Gerais, Ipatinga **E** Brazil, Bahia, Jaguaquara **F** Brazil, Rio de Janeiro, Teresópolis.

**Male (Fig. 15).** The description below is based on a specimen from Bahia state, Jaguaquara locality, that closely resembles the specimen illustrated by Olivier. Other specimens from different localities are shown to illustrate the morphological variability constantly found all over the distributional range of the species (Fig. 16A–F). ***Size*.** L: 79 mm; TL: 108 mm; PL: 23 mm; PW: 37 mm; EL: 54 mm; EW: 49 mm; CL: 38 mm; PH: 10.5 mm. ***General appearance*.** Uniformly ebony brown covered by a yellowish short, fine, uniform, pilosity; head, including horn, consistently black except for the basal part near pronotum with sparse bristles. Tips of thoracic horns glossy black. ***Head.*** Cephalic horn long, projecting forwards and slightly curved upwards. In dorsal view, wider base, decreasing for a length of 6 mm and then gradually widening to a median flattener zone with a maximal width of 5.5 mm, then decreasing again for 13 mm, and finally gradually broadened towards forked apex. Apex always V-shaped, with divergent tips (Fig. 15A). This feature occurs always in minor, medium, and major males, with median or longer horns. Sometimes the tips of the cephalic horn, in dorsal view, are slightly bent backwards, mostly in medium or small specimens. The distance between the tips is 8.5 mm. The sides are bordered with a weak rim easily detectable, from base to mid-length. The dorsal side at the base bears the relief of a distinct tooth, with a maximal height of 3.5 mm.

***Pronotum*.** Whole surface is covered by uniform, fine, dense, yellowish pubescence. Anterior angles projecting as elongate, sharp, divergent horns, distinctly bent outwards, basal width ca. 9.5 mm, length from base 10.5 mm, distance between apices of anterior horns 37 mm. Median thoracic horn longer than laterals, length 17 mm, straight, dorsal side with a glossy black line, ventral side of median horn with abundant fine pubescence. PL/TH ratio 2.190. ***Elytra*.** Covered by very fine, uniformly dense, yellowish pubescence except along sutural edge and lateral margins; EL/EW ratio 1.102. Sutural edge with glossy black stripe; three or four ridges almost equally spaced on each elytron, covered by pubescence. Elytra in lateral view bulging but gradually flattened towards the apex. L/EL ratio 1.462, showing an elongate feature of the body. ***Abdomen*.** Laterally covered with very fine, short, reddish brown pilosity, medially glabrous only in a small area of each sternite. ***Legs*.** Fore tibia slightly rounded inwards, inner edge strongly dilated at apex, FL 26 mm. Anterior edge of protibia V-shaped. Lateral edge with three strong teeth, decreasing in length from basal to apical teeth, but basal tooth longer than subapical; basal tooth more distant from subapical tooth than the latter from apical. Basal and subapical teeth large, thick, sharp, triangular, pointing rearwards; apical tooth very reduced, pointing forwards. Inner apical spur strongly curved ventrally, distinctly longer than the apical tooth. TF 29 mm. ***Aedeagus*.** Overall appearance of the parameres more massive than in *M. gyas*, subrectangular, not narrow, as showed in Fig. 31C, D. Anterior phallobase also bigger.

##### Variation, males.

As usual, the development of cephalic and thoracic horns is allometric, but in medium and small specimens of *M. typhon typhon* with shorter cephalic horn, thoracic horns remain well developed. The tooth on the dorsal side of the cephalic horn is always present, in major, medium, and small specimens. Minor males in lateral view, often show a rounder feature of the body.

##### Measurements.

L: 57–85 mm; TL: 65–119 mm; PL: 17–24 mm; PW: 29–40 mm; EL: 41–57 mm; EW: 35–50 mm; CL: 13–38 mm; FL: 19–26 mm; TF: 22–30 mm.

**Figure 17.**
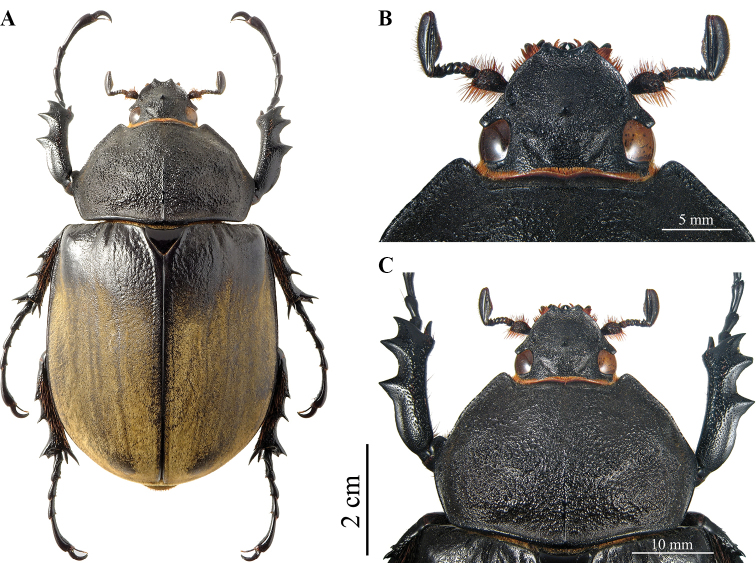
*M. typhon typhon* ♀ from Brazil, Minas Gerais, Ipatinga **A** dorsal habitus **B** single head’s tubercle **C** pronotum with carina.

**Female (Fig. 17). *Size*.** L: 73 mm; PL: 20 mm; PW: 32 mm; EL: 49 mm; EW: 41 mm.

***General appearance*.** Uniformly black; elytra covered with yellow-brownish dense pilosity for 4/5 of the total surface. ***Head*.** Middle of fronto-clypeal suture with single tubercle. ***Pronotum*.** Dull, coarsely punctate-rugose, strongly convex; posterior median carina 12 mm long, more than ½ total length. Anterior angles projecting, obtuse, with blunt tip. ***Elytra*.** Punctate-rugose glossy black on dorsal, anterior area, extending for 10 mm, almost 1/5 of the EL; sculpture finer towards pubescent surface. Pubescent surface consistently covered, with clearly visible longitudinal ridges, three or four ridges for each elytron, not equally spaced. Sutural line and epipleura glabrous, glossy black, with very fine punctation. ***Abdomen*.** Finely punctate, covered by short, brown-yellowish pilosity except for median central portion on sternites III-V. ***Legs*.** Fore tibiae shorter than in males, shorter than tarsi, 17 mm long, fairly arcuate, with three lateral strong teeth. Basal and subapical teeth equal in length; apical tooth smaller. Inner side with slight dilatation at apex. Inner spur curved ventrally almost equal in length as apical tooth. Lateral and inner apical teeth smaller than in males. TF length 22 mm.

##### Measurements.

L: 47–78 mm; PL: 12–21 mm; PW; 20–34 mm; EL; 29–50 mm; EW: 26–44 mm; FL: 11–19 mm; TF: 16–24 mm; HL: 5–11 mm.

**Figures 18, 19.**
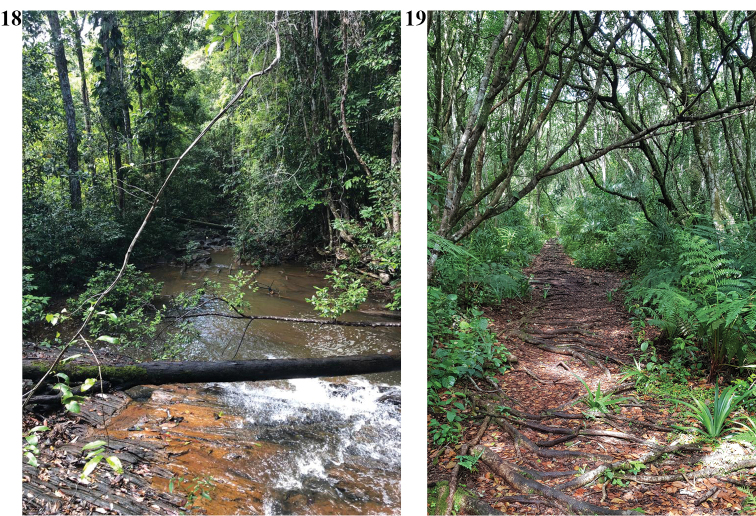
**18** View of Mata Atlântica biome, native trail, Itacaré, Bahia. (photograph L. Migliorati). **19** Trail into Mata Atlântica, Serra do Mar, Santa Catarina (photograph M. Prandi).

#### Megasoma (Megasoma) typhonssp.prandii

Taxon classificationAnimaliaColeopteraDynastidae

Milani, 2008

6528C64F-A52D-5B92-8909-CF19422BA12D

**Figure 20.**
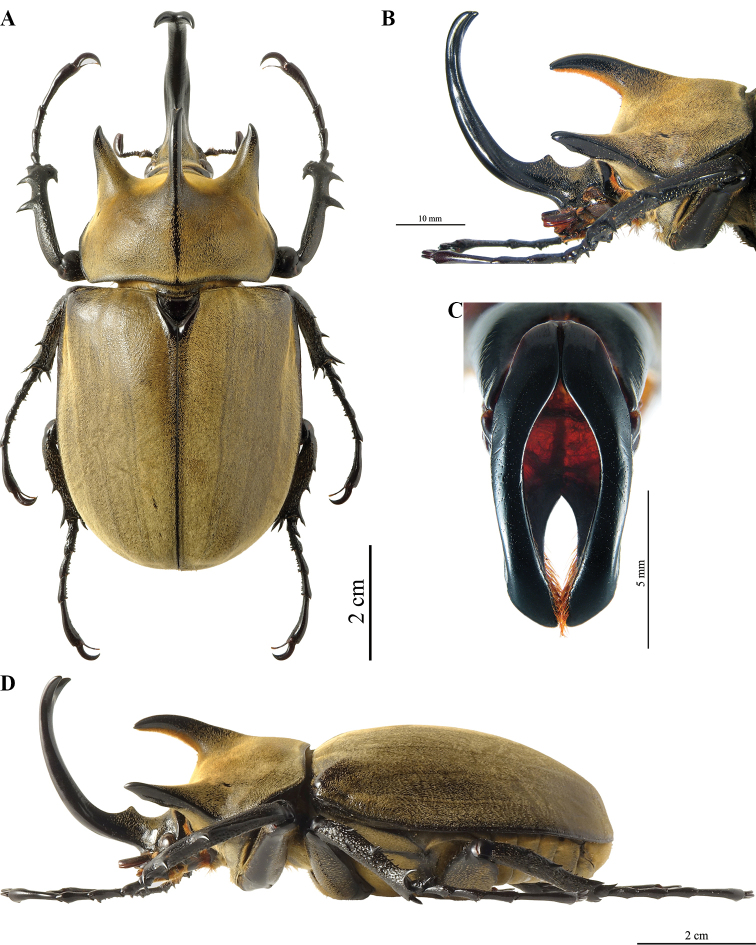
*M. typhon prandii* ♂ from Brazil, Santa Catarina, Serra do Mar **A** dorsal habitus **B** cephalic horn lateral view **C** aedeagus **D** lateral habitus.

**Figure 21.**
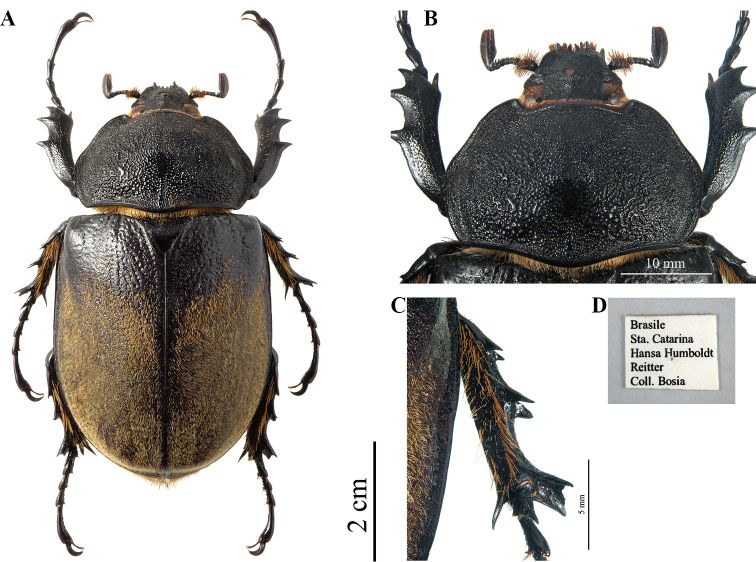
*M. typhon prandii* ♀ from Brazil, S. Catarina, Hansa Humboldt (now Joinville) **A** dorsal habitus **B** single head’s tubercle and pronotum **C** mesotibia with carina **D** label.

Figures 20A–D , 21A–D

Megasoma (Megasoma) gyas ssp. prandii Milani, 2008: 119–133.

##### Distribution.

This is the southernmost subspecies, nowadays restricted to the Serra do Mar region in the northern part of the Santa Catarina state. It displays a constant distinct morphology with respect to *M. typhon typhon*, which in addition to its geographic isolation, allows us to consider this population as a distinct subspecies.

##### Material examined.

***Holotype*** (male): “Brasile, Santa Caterina, Vale do Rio Itajaí, Timbó, 27 III 1989 local collector lgt., L. Milani det. 2008” (MPC). Paratypes: same data (4 ♂, MPC, 1 ♂, LMC, 1 ♂, MZC); same locality, 26 III 1989 (1 ♂, MPC); 28 III 1989 (1 ♂, MPC); 29.III.1989 (1 ♂, MPC); 6 IV 1989 (1 ♂, MSNM); 7 IV 1989 (1 ♂, MPC); 22 IV 1989 (1 ♂, 1 ♀, MPC). **Additional material examined**: Brazil, **Santa Catarina**, Rio dos Cedros, 2010 (1 ♂, UNLP); Hansa Humboldt, from the Collection Reitter (1 ♀, UBC); Joacaba, 1981 leg. Hartmann (1 ♀, UBC). **Rio Grande do Sul**, Porto Alegre, X 1946 (1 ♂, UBC); Porto Alegre, 1938, with label “*Typhon*” (1 ♂, UBC).

##### Remarks.

The diagnosis below is based on a specimen from Serra do Mar, near Rio dos Cedros, above 180 m a.s.l., caught in 2010. After this date very few specimens have been found, suggesting that the subspecies could be threatened by the reduction of its habitat.

##### Male diagnosis

**(Fig. 20).** A large *Megasoma*, uniformly dark brown, covered by a yellowish short, fine, uniform, pubescence; head, including horn, consistently black. Elongate body. ***Size*.** L: 75 mm; TL: 100 mm; PL: 21 mm; PW: 34 mm, EL: 52 mm; EW: 44 mm; CL: 39 mm; FL: 24 mm; TF: 25 mm. ***Head*.** Cephalic horn long, projecting forwards and noticeably curved upwards. In dorsal view, slightly wider at the base and apex, but remaining almost subrectangular laterally, without median flattened zone. Apex always V-shaped, with divergent tips (Fig. 20A). ***Pronotum*.** Whole surface covered by uniform, fine, dense, yellowish pubescence. Anterior angles projecting as elongate, sharp, weakly divergent horns. Median thoracic horn longer than laterals, straight, dorsal side with a glossy black line. ***Elytra*.** Covered by very fine, dense, uniform, yellowish pubescence except for sutural edge and epipleura; in lateral view not bulging, uniformly flattened towards apex. ***Legs*.** Fore tibia slightly rounded inwards, the inner edge strongly dilated apically. Anterior hedge of protibia V-shaped. ***Aedeagus*.** It differs from the nominative subspecies for the parameres of tegmen smoother and rounder laterally, as in Fig. 20C.

##### Variation, males.

A feature of the apex of the cephalic horn is that it is always V-shaped. A distinct tooth present on the dorsal side of the cephalic horn is always visible, also in medium and minor ♂. A feature of the body, in lateral view in medium specimens is that it is almost flat, and only very small specimens sometimes show a rounded body.

##### Measurements.

L: 53–78 mm; TL: 62–102 mm; PL: 15–23 mm; PW: 26–36 mm; EL: 33–53 mm; EW: 35–46 mm; CL: 11–40 mm; FL: 17–26 mm; TF: 19–27 mm),

##### Female diagnosis

**(Fig. 21).** A medium-large female of *Megasoma*, uniformly black, elytra covered for 5/6 of total surface by dense yellow-brownish pilosity. ***Size*.** L: 62 mm; PL: 17 mm; PW: 29 mm; EL: 42 mm; EW: 34 mm; FL: 17 mm; TF: 19 mm; HL: 8 mm. ***Head*.** Middle of fronto-clypeal suture with a single tubercle. ***Clypeus*.** Anterior lateral angles projecting into a very small tooth directed forwards and upwards; in this ssp. the two small teeth are distinctly more acuminate and curved upwards than in other species. ***Pronotum*.** Dull, except for the sides, with sparse bristles. Strongly convex, coarsely punctate-rugose; with posterior median carina flat, enlarged, smooth. Pronotum more expanded longitudinally and rounded laterally than in other species (Fig. 21A). ***Elytra*.** Punctate-rugose on dorsal, anterior area, glossy black; sculpture steadily finer towards pubescent surface. Pubescent surface rather sparse, roughly covered, with no visible longitudinal ridges. ***Legs*.** Fore tibiae shorter than tarsi, fairly arcuate, with three strong lateral teeth. On mesotibiae and metatibiae lateral teeth evolving into evident lateral carinae (Fig. 21C); lateral spiny processes extending up to 3 mm laterally, immediately before tarsi junction.

##### Measurements.

L: 49–65 mm; PL: 13–18 mm; PW: 21–29 mm; EL: 32–43 mm; EW: 29–36 mm; FL: 13–19 mm; TF: 15–20 mm; HL: 7–9 mm).

#### Megasoma (Megasoma) hyperion sp. nov.

Taxon classificationAnimaliaColeopteraDynastidae

275B9FFF-2294-5F6B-88E1-53164F9C214C

http://zoobank.org/F291D96E-BE88-4ECC-A64F-BEFCE2C0245B

**Figure 22.**
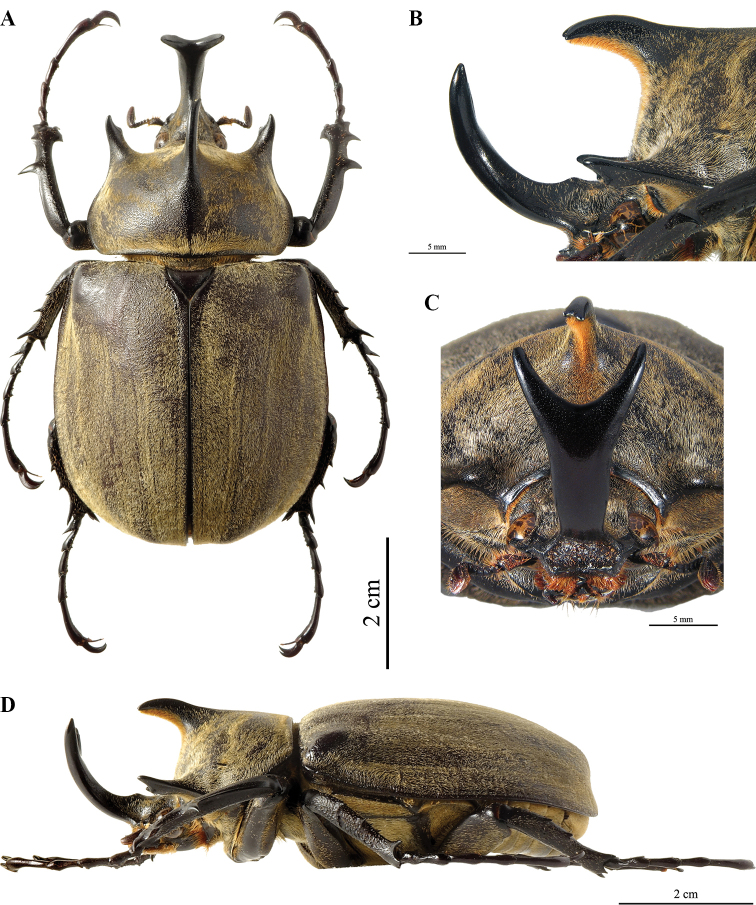
*Megasoma hyperion* sp. nov., holotype from Brazil, Minas Gerais, Águas Vermelhas ♂ **A** dorsal habitus **B** cephalic horn lateral view **C** cephalic horn frontal view **D** lateral habitus.

Figures 22A–D , 25A–C

##### Type material.

***Holotype*** ♂ (deposited in CERPE): Brazil, **Minas Gerais**, Águas Vermelhas, IV 2006. Paratypes (154 in total, 86 major ♂ [> 65mm]), 30 minor ♂ and 38 ♀), as follows: 1 paratype (allotype) ♀ same locality of holotype, III 2002 (CERPE); **Minas Gerais**, Salinas, V 2002 (1 ♂, CEMT); same locality, 2007 (1 ♂, CEMT); same locality, IV 2005 (1 ♂, 1 ♀, EPCG); Águas Vermelhas, V 2006 (1 ♂, MSNM); same locality, VI 1992 (1 ♂, EPCG); same locality III 2001 (1 ♂, EPCG); same locality, IV 2001 (8 ♂, 1 ♀, EPCG); same locality, III 2002 (5 ♂, EPCG); same locality, IV 2005 (1 ♂, MPC); same locality, V 2005 (1 ♂, 1 ♀, MPC); same locality, IV 2006 (35 ♂, EPCG, 11 ♂, 2 ♀, MPC); same locality, V 2006 (27 ♀, EPCG, 5 ♂, MPC); same locality, V 2008 (1 ♀, MPC); same locality, IV 2013 (18 ♂, EPCG); same locality, XII 1994 (2 ♂, KKC); same locality, III 2013 (1 ♂, MPC); same locality, IV 2013 (3 ♂, MPC); same locality , IV 2014 (1 ♂, KKC); same locality , VI 2014 (2 ♂, MPC); same locality , III 2016 (2 ♂, MPC); same locality , III 2018 (7 ♂, MPC); same locality , IV 2019 (4 ♂, MPC); Paracatú, VI 1981 (1 ♂, EPCG); Jaíba, V 1997 (1 ♂, EPCG); Montes Claros, II 2000 (1 ♂, EPCG); same locality, IV 2000 (1 ♀, EPCG); Capitólio, IV 2004 (1 ♂, EPCG); **São Paulo**, Boituva, VII 1991 (2 ♂, EPCG, 1 ♂ MPC).

##### Description of the holotype

**(Fig. 22). *Size*.** L: 70 mm; TL: 79 mm; PL: 18 mm; PW: 32 mm, EL: 43 mm; EW: 42 mm, CL: 22 mm; TH: 8 mm; FL: 21 mm; TF: 24 mm. ***General appearance*.** Uniformly dark ebony brown covered by sometimes dense, sometimes sparse rough pilosity; pubescence that turns from grey to yellowish brown color. Head, including horn, consistently black; base of horn towards pronotum with sparse bristles. Tip of pronotal horns, sutural and lateral edges of elytra and thorax shiny black as in legs. ***Head*.** Cephalic horn short, projecting forwards and curved upwards in lateral view. In dorsal view, narrower at base, 3 mm, and gradually broadens to a maximum of 7 mm towards the apex. Apex distinctly forked, V-shaped, distance between tips 10 mm (Fig. 22C). Sides bordered with weak rim from base to apex, rim detectable on total length. Dorsal side at base with small but evident triangular tooth. In lateral view, apex of tooth blunt, projecting upwards; height of tooth from base 1 mm. ***Clypeus*.** Anterior edge slightly concave, less concave than in *M. gyas*, broader than width of cephalic horn at base, lateral angles with small tooth, projecting forward, surface punctate with sparse bristles. ***Mandibles.*** Each with two small lateral teeth. In ventral view, interocular minimum width (IW) 4 mm, transverse eye diameter (TE) 4.5 mm, IW/TE ratio 1.154. Antennal club, in dorsal view, 3.8 mm of length. ***Pronotum*.** Completely covered by rough pubescence, with distal part quite abraded. Anterior angles projecting as sharp, elongate, parallel horns, slightly bent outwards; width at base ca. 4.9 mm, length 8 mm, distance between apices of anterior horns 21 mm. Median thoracic horn longer than laterals, sickle-shaped, 11 mm long. PL/TH ratio 2.250. L/PL ratio 3.888, higher than in *M. gyas*. ***Scutellum*.** Subtriangular, 5 mm long, 7 mm wide, largely coarsely punctate, lateral edges and lower apex smooth. ***Elytra.*** Covered by rough pubescence, except for black glossy punctation around scutellum, along epipleura and elytral suture; EL/EW ratio 1.023. Pubescence quite abraded on the anterior part of elytra, close to pronotum. Sutural punctate black stripe limited by very fine, visible ridges, covered by pubescence; three or four ridges, almost equally spaced, on each elytron. Elytra in lateral view not bulging, with flat feature declining towards apex. L/EL ratio 1.628, significantly higher than in *M. gyas*, with bulker body and shorter, broader elytra. This provides *M. hyperion* sp. nov. with an obviously more “squared” feature. ***Pygidium*.** Convex, covered by yellowish pubescence. ***Abdomen*.** Laterally covered with very fine, short, yellowish brown pilosity, medially on sternites III-V almost glabrous. ***Legs*.** Fore tibia almost straight, inner edge rather dilated inwards at apex, 21 mm of length. Anterior edge V-shaped, just over first tarsomere. External sides of tibiae with three teeth, decreasing in length from basal to apical tooth; basal tooth more distant from subapical than the latter from apical. Basal and subapical teeth large, sharp, triangular, pointing rearwards; apical tooth sharp, pointing forwards. Inner apical spur strongly curved ventrally, as long as the basal tooth. Fore tarsus 24 mm of length. Mesotibia and metatibia with three very pointed teeth increasing in length from basal to apical teeth; first tarsomere in middle and hind tarsi very acute. ***Aedeagus*.** Intermediate between *M. gyas* and *M. typhon*, more massive than the former, shorter in frontal view and less massive with parameres laterally more rounded than the latter, as shown in Fig. 31E, F. **Labels.** 1 (white): “Brazil, Minas Gerais, Aguas Vermelhas IV 2006”; 2 (red): “*Megasoma hyperion* sp. nov. / Holotypus ♂ / M. Prandi, P.C. Grossi & F.Z. Vaz-de-Mello det. 2020”.

##### Paratype variations, males.

The overall morphology is quite homogenous. Proportional to the body, the measured differences in CL are slight. The shape of the cephalic horn shows the most interesting variability, e.g., more elongate vs. more squared; triangular shape vs. subrectangular, even if all the paratypes always maintain the same V-shaped apex with regular/short tips, in both small and large specimens. The median thoracic horn is always longer than the laterals, often sickle-shaped as in holotype, but sometimes elongate as in other paratypes. The color of the pubescence changes from grey to yellowish to reddish brown. The elytra appear flat in lateral view in major and medium specimens, but small paratypes show an accentuated rounder body.

**Figure 23.**
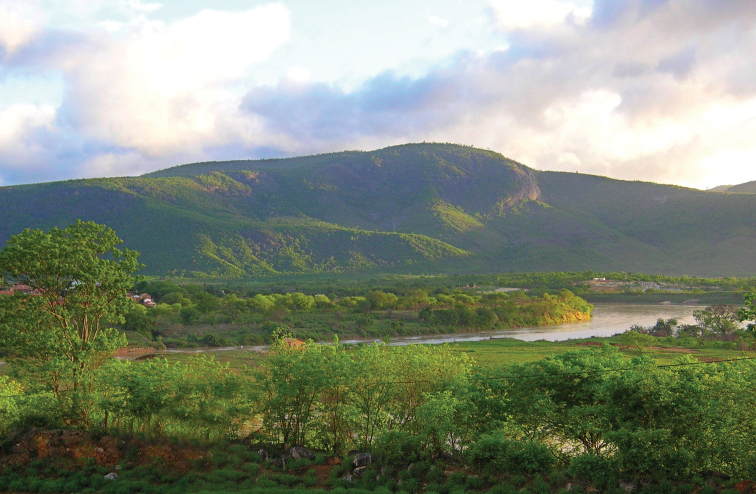
Panorama in Ponto dos Volantes, north of Minas Gerais (photograph P. Grossi).

##### Measurements.

L: 45–71 mm; TL: 52–94 mm; PL: 14–24 mm; PW: 22–37 mm; EL: 34–52 mm; EW: 14–47 mm; CL: 5–25 mm; TH: 4–9 mm; FL: 14–25 mm; TF: 18–26 mm.

**Figure 24.**
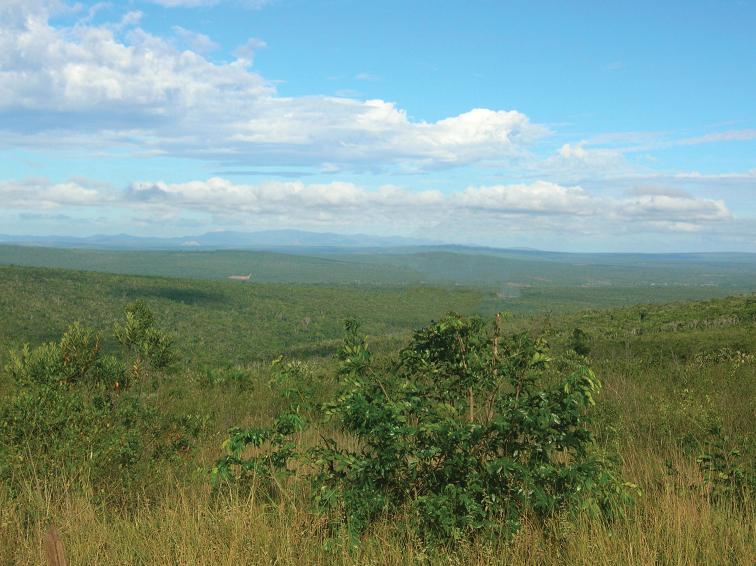
Panorama of north of Minas Gerais, near Aguas Vermelhas, with enclaves of Cerrado and Caatinga biomes (photograph P. Grossi).

##### Description of the female

**Figure 25.**
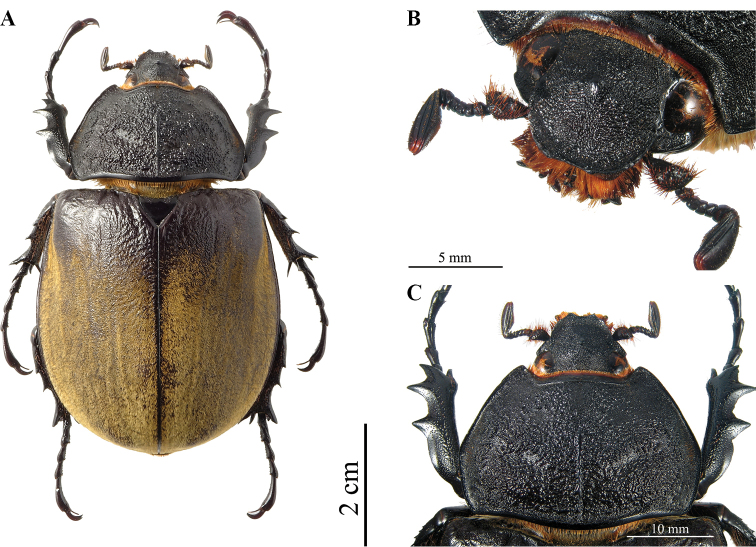
*Megasoma hyperion* sp. nov. from Brazil, MG, Águas Vermelhas, allotype ♀ **A** dorsal habitus **B** single head’s tubercle **C** pronotum with carina.

**(allotype) from Brazil, Minas Gerais (Fig. 25). *Size*.** L: 64 mm; PL: 18 mm; PW: 27 mm; EL: 42 mm; EW: 38 mm; HL: 8 mm. ***General appearance*.** Uniformly black; elytra with 3/4 of its surface covered by yellowish brown dense recumbent pilosity. ***Head*.** The middle of fronto-clypeal suture with a single tubercle. In ventral view, inter-ocular distance length 3.4 mm; transverse eye diameter width 3.5 mm. ***Clypeus*.** Finely punctate; anterior lateral angles projecting into a tooth directed forwards and upwards; distance between tips 2.5 mm; apical edge between the angles concave. ***Pronotum*.** Dull, coarsely punctate-rugose, strongly convex; posterior median carina 11 mm long, more than ½ of total length. Anterior angles projecting, obtuse, with blunt tips. ***Scutellum*.** Triangular, smooth, shiny, impunctate. ***Elytra*.** Surface glossy black, punctate-rugose anteriorly, near base coarser; punctate surface extending for 12 mm in length, almost 1/5 of EL. Elytral pilosity very uniform, yellowish brown, with easy detectable longitudinal ridges, three or more for each elytron, almost equidistantly spaced out. Dorsal sutural line and lateral edges glossy black, with very fine punctation. As in males, the distinct shape of a stocky body is a visible differential character (Fig. 25A). ***Pygidium*.** In lateral view, concave, with very fine punctation. Surface in basal half-covered with short, fine, grey pubescence; in apical half with scattered, erected, brown-yellowish setae. ***Abdomen*.** Sternites very finely punctate, covered by short, yellowish brown pilosity, except for a small central portion in the middle of sternites III-V. ***Legs*.** Protibiae shorter than tarsi, shorter than in the males, tarsi shorter too; FL 17 mm, TF 20 mm. External sides with three strong teeth almost equidistant. Basal and subapical teeth almost equal in length; the apical tooth smaller. Inner side without a strong dilated apex. Inner spur curved ventrally and shorter than apical tooth. On mesotibiae and metatibiae three lateral sharp teeth, with the subapical and the apical weakly evolving in lateral carinae, with presence of basal embryonic spiny processes.

##### Measurements of female paratypes.

L: 48–71 mm; PL: 14–20 mm; PW: 21–33 mm; EL: 32–48 mm; EW: 27–38 mm; FL: 11–17 mm; TF: 16–20 mm; HL: 6–8 mm.

##### Bionomics.

Very little information is available on this beetle’s behavior. The beetle flies usually from 9–10 p.m. till 2 a.m. and it is attracted by white mercury lights. Males are encountered more frequently (EJ Grossi, pers. comm., 2019).

##### Etymology.

Noun in apposition. Following the example of Jablonsky who chose the name of a Titan, Gyas, we also follow Greek mythology and choose the name of another Titan, Hyperion, son of Uranus (the sky) and Gaia (the earth).

##### Remarks.

Grossi et al. (2008) were the first to remark on the disjunct distribution range of (alleged) *M. rumbucheri* (see also Prandi 2016; Santos et al. 2013). The type locality of the new species falls within the “mata seca ou de cipó” (dry forest) habitat, a crossroad of the three biomes: Caatinga, Cerrado, and Mata Altlântica. An interesting historical record (1908) from Be-Kuwa (Kobayashi 2019) in the locality of Paranaíba (western Minas Gerais state, Brazil). The beetle’s external morphology is rather homogeneous, with the cephalic horn showing a certain degree of variability, but the species-specific characters here identified are constant and indicate this taxon as a separate species.

### Related South American taxa

#### Megasoma (Lycophontes) joergensenijoergenseni

Taxon classificationAnimaliaColeopteraDynastidae

(Bruch, 1910)

E6A84C6B-50FC-55C2-9945-6A179087C179

Figure 26

Lycophontes jörgenseni Bruch, 1910: 74.

##### Remarks.

**Figure 26.**
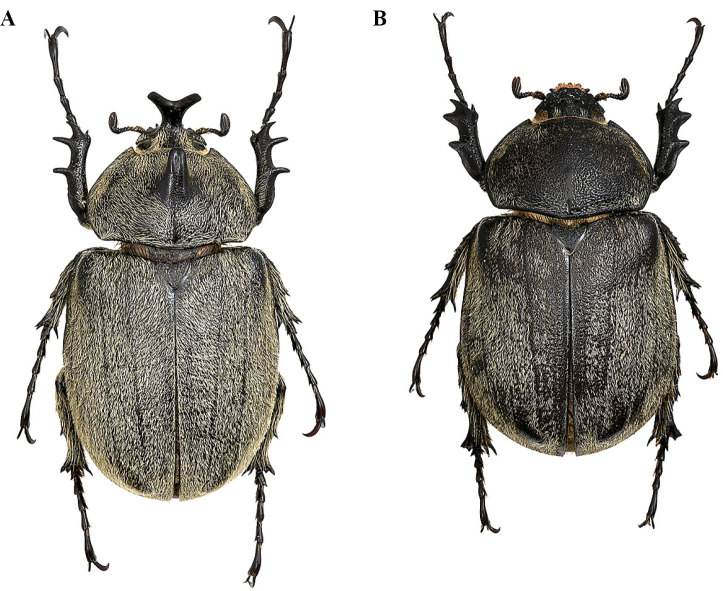
**A***M. joergenseni joergenseni* ♂ **B***M. joergenseni joergenseni* ♀.

The subgenus Lycophontes, of the genus *Megasoma*, is a group comprised of small-sized taxa. *Megasoma jorgenseni jorgenseni* is a completely-pubescent taxon, occurring in central-northern Argentina and southeastern Bolivia. The total body length varies from 30 to 40 mm (Fig. 26, courtesy of Mushi-sha). The original type of Bruch (San Luis, Mendoza) considered lost, was recently found by the Argentinean entomologist FC Penco in a private collection, and is now deposited in the UNLP collection.

#### Megasoma (Lycophontes) joergensenipenyai

Taxon classificationAnimaliaColeopteraDynastidae

Nagai, 2003

BE2FDAF7-0E21-59BF-ADF8-5E4CC62C16F3

Figure 27

Megasoma joergenseni ssp. penyai Nagai, 2003: 38–39.

##### Remarks.

**Figure 27.**
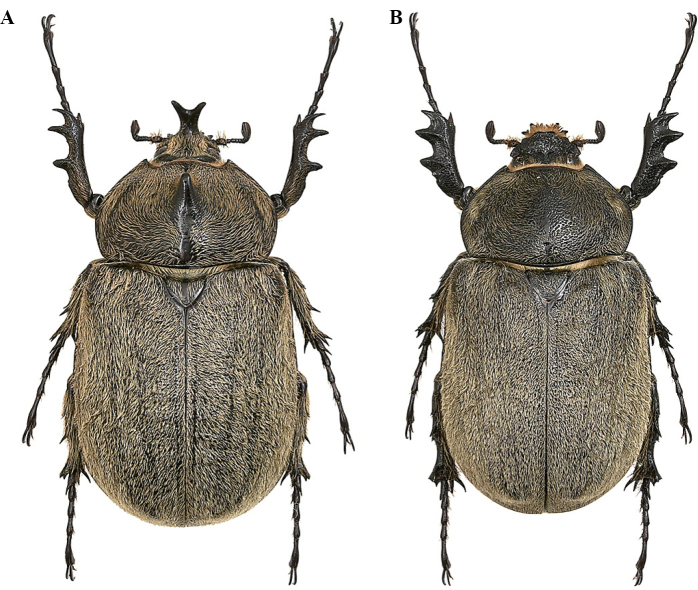
**A***M. joergenseni peynai* ♂ **B***M. joergenseni peynai* ♀.

The subspecies penyai is restricted to an area in central-western Paraguay (holotype from Loma Plata, Chaco). The main characters differentiating it from the subspecies joergenseni are found in the smaller median thoracic horn and in the denser pubescence, giving a more brownish color. It is also usually smaller than ssp. joergenseni. (Fig. 27, courtesy of Mushi-sha).

#### Megasoma (Megasoma) anubis

Taxon classificationAnimaliaColeopteraDynastidae

(Chevrolat in Guérin, 1836)

BDBD95F5-8734-5668-B549-7C046925722E

Figure 28

Scarabaeus anubis Chevrolat, 1836: t.139–140.
Scarabaeus hector Gory, 1836, synonymy by Burmeister 1847: 278–279.
Megalosoma theseus Laporte, 1840, synonymy by Burmeister 1847: 278–279.

##### Remarks.

**Figure 28.**
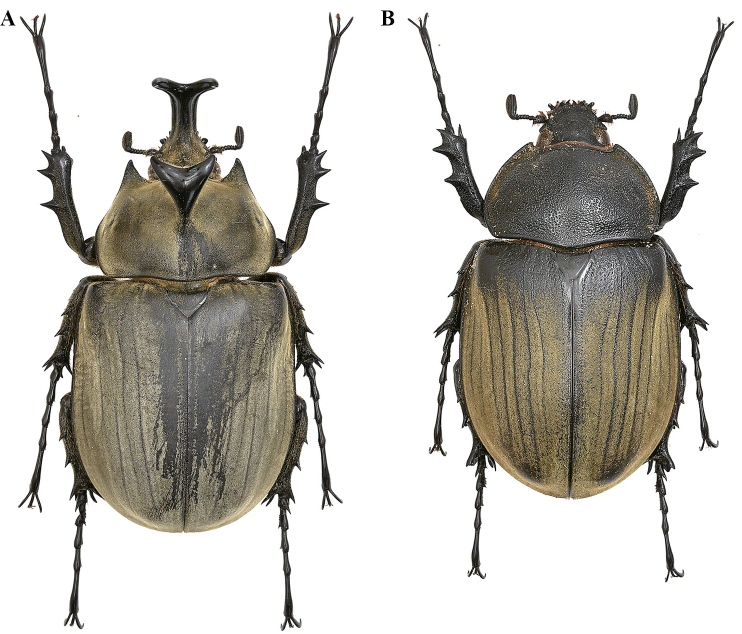
**A***M. anubis* ♂ **B***M. anubis* ♀.

This is another completely-pubescent South American taxon belonging to the genus *Megasoma*. It occurs in northeastern Argentina (Misiones region), southeastern Paraguay, and in eastern and southern Brazil (Espírito Santo, Rio de Janeiro, Paraná, Santa Catarina, and Rio Grande do Sul states). It is interesting to note that in the first two Brazilian states, in the region of Teresópolis, RJ, it is sympatric with *Megasoma typhon typhon*. The distinctive characters are the cephalic horn and the sickle-shaped thoracic horn, both short with a very wide bifurcated apex. Total body length varies from 50 to 90 mm (Fig. 28, courtesy of Mushi-sha).

#### Megasoma (Megasoma) hermes

Taxon classificationAnimaliaColeopteraDynastidae

Prandi, 2016

BC26F853-535F-57DA-A575-7F2745C123EC

Figure 29

Megasoma hermes Prandi, 2016: 525–584.

##### Remarks.

**Figure 29.**
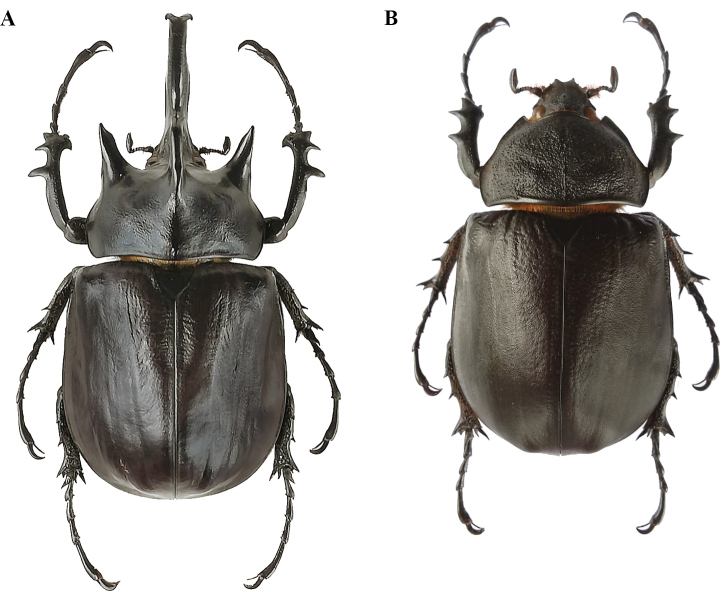
**A***M. hermes* ♂ **B***M. hermes* ♀.

The distribution range of this species (northern Brazil, on the border with Venezuela and Guyanas) represents the northernmost distribution compared to the aforementioned species and subspecies. It is likely that Endrödi’s (1977) and Morón’s (2005) claims of the alleged presence of *M. gyas* in Suriname and Guyana refer to this species. The validity of this taxon has been recently confirmed by further findings from Venezuela, at the border with Brazil (see Kobayashi, 2019). Only ten specimens have been collected thus far and a large specimen is deposited in MSNM. It is a completely glabrous *Megasoma* on its dorsal side. Ventrally it bears a very poor pubescence, mainly on female specimens. The variability of total body length varies from 68 to 105 mm (Fig. 29, courtesy Mushi-sha).

**Figure 30.**
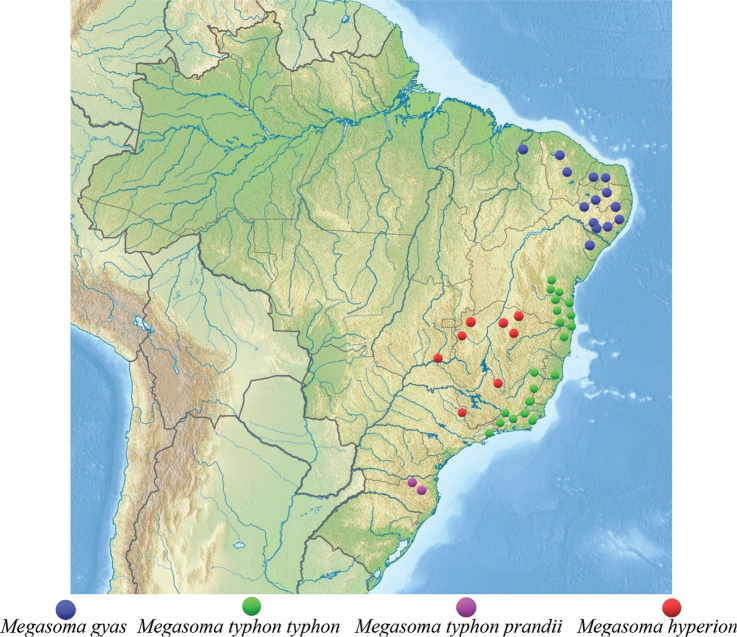
Collecting localities of the *Megasoma gyas* species-group.

**Figure 31.**
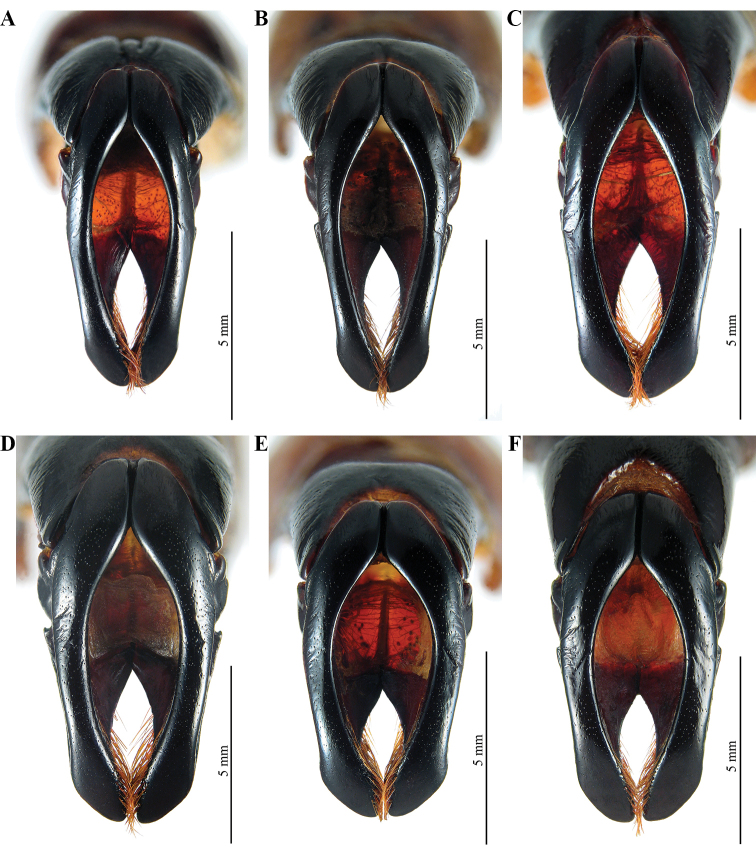
Frontal view of aedeagi **A** aedeagus of *M. gyas* from Brazil, Pernambuco, Custódia **B** aedeagus *M. gyas* from Brazil, Sergipe **C** aedeagus of *M. typhon typhon* from Brazil, Bahia, Jaguaquara **D** aedeagus of *M. typhon typhon* from Brazil, Minas Gerais, Ipatinga **E, F** aedeagi of *M. hyperion* sp. nov. from Brazil, Minas Gerais, Aguas Vermelhas.

### Identification keys for major males and females of the Megasoma (Megasoma) gyas species group. The identification of minor males requires the examination of the aedeagus.

**Males**:

**Table:** 

1	Dorsum covered by pubescence, pronotum three-horned	***M. gyas* species group 2**
–	Different combination of characters	**other *Megasoma* species**
2	Cephalic horn wide and short (< 30 mm)	**3**
–	Cephalic horn elongate, medium to long (> 30 mm)	**4**
3	Cephalic horn with apex U-shaped with long tips	***Megasoma gyas***
–	Cephalic horn with apex V-shaped with short tips	***Megasoma hyperion* sp. nov.**
4	Cephalic horn medium to long, straight	***Megasoma typhon typhon***
–	Cephalic horn long, curved upwards and backwards	***Megasoma typhon prandii***

**Females**:

**Table:** 

1	Female with a single cephalic tubercle	**2**
–	Female with two cephalic tubercles	***Megasoma gyas***
2	Sides of pronotum rounded	***Megasoma typhon prandii***
–	Sides of pronotum subtrapezoidal	**3**
3	Elongated body shape	***M. typhon typhon***
–	Stocky body shape	***M. hyperion* sp. nov.**

## Conclusions

Retracing the history of the taxon *Megasoma gyas* was more than a revision, it was a dip in the past. The historical part, with the various Fathers of Entomology who have studied this insect, was exciting. In an age without knowledge, and without modern scientific means, scientists like George Marcgraf, Carl Gustav Jablonsky, and Guillaume-Antoine Olivier were able to write and paint with an accuracy that seems miraculous. We want to dedicate this paper to those pioneers, hoping that it will be useful for the reader to clarify the taxonomic evolution and the correct classification of one of the most beautiful taxa of the Dynastini tribe.

## Supplementary Material

XML Treatment for Megasoma (Megasoma) gyas

XML Treatment for Megasoma (Megasoma) typhon ssp.typhon

XML Treatment for Megasoma (Megasoma) typhonssp.prandii

XML Treatment for Megasoma (Megasoma) hyperion

XML Treatment for Megasoma (Lycophontes) joergensenijoergenseni

XML Treatment for Megasoma (Lycophontes) joergensenipenyai

XML Treatment for Megasoma (Megasoma) anubis

XML Treatment for Megasoma (Megasoma) hermes

## Supplementary materials

Supplementary material 1 The taxa of the genus *Megasoma* Kirby, 1825 (Coleoptera, Scarabaeidae, Dynastinae) related to *M. gyas* (Jablonsky in Herbst, 1785) are revised. Data type COL Explanation note Megasoma (Megasoma) gyas is recognized as a monotypic species (confirming the invalid subspecies status of *M. gyas rumbucheri*, new synonymy) restricted to the Caatinga biome of Northeastern Brazil. The “long-horned *gyas*” is recognized as a separate polytypic species with the name Megasoma (Megasoma) typhon typhon (Olivier, 1789) (stat. nov.) for the populations occurring through the Mata Atlântica biome of Brazil, from Bahia to São Paulo states (*M. gyas porioni* Nagai is synonymized with *M. typhon typhon* new synonymy), and Megasoma (Megasoma) typhon prandii Milani, 2008 (new combination) for the population restricted to a small area in the state of Santa Catarina, South Brazil. The “short-horned *gyas*” occurring in Minas Gerais, São Paulo and southwestern Bahia is recognized as a separate new species and described as Megasoma (Megasoma) hyperion sp. nov. The work includes a substantial historical research and the redescriptions of the historical species. Additionally, distribution maps and a male and female key to the species in the species group are provided. https://binary.pensoft.net/file/481457This dataset is made available under the Open Database License (http://opendatacommons.org/licenses/odbl/1.0/). The Open Database License (ODbL) is a license agreement intended to allow users to freely share, modify, and use this Dataset while maintaining this same freedom for others, provided that the original source and author(s) are credited.Massimo Prandi, Paschoal C. Grossi, Fernando Z. Vaz-De-Mello
