# Supplementary material for: Revision of the Megasoma (Megasoma) gyas (Jablonsky in Herbst, 1785) species group (Coleoptera, Scarabaeidae, Dynastinae)
Source: Zookeys. 2020 Nov 30;999:109–45. doi: 10.3897/zookeys.999.53130 (PMC7723882; doi:10.3897/zookeys.999.53130)
Supplement: Supplementary material 1 — The taxa of the genus Megasoma Kirby, 1825 (Coleoptera, Scarabaeidae, Dynastinae) related to M. gyas (Jablonsky in Herbst, 1785) are revised. [file zookeys-999-109-s001.pdf]

Revision of the *Megasoma* (*Megasoma*) gyas (Jablonsky *in* Herbst, 1785) species group (Coleoptera, Scarabaeidae, Dynastinae)

by MASSIMO PRANDI*, PASCHOAL C. GROSSI° & FERNANDO Z. VAZ-de-MELLO^

**Via Del Seminario 16, I-25087 Salò, BS, ITALY . E-mail: prandi.m@libero.it (corresponding author)*
°Universidade Federal Rural de Pernambuco, Departamento de Agronomia/Fitossanidade, Laboratório de Taxonomia de Insetos, Programa de Pós-graduação em Entomologia Agrícola-PPGEA, 52171-900 Recife, Pernambuco, Brazil. E-mail: paschoal.grossi@gmail.com
^Universidade Federal de Mato Grosso, Instituto de Biociências, Departamento de Biologia e Zoologia, Coleção Entomologica, 78060-900 Cuiabá, Mato Grosso, Brazil. E-mail: vazdemello@gmail.com

Abstract
The taxa of the genus *Megasoma* Kirby, 1825 (Coleoptera, Scarabaeidae, Dynastinae) related to *M. gyas* (Jablonsky *in* Herbst, 1785) are revised. *Megasoma* (*Megasoma*) *gyas* is recognized as a monotypic species (confirming the invalid subspecies status of *M. gyas rumbucheri,* **new synonymy**) restricted to the Caatinga biome of Northeastern Brazil. The "long-horned *gyas*" is recognized as a separate polytypic species with the name *Megasoma* (*Megasoma*) *typhon typhon* (Olivier, 1789) (**stat. nov.**) for the populations occurring through the Mata Atlântica biome of Brazil, from Bahia to São Paulo states (*M. gyas porioni* Nagai is synonymized with *M. typhon typhon* **new synonymy**), and *Megasoma* (*Megasoma) typhon prandii* Milani, 2008 (**new combination**) for the population restricted to a small area in the state of Santa Catarina, South Brazil. The "short-horned *gyas*" occurring in Minas Gerais, São Paulo and southern Bahia is recognized as a separate new species and described as *Megasoma* (*Megasoma) hyperion* **sp. nov.** The work includes a substantial historical research and the redescriptions of the historical species. Additionally, distribution maps and a male and female key to the species in the species group are provided.

Key words: Cerrado, Neotropical region, new species, Scarabaeoidea, South America

Introduction
Megasoma (*Megasoma) gyas* (*Coleoptera, Scarabaeidae, Dynastinae*), locally known as "besouro de chifre" or "besouro com chifre" or "grande besouro", is perhaps the most interesting species among all the large sized South American *Megasoma*. Unlike its "naked" related species, i.e. the species of the *Megasoma actaeon* (Linnaeus, 1758) group, it displays a thick cover of short shiny setae on the whole dorsum, a feature shared with *M. anubis* (Chevrolat *in* Guérin, 1836) and *M. joergenseni* Bruch, 1910. Through the examination of type material as well as of large series of specimens from several localities, it was possible to re-define the species and to isolate three taxa which deserve a separate species or subspecies status. In this paper we describe a new species distributed in open Cerrado areas, as well as in some transitional areas of Cerrado and Caatinga, ranging from São Paulo to Bahia states. Additionally, we propose the use of the name *Megasoma* (*Megasoma) gyas* over *Megasoma rumbucheri*, considered here a new junior synonym of the former, and the name *Megasoma* (*Megasoma) typhon* (Olivier, 1789) over *M. gyas* for the current "long-horned *gyas*".

Material and Methods
A total of 328 specimens were studied (all wild collected), deposited in following collections:
BMNH: The Natural History Museum, London, UK (Maxwell Barclay)
EPGC: Everardo and Paschoal C. Grossi Collection, Nova Friburgo, Rio de Janeiro, Brazil

EUMJ: Ehime University, Entomological Department, Matsuyama, Japan (Hiroyuki Yoshitomi)
INPA: Instituto Nacional de Pesquisa da Amazônia, Manaus, Brazil (Márcio L. de Oliveira)
MPC: Massimo Prandi Private Collection, Salò, Italy
MSNM: Museo Civico di Storia Naturale, Milano, Italia (Fabrizio Rigato)
MPEG: Museu Paraense Emilio Goeldi, Belém, Brazil (Orlando Tobias)
DZUP: Universidade Federal de Paraná, Centro Politécnico, Curitiba, Brazil (Lucia Massutti de Almeida)
CEMT: Universidade Federal do Mato Grosso, Instituto de Biociências, Cuiabá, Brazil (Fernando Z. Vaz-de-Mello)
CERPE: Universidade Federal Rural de Pernambuco, Recife, Brazil (Paschoal C. Grossi)

Specimens were examined through naked eye observation, or/and with a stereomicroscope. Pictures were taken by using a digital camera Canon Powershot S50 on base Leica M5 and processed for the Focus Stacking with the Combine ZP software. Dissection of male genitalia was made by extraction with forceps through an aperture operated between tergite VI and the propygidium. The parameres were then glued on a card and pinned below the specimens. The distribution maps were made using facsimiles suitable for the purpose available on the web.

List of abbreviations of measurements:
L : body length from clypeal apex to elytral apex of males and females
TL: length from tip of cephalic horn to elytral apex
PL: pronotum maximum length
PW: pronotum maximum width
TH: lateral thoracic horn length from base
EL: elytral maximum length
EW: elytral maximum width
CL: cephalic horn length measured along the external curve
PH: lateral pronotal horn length from base
FL: fore tibia length
TF: fore tarsi length
HL: head length

NOMENCLATURAL AND TAXONOMIC HISTORY OF THE TAXON *GYAS*

Pre-Linnean accounts

....Dutch and French merchants mantained regular commercial contact with amerindians Tupi (coastal populations of Northeastern Brazil) from the end of the 16th and well into the 17th century. ....in doing so, they also had access to a variety of ethnographic items and natural species sold or given to them by the Tupi. These items were highly prized in the Dutch Republic. In the Low Countries from the end of the 16th century it became more and more fashionable to own a collection of curiosities. Members of the nobilty and, in increasing numbers intellectuals and the high bourgeoisie searched tirelessly for exotica from distant lands (Bergvelt and Kistemaker 1992). Items from Portoguese America soon made their way into Dutch collections....(Françoso 2012). In the year 1637 the Count Johan Maurits van Nassau-Siegen became the General Governor of land and see of the Dutch North and Northeast Brazil under the Geoctoyeerde Westindische Compagnie, (the Dutch West-Indies Company). He invited George Marcgraf (or Marcgrave) to Brazil for the first scientific expedition of the area, which was made between 1637 and 1644. During the expedition beautiful oil and watercolour plates, showing maps and scientific subjects, such as people, plants, animals and insects, were made by Albert Eckout, Zacharias Wagner and by George

Marcgraf himself. A big part of those plates were rejoined into the *Libri picturati* now housed in Krakow, Poland. Among the subjects of the "Handbook" (*Libri* principis) of Libri *picturati* made by Marcgraf there is a beautiful plate showing a *Megasoma gyas* (Fig. 1). It was the first time that this species was brought to the attention of the Western world.

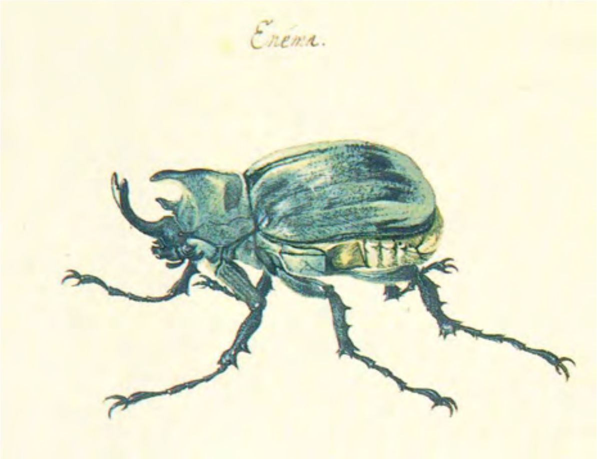

Fig. 1

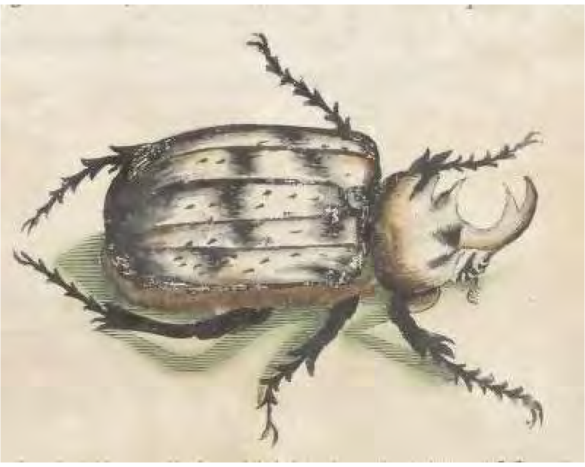

Fig. 2

Fig. 1. George Marcgraf, *Libri Picturati,* pag. 477 *Libri Principis*, before 1648, "*Enéma*" (indigenous name). **Fig. 2**. W. Piso & G. Marcgraf, Historia Naturalis Brasiliae 1648, pag. 246. *Enéma*, *Enena, Escaravelha Lusitanis, Taurus Volans*

Subsequently, another illustration, a poorly executed drawing of the same beetle, was provided in the work *Historia Naturalis Brasiliae* of 1648 by Piso and Marcgraf (Fig. 2). This plate was the one seen by Linnaeus (1758) and listed by him among the references he provided while describing *Scarabaeus actaeon* (Prandi 2018). Despite the poor quality of the drawing there is little doubt that it represents a *Megasoma* gyas, an interpretation supported by the accompanying text: "the body is covered by yellowish pilosity…the first section of the body is three-horned…". The chapter of the book dealing with *Megasoma gyas* is entitled "Enema rare conformationis" and the *Megasoma* beetle is there called "Taurus volans" (flying bull) together with other three beetles. Marcgraf's first xylography at page 246 (Fig. 2), and the related *guache* color image in *Libri picturati* (Fig. 1) show clearly a short-horned *Megasoma*: this is the most relevant character pointing towards *Megasoma gyas* s.str..
George Marcgraf (1610-1644) was a German scientist, who, at the beginning of the Dutch expedition had a role of simple attendant of the famous physician Wilhem Piso, but day after day thanks to his enthusiastic work gained the favor of the Dutch Governor. Four were the books on the traditional Brazilian medicine written by Piso, eight the books on the complex of natural sciences written by Marcgraf, *Historiae naturalium Brasiliae*, decoded by Johannes de Laet (1593-1649), one of the directors of the Dutch west india Company, all those reunited by J. Maurits van Nassau in the main opera *Historia Naturalis Brasiliae* (1648), financed by van Nassau himself, after his return to Holland and four years after Marcgraf's death. The work *Historia Naturalis Brasiliae,* until the beginning of XIX century, represented a source of important information on Brazilian natural history for European scientists, including Linnaeus, who referred to it in his *Systema Naturae*, often using Marcgraf's (or Piso's) descriptions as the only basis for establishing his species names. Marcgraf's drawings and descriptions of course referred to the places that Marcgraf himself had visited, i.e. the old Dutch "Captaincy" of Northeast, which includes the current States of Paraíba, Pernambuco, Alagoas, Ceará, Piauí and Rio Grande do Norte. Marcgraf visited certainly also the north of State of Bahia, having landed in Salvador (now its capital city). Hence it is very likely that the information given by Marcgraf on *Megasoma gyas* refers to specimens observed or collected

in those regions and then given to the Count J. Maurits.

Post-Linnean scientific reports

In 1785 Jablonsky described *Scarabaeus gyas* following the Linnean method: "...in the lower part of the head there is a horn, considerably wide but hidden, in the shape of a shovel, which the more it lengthens and the more it becomes wider, towards the end it ends in two long teeth…the armor is completely covered by yellow hair…in addition to this sickle-shaped horn the armor stretches downwards and both sides form a point…so it can also be called tricorn thorax…the elytra are thick and covered with yellow hair, which give to the insect an unusual sumptuous appearance…". The description is enriched by a precious color plate by Jablonsky himself.

Carl Gustav Jablonsky (1756-1787) was a German scientist and illustrator, private secretary of the Queen of Prussia. Jablonsky's plates are worldwide recognized among the most beautiful plates produced in the age of enlightenment. Although "Natursystem alles bekannt...." is attributed to J. F. W. Herbst (1743-1807), it must be stressed that Jablonsky was the author of the first volumes of that work (Bousquet 2016), i.e. the first volume on butterflies and the first volume on beetles. Herbst took over the job after the untimely death of Jablonsky in 1787, at the age of 31. The ten volumes on beetles edited by Herbst and Jablonsky from 1785 to 1806 were probably the most important coleopterological work of the time. It is important to notice that also in the plate by Jablonsky the specimen of *Megasoma gyas* displays a short and wide cephalic horn. This particular shape of cephalic horn matches exactly with both the xylography and the painted picture in Marcgraf's publications. This is a key point because in 1789 the French scientist Guillaume Antoine Olivier (1756-1814) described *Scarabaeus typhon* and *Scarabaeus laniger*. The specimens of Olivier show two different features of the cephalic horn: long, thick, with a bifurcated apex the former, as shown in plate XVI at n. 252 (Fig. 5) and shorter, flatter and wider, with a very-well bifurcated apex the latter, as shown in plate XXVIII at n. 247 (Fig. 6). Olivier named his first specimen *Scarabaeus typhon*. Olivier's *Scarabaeus typhon* actually represents the species that up to now has been indicated as *Megasoma gyas* s.l. by the majority of authors. Burmeister (1847) established the synonymy between *Megalosoma* (a junior synonym of *Megasoma* Kirby) *Typhon* Olivier and *Scarabaeus Gygas* Jablonsky, giving priority to *typhon.* Later, in his *Catalogus Coleopterorum* of 1868, Harold mantained the synonymy of Burmeister, but in his addendum of 1871 changed his mind and established the priority of the name *gyas* over *typhon* saying (pag. 121-122): "...Der Jablonsky'sche Name *gyas* von 1785 hat die Prioritat; warum ihn Burmeister, der irrthumlich *Gygas* schreibt, zuruckgesetzt hat, ist ganz unerklarlich...". Also *Scarabaeus laniger* was incorrectly synonymized with *M. typhon*, always by Burmeister in 1847, considered as a "variation B" of the former, variation with a short horn (*Cornu capitis brevius, in apice late furcatum, in ipsa basi tuberculatum*). Before him, Kirby and Spence (1826) still had cited *Typhon* and *lanigerum* as separate species. But taking into account Harold's subsequent actions, the final result is that now *laniger* is correctly synonymyzed with the true *Megasoma gyas*, with which it shares the same specific characters. As regards the types of *S. typhon* and *S. laniger* , they are the specimens illustrated in the above mentioned plates of Olivier's *Entomologie ou histoire naturelle des insectes* (1789). For preparing his book Olivier travelled through England and Holland, with the goal to visit the private cabinets of collectors and to draw the species which were not available in Paris. It is likely that the specimens of *S. typhon* and *S. laniger* illustrated by Olivier were kept in collections he visited during the aforementioned trips. Olivier's reference under the description of *Scarabaeus laniger* "du cabinet de Mr. Juliaans" (from the collection of Mr. Juliaans) clearly indicates a Dutch family name. As for *Scarabaeus typhon*, the description of Olivier reported no localities, apart from the indication "du Musée Britannique" (from the British Museum). A recent search by Kazuho Kobayashi at the Natural History Museum in

London revealed the presence of old specimens (ex Fry collection, dated around 1900) of classical "long-horned *gyas*" coming from Rio de Janeiro and Bahia.

In his description Jablonsky listed three references. The first reference is "*Fuessly* Mag. I. p.37": in his work of 1778, Johan Caspar Fuesly reported a description of Voets' "Kaferwerk", *Scarabaeus Goliath.* The Latin description is the same reported by Jablonsky; there is also a numeric reference, Tab. XVII fig. 114. The third reference is "*Goeze* Ent, Beytr. I. p.56. n.11.": In his work of 1777 Johan August Ephraim Goeze also referred to "*Voet.* Scar. Terric. p. 27. No.114. t.17. f.114, giving a short latin description and the title "*Goliath,* der *americaniche gelbe Bar*". It is clearly the same insect of Fuesly. The second reference given by Jablonsky it is directly to "*Scarabaeus Goliath. Voet.* Scar, tab. 17. fig. 114".

Johann Euseb Voets (1706-1778) was a Dutch physician, poet and entomologist. Voets died before the publication of Jablonsky, hence it is obvious that his description of *Scarabaeus Goliath* must be precedent, also because Fuesly in 1778 and Goeze in 1777 referred to him. His oldest work *Catalogue raisonné ou systématique du genre des insectes, qu'on appelle Coleoptrées*, was apparently published in livraisons starting in 1766. The first part was noticed, without further qualification, in April 1767 by the *Gazette littéraire de l'Europe.* Other parts were issued in 1776 and 1781. Finally, the work was completed and published by Bakhuysen in 1806, under the name C*atalogus systematicus Coleopterorum.* (Bousquet 2016). In the work of 1785, published after his death, "Beschreibungen and Abbildungen…" magistrally illustrated by G.W.F. Panzer, the already cited fig. 114 in plate XVII (our Fig. 3) shows perfect coincidence with Jablonsky's image. In fact Jablonsky wrote:…*I tried to find a previous right description, but without result. Only Voet could see the insect in the collection of dr. Luchmann, and from there he represented it….*For that Jablonsky used the image of Voet but *...giving a more appropriate description, because I trust more on Voets' images than in his descriptions...and because Voets used a name "Goliath" which was already used by Linnaeus for another beetle without horn, I decided to use a name of a Titan, Gyas…*

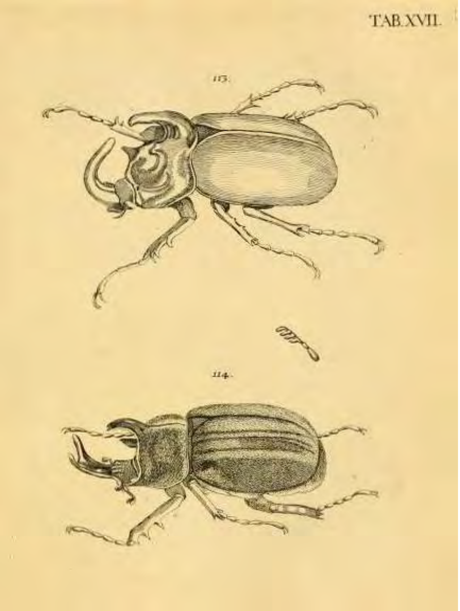

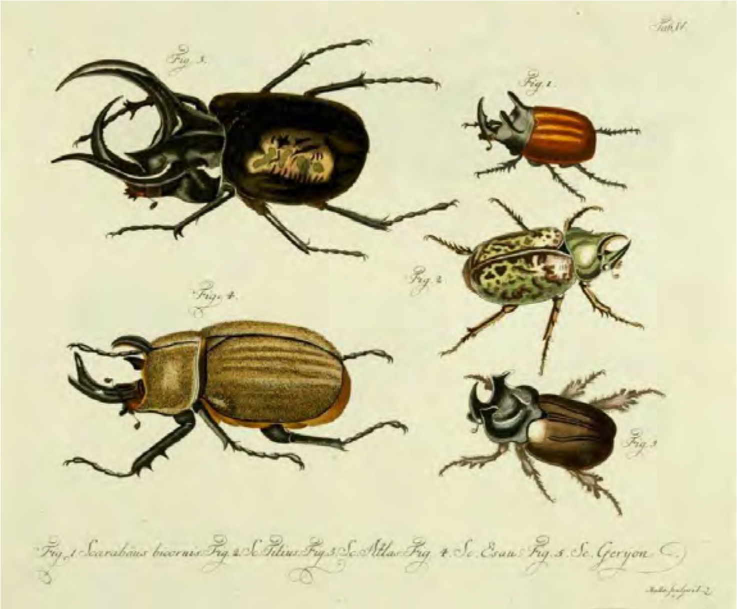

Fig. 3 **Fig. 4**

Fig. 3. Johann Euseb Voets, 1782 edition of "Kaferwerk", fig. 114. *Scarabaeus Goliath*

Fig. 4. Carl Gustav Jablonsky 1785. "Natursystem aller bekannten…." Vol. I. Kafer, fig. 4. *Scarabaeus gyas.* In this plate the insect curiously appeared with the name of *Scarabaeus esau.* For this reason *esau* is considered as synonymous of *gyas*. Some plates appeared with the name of *gyas*, some other appeared with the name of *esau*. But no description of *esau* was provided.

Therefore Voets' name *Scarabaeus Goliath* should have priority over *gyas*, however, Voets' *Catalogus Systematicus Coleopterorum*, fails to fulfill the requirements in the ICZN (Article 11.4) that for scientific names to be available, the entirety of the work in which they appear must be consistently binomial. Voets' names varied from 2 to 5 names in series, thus violating this rule, so none of Voets' names, even those which happened to be binomial, are available for use in modern scientific literature (see Alonso-Zarazaga and Lyal (1999:8), following Sherborn (1902:liv), both citations in Bousquet 2016; see also interpretation of Krell (2012) on nomenclature and synonymy of *Trichius*). The original Luchmann's specimen is apparently lost. The iconography by Marcgraf and Voets/Jablonsky allows us to state that **the first "*gyas*" described with the Linnaean scientific method had a short, flat and wide cephalic horn** (Figs 3-4). Moreover, these evidences match the ancient description of Marcgraf, but we were then in the pre-Linnaean age, so the name was only a generic "Taurus volans". In this case, since Jablonsky's type doesn't match with Olivier's *typhon*, necessary changes in taxonomy need to be made. As for the type locality, then the travels to the "West Indies" meant also the coast near the Antilles, e.g. the Guyanas, Venezuela, and obviously, as we saw before, the Northern Coast of Brazil.

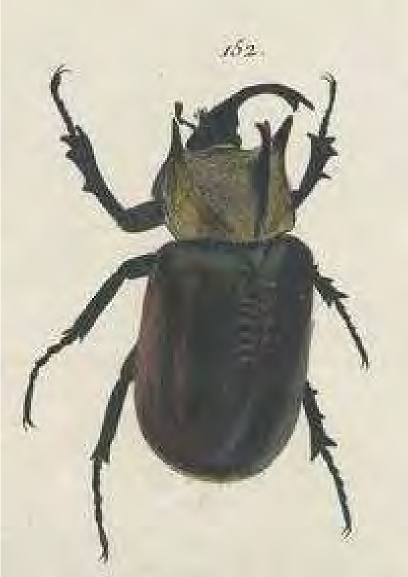

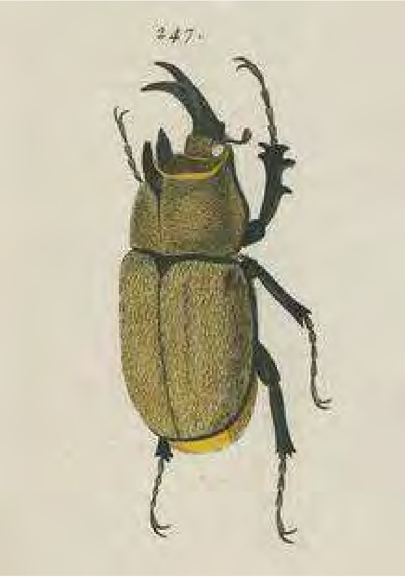

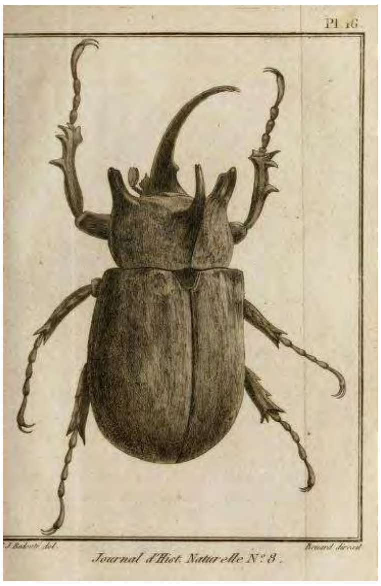

Fig. 5 **Fig. 6** **Fig. 7**

Fig. 5. G-A Olivier 1789. "Entomologie ou Histoire Naturelle des Insectes" no. 252. *Scarabaeus typhon*

Fig. 6. G-A Olivier 1789. "Entomologie ou Histoire Naturelle des Insectes" no. 247. *Scarabaeus laniger*

Fig. 7. G-A Olivier 1792. Journal d'Histoire Naturelle. no. 8. *Scarabaeus entellus*

In 1968 H. Fischer described *M. rumbucheri* (afterwards considered a subspecies of *M. gyas:* see Endrodi 1971) from Rio Pajeú, Planalto da Borborema, Pernambuco, Brazil. But this taxon actually displays the same characters of Marcgraf and Voets/Jablonsky original descriptions and is therefore a junior synonym of *M. gyas* (Fig. 8). Curiously in 1991 Kurt Rumbucher, to whom the Fischer's subspecies had been dedicated, while reviewing the variability of *M. gyas* challenged Fischer's claims, suggesting that the taxon *rumbucheri* fell within the variability of *M. gyas* s.l.. This opinion was supported by photos of *M. gyas* s.l. specimens in different sizes and tables with measurements. He did not examine the aedeagus and did not assess the geographical variability of the specimens he had examined.

In 2003 S. Nagai described *M. gyas porioni* from Jaguaquara, Bahia state, Brazil, dedicated to

the French entomologist Thierry Porion. The main character currently in use in order to distinguishing this subspecies is a long, normally straight, cephalic horn (despite the fact that in the original description is indicated as short and thicker in middle area), showing in the majority of cases a medium depressed zone, with a normally bifurcate apex (Fig. 15). This is a character we find in the typus of *S. typhon* Olivier. To conclude: no great external differences between the "long-horned *gyas*", *M. gyas porioni* and Olivier's *S. typhon* are found (Figs 5-7). The synonymy between *M. gyas porioni* and the "long-horned" *gyas* was also suggested by Grossi et al. (2008).

Grossi, Vaz-de-Mello and Coelho Grossi (2008) hypothesized the presence of *M. gyas* in the state of Santa Catarina and the same year Leonello Milani described *M. gyas prandii*, from Santa Catarina State. This is the southernmost distributional record for *M. gyas*. Unlike the aforementioned subspecies, in this case both the geographical isolation and the peculiar morphology of the taxon leave little doubt about its validity as a subspecies, although, due to the present new arrangement, it must be considered as a subspecies of *Megasoma typhon*. None of the specimens of *M. typhon prandii* we have examined thus far displays a depressed area in the middle of the cephalic horn. All examined specimens show a thick long horn, often curved backwards, without any flattened area, and they have a distinctly bifurcate apex bent upwards. Besides the type locality, it was recently possible to find (author's unpublished data) other specimens of *M. typhon prandii* in old collections, from even more southern localities, labelled "Porto Alegre" (Rio Grande do Sul state in Brazil) or "surroundings of Porto Alegre" (around 1930, collection Ugo Bosia, Asti, Italy). Interestingly all those old specimens have an old label "*M. typhon*".

Revision of the species of *Megasoma* (*Megasoma) gyas* species-group
The *Megasoma gyas* species group, based on the present revision, consists of three species, one of them polytypic, with an overall distribution occupying most of Eastern Brazil, extending northwards up to Ceará (estimated latitude 3°42'02"N) and southwards to Rio Grande Do Sul (estimated latitude 30°00'44"S) states.

***Megasoma* (*Megasoma*) *gyas* (Jablonsky *in* Herbst, 1785)**
ref.: *Enéma, Enena Brasiliensibus, Escaravellha Lusitanis, Taurus volans*, Marcgraf 1648
ref.: *Scarabaeus Goliath*, Voets 1766
syn.: *Scarabaeus esau*, Jablonsky 1785
syn.: *Scarabaeus laniger*, Olivier 1789
syn.: *Scarabaeus monoceros*, Weber 1801
syn.: *Megasoma gyas rumbucheri*, Fischer 1968 **New synonymy**

Distribution. *Megasoma gyas* occurs in the Caatinga biome of the Brazilian States of Piauí, Ceará, Rio Grande do Norte, Paraíba, Pernambuco, Alagoas, Sergipe and Bahia (Fig. 9). The distribution range of this species overlaps a portion of the "subregioes nordestinas" of "Meio-norte" and "Sertao" regions. (Fig. 10). The Caatinga biome (xeric shrubland and thorn forest) occupies an area of 497 thousands sq. miles, i.e. 10% of Brazilian territory. It's a recent biome located on an ancient seabed. This biome experiences long periods of drought, which can last up to 8 months. It is mainly composed by dry-Savannah (Brasilia's Botanical Garden; Coutinho 2016). **Material examined.** The studied material was provided by the CERPE, the Collection of the Universidade Fereral Rural de Pernambuco-UFRPE, with recent material from the States of Piauí, Ceará, Rio Grande do Norte, Pernambuco, Alagoas and Sergipe; the Universidade Federal do Mato Grosso-CEMT and from private Collections (EPGC, MPC). In order to re-describe the taxon, we have chosen a male from the original series of Kurt Rumbucher (herein shown with the original label). The list of examined material enumerates 19 specimens as follows: 9 major ♂ labeled: Alagoas, Canapi – X 2018, J. Dantas Leg.; PE Timbanba 1und1/2 St. (auto) Landeinwarts von Recife ♂ Meg. rumbucheri Dezember 79; UFSE – Sergipe; UFSE – Sergipe; Areia PB Brasil VI 1945; Brasil, Pernambuco, Custódia X 2012 leg. C.E.B Nobre; Ceará - no data; Ceará - no data; Brasil, Pernambuco, São José do Egito (sitio Humaitá) coleta manual 13 IV 2010 R.M. Correia (col) UFMT; 4 minor ♂ labeled: Coleção didatica UFPI – Teresina PI São Francisco 24 IV 2001; Brasil, Sergipe, Capela – Ref. Vida Silv. - Mata do Jumco (RUSMJ) – Sede – 25 III 2014 Leg. O.G. Moura; Brasil, Rio Grande do Norte. Tenente Laurentino Cruz. I 2015 – 6.167S/6.167W – 740m. Leg. R. Andreazze; Coleção didatica UFPB – Patos; 6♀ labeled: Alagoas, Canapí – X 2018 J. Dantas leg.; Brasil, Rio Grande do Norte. Tenente Laurentino Cruz. I 2015 – 6.167S/6.167W; PE – Igarassu – Três Ladeiras – Usina São José XII 2017 Kevin Coll.; Coleção didatica UFPB – Patos; Ceará - no data; PE – Timbanba Dez.79.

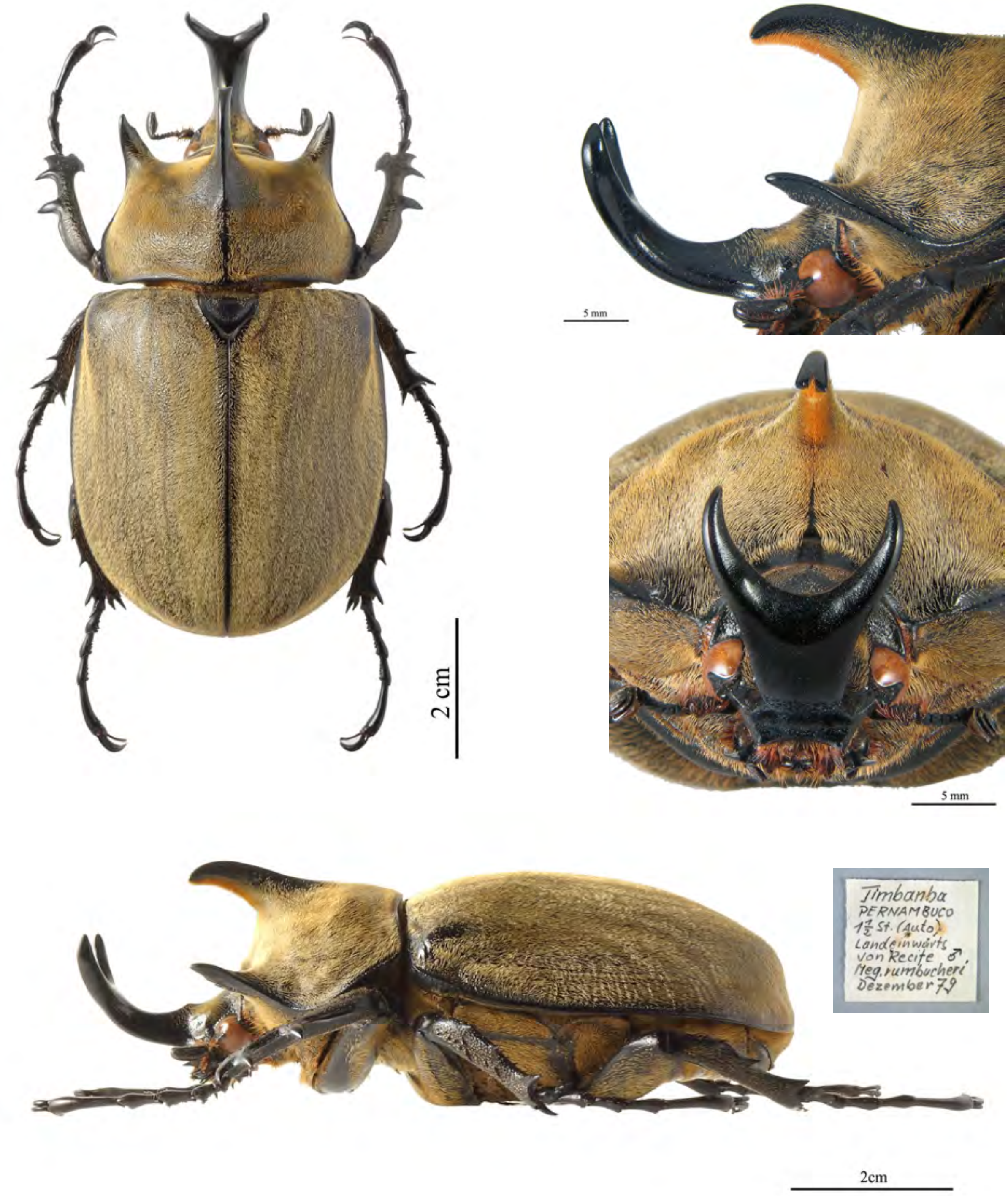

Fig. 8. Dorsal and lateral view with detail of cephalic horn of *Megasoma gyas* from Brazil, Pernambuco. ♂ 90 mm.

Male redescription. (Fig. 8)

Dimensions. L: mm. 77; TL: mm. 88; PL: mm. 21, PW: mm. 35; EL: mm. 49, EW: mm. 42, CL: mm. 23; PH: mm. 8.5. **Color.** Uniformly dark ebony brown covered by a yellowish short, fine, thick pubescence; head, including horn, consistently black with the basal part near pronotum with yellowish sparse bristles. **Head.** Cephalic horn**:** short, projecting forwards and curved upwards. In lateral view flat, distally bent upwards. In dorsal view, narrower at the

base, gradually broadened towards the distinctly forked apex. Apex U-shaped, with slightly divergent, long, tips (Fig. 8). Distance between tips 11.5 mm. Sides bordered with a weak rim hardly detectable, from base to mid-length. Dorsal side at the base with the relief of an almost imperceptible tooth, in this that is a major male; tooth totally absent in medium and minor males. **Clypeus**. Anterior edge concave, lateral angles with a small tooth, projecting forward, surface punctate. **Mandibles**. Each one with two small lateral teeth. **Pronotum**. The whole surface covered by a fine, dense, plentiful yellowish pubescence. Anterior angles projecting as small but elongate, sharp, parallel horns, slightly bent outwards; width at base 4.5 mm; length from base 8.5 mm; distance between apices of anterior horns 23 mm. Medial thoracic horn longer then laterals, length 15 mm., with a characteristic sickle-shaped form, dorsal side with a glossy black line, ventral side of medial horn with recumbent fine pubescence. PL/TH ratio 2.470. L/PL ratio 3.666, showing a fairly elongated feature of the body. **Scutellum.** Form triangular, each side with 8.1 mm, surface glabrous, finely punctate, apex smooth. **Elytra.** Surface covered by fine, dense, recumbent yellowish pubescence apart elytral suture and epipleure glabrous; EL/EW ratio 1.166. Elytral surface covered by variable number (two or three on each elytron) visible longitudinal ridges: sutural edge black, glabrous, punctate; the others pubescent ridges spaced out. Elytra in lateral view more convex proximally and then gradually flattened towards apex. L/EL ratio 1.571, elongate.

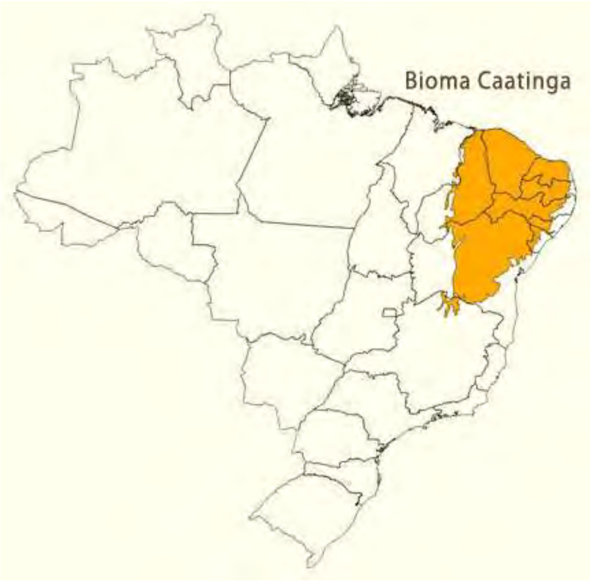

Fig. 9

Fig. 9. Surface of bioma Caatinga in Brazil. (www.brasilescola.uol.com.br)

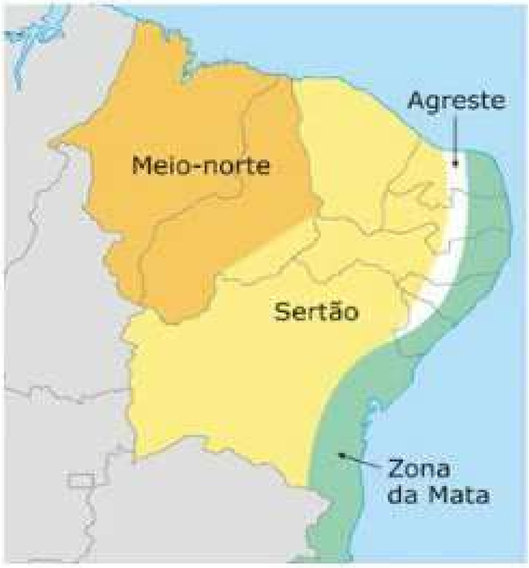

Fig. 10

Fig. 10. The subregions in NE Brazil. (www.brasilescola.uol.com.br)

Pygidium. Strongly convex, with very fine, dense punctuation, hidden by thin, short, greyish-brown pubescence. **Abdomen**. Sides covered with short, very fine, yellowish-brown pubescence, medially almost glabrous. **Legs.** Fore tibia almost straight, inner apical edge strongly dilated inwards, 23 mm. in length. Anterior edge of protibia V-shaped. Lateral edge with three strong teeth, decreasing in size proximally, from the basal to the apical; basal tooth more distant from subapical tooth than the latter from apical tooth. Basal and subapical teeth large, triangular, thick, sharp, pointing rearwards; apical tooth short, pointing forwards. Inner apical spur strongly downcurved, as long as apical tooth. Fore tarsi length 25 mm. **Aedeagus.** Parameres elongate and narrow, as in Tab. 2A-B. **Variation, males.** Major and medium males always with the apex of cephalic horn U-shaped, with long tips. (Fig. 8). Body fairly elongate

(see ratios). Minor males, with cephalic horn's lenghth not more then 2-times the head's length, from vertex to clypeus, show the apex of cephalic horn V-shaped, with shorter tips and a rounder feature of the body. **Measurements of males.** The variability of examined specimens ranges as follows in mm. L: 53-77; TL: 59-88; PL: 14-21; PW: 24-35; EL: 38-49; EW: 30-43; CL: 10-23; FL: 15-23; TF: 17-25.

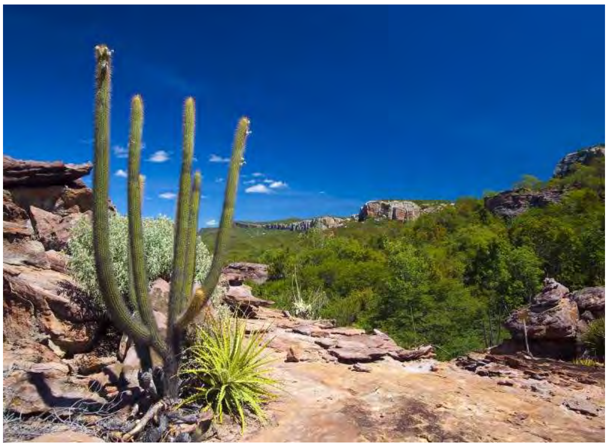

Fig. 11

Fig. 11. View of Caatinga Brasileira biome. (www.nossaciencia.com.br)

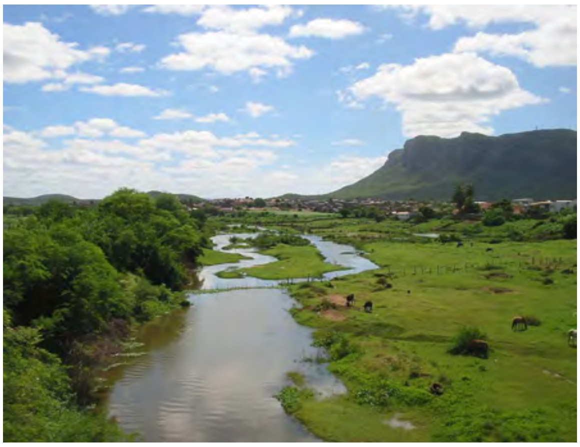

Fig. 12

Fig. 12. View of Rio Pajeú, Serra Talhada, Pernambuco.
(www.clickeaprenda.uol.com.br)

Female description. (Fig. 13).

Dimensions. L: mm.54; PL: mm.16; PW: mm.23; EL: mm.34; EW: mm.30. **Color.** Uniformly black; elytra with 6/7 of its surface covered by grey-brownish dense pilosity. **Head**. Fronto-clypeal suture with a double conical tubercle. **Clypeus.** Surface finely punctate; lateral angles teeth like directed forwards and upwards; distance between apex mm.2; apical edge between angles concave. **Pronotum**. Surface dull, coarsely punctate-rugose, strongly convex; posterior medial carina mm. 8 long, ½ of total PL. Anterior angles obtusely projecting, yet with sharp tips. Lateral edges with presence of sparse bristles. **Scutellum**. Triangular, smooth, shiny, impunctate. **Elytra.** Surface rugo punctate at anterior region, glossy black; punctate black surface extending for 8 mm. in length, almost 1/7 of L. Elytral pubescence thick, uniform, with clearly visible longitudinal ridges, three or more for each elytron, almost equidistant. Dorsal longitudinal and lateral borders glossy black, with very fine punctuation. **Pygidium.** In lateral view, concave, with very fine punctuation. Surface in basal half covered with short, fine, reduced greyish pubescence; in apical half with scattered, erected brown-reddish setae. **Abdomen.** Sternites finely punctate, covered by short, yellowish-brown pilosity, except for a small central portion in the middle of sternites III-IV-V. **Legs**. Fore tibiae shorter than in males, TL mm.15, and shorter than tarsi, TF mm.17; external sides with three strong teeth almost equal in length, with the subapical tooth a little longer. Lateral teeth and inner apical spur smaller than in males. **Measurements of females.** The variability of examined specimens ranges as follows in mm. L: 52-60; PL: 15-16; PW: 23-27; EL: 34-39; EW: 30-34; FL: 15-16; TF: 17-18; HL: 6-8.

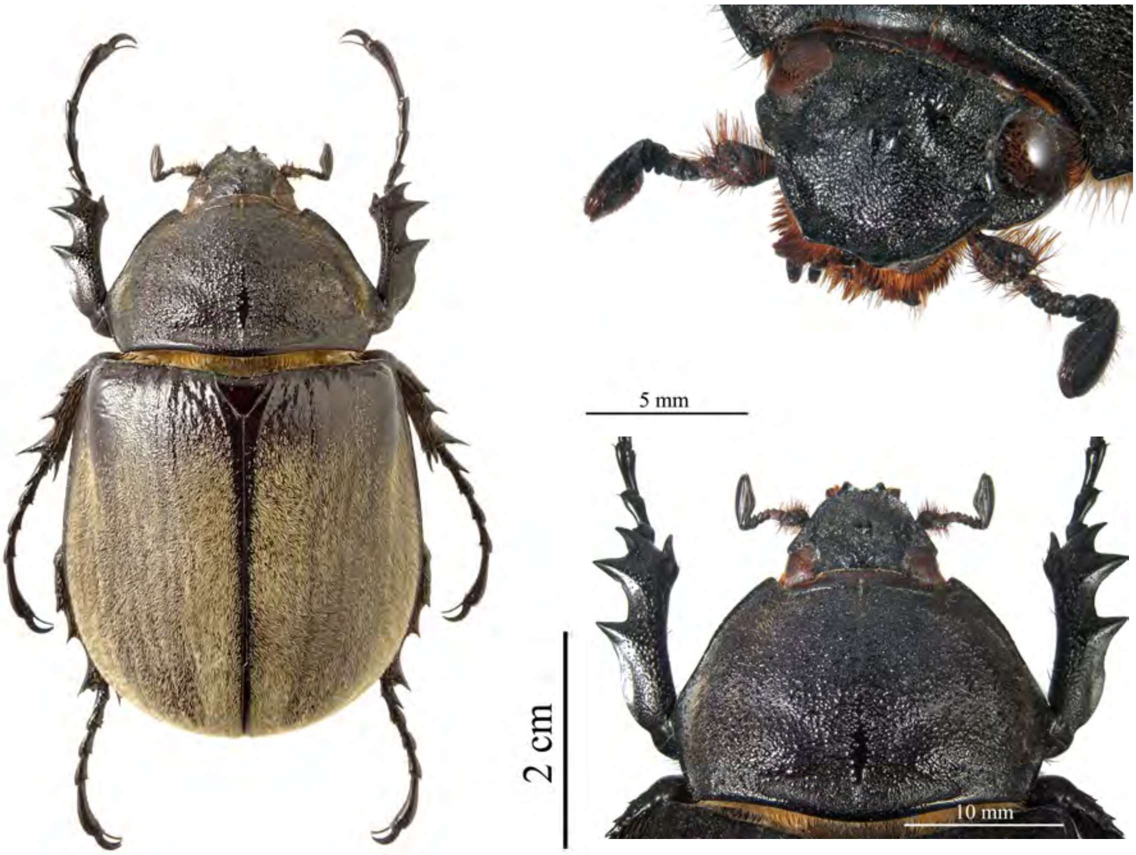

Fig. 13. Dorsal view with detail of carina and double head's tubercle of *Megasoma gyas* ♀ from Brazil, Rio Grande do Norte, Tenente Laurentino.

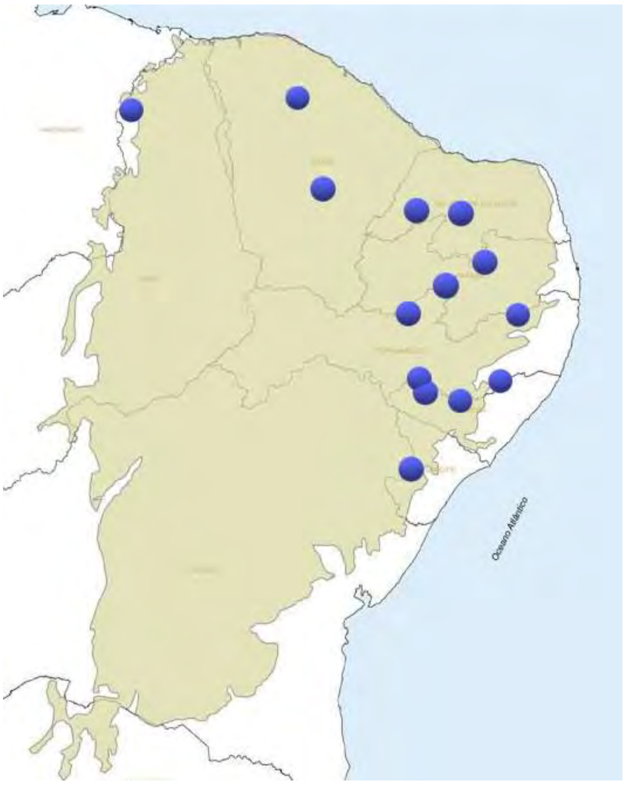

Fig. 14. Map of Caatinga's biome with frequency records (grouped) of *Megasoma gyas* in Northeastern Brazil.

***Megasoma* (*Megasoma*) *typhon ssp. typhon* (Olivier, 1789) stat. nov.**
syn.: *Megasoma gyas* ssp. *porioni,* Nagai 2003. **New synonymy**

Type material: the **Holotype**, i.e. the specimen illustrated by Olivier (Fig. 5) is probably lost (Kobayashi, after research at BMNH, personal communication, 2019). The designation of a neotype does not seem necessary since the species is well characterized and a search for the type in historical collections is still ongoing. The examined material, all kept in Universities and private collections, comes from several localities in Bahia, Minas Gerais, Rio de Janeiro and São Paulo states.
Distribution. As explaned above, the classical "long-horned" beetle up to now called *M. gyas gyas*, is actually a distinct species which needs to be named *M. typhon* (Olivier, 1789). It occurs through the Mata Atlantica biome along the coastal areas of the Brazialian states of Bahia, Espirito Santo, Rio de Janeiro, São Paulo and Minas Gerais. The biome Mata Atlântica (Atlantic Rain Forest) occupies an area equivalent to 622.000 sq. miles, i.e. 13% of Brazilian territory and consists mainly of forests that run along the coastline from the State of Rio Grande do Norte to the State of Rio Grande do Sul. Due to its high human population density it is one of the most deforested areas of Brazil. Only 7% of its original vegetation remains, scattered over hundreds of mostly small fragments. The Mata Atlântica presents a diversified group of forest ecosystems and a variety of floristic structures connected to specific different climatic conditions, all them enjoying the humid winds that blow from the ocean (Brasilia's Bothanical Garden; Coutinho 2016). We have no records of *M. typhon* from Paraná state, being São Paulo state the southernmost record. This species shows an interesting variability in the shape of cephalic and thoracic horns, mainly in the flat or thin section of the former and in the tips of the latter. This variability however is found all over the distribution range of the species and therefore is an individual variability without a geographical meaning. Based on this new interpretation, *M. gyas porioni* Nagai is a synonym of *M. typhon typhon*.
Material examined. The studied material is housed at CEMT, BMNH, INPA, MSNM, EUMJ, in EPCG and MPC collections and in many other private collections. The description below is based on a specimen from Bahia state, Jaguaquara locality, which closely resembles the specimen illustrated by Olivier. Other specimens from different localities are shown in order to illustrate the morphological variability uniformly found all over the distributional range of the species. The list of examined material enumerates more than 100 specimens (major ♂ 80%, minor ♂ 10%, ♀ 10%) coming from the following Brazilian localities: Jaguaquara, Amargosa, Salobrinho, Arataca, Ilhéus, Porto Seguro, Olivença, Una, Itamajú, Itabuna, Jequié (Bahia state); Ubatuba and some specimens labeled "province" of SP (São Paulo state); Teresópolis, Rio das Ostras, Guapimirim, Xerém, Nova Iguaçu (Rio de Janeiro state); Linhares (Espirito Santo state); Ipatinga, Vale do Rio Doce, Cataguases (Minas Gerais state).

Male redescription. (Fig. 15)
Dimensions. L: mm. 79; TL: mm. 108; PL: mm. 23, PW: mm. 37; EL: mm. 54, EW: mm. 49; CL: mm. 38; PH: mm. 10.5. **Color.** Uniformly dark ebony brown covered by a yellowish short, fine, regular, pilosity; head, including horn, consistently black except for the basal part near pronotum with sparse bristles. Tips of thoracic horns glossy black. **Head. Cephalic horn:** long, projecting forwards and slightly curved upwards. In dorsal view, wider at the base, declining for a length of 6 mm. and then gradually broadened to a medial flattener zone with a maximal width of 5.5 mm., then declining again for a length of 13 mm., and finally gradually broadened towards the forked apex. Apex always V-shaped, with divergent tips (Fig. 15). This feature occours always in minor, medium and major males, with medial or longer horns. Sometimes the apex's tips of cephalic horn, in dorsal view, are slightly bent

backwards, mostly in medium or small specimens. Distance between tips 8.5 mm.. Sides bordered with a weak rim easily detectable, from base to mid-length. Dorsal side at the base with the relief of a distinct tooth, with a max height of 3.5 mm.

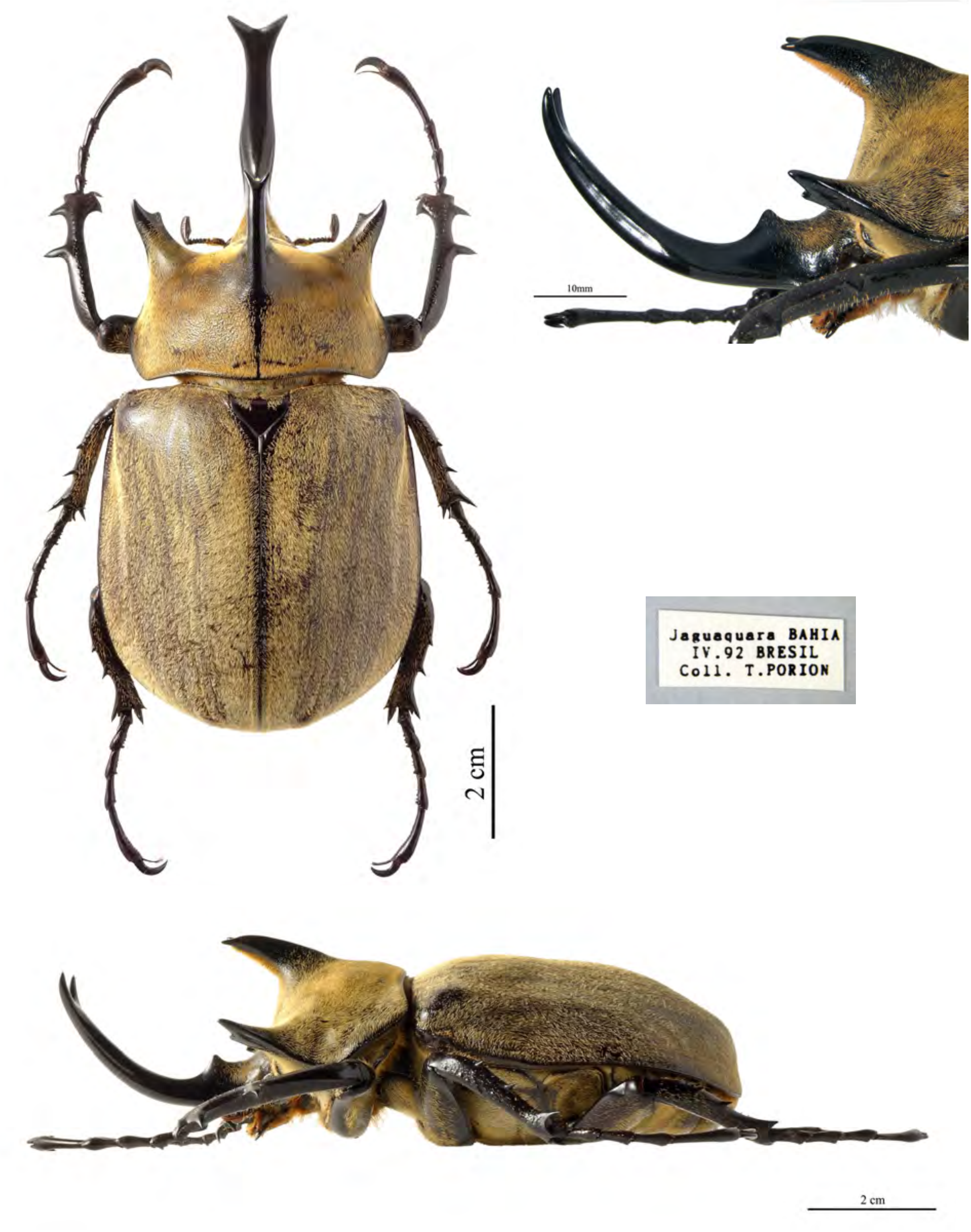

Fig. 15. Dorsal and lateral view of *Megasoma typhon typhon* from Brazil, Bahia, Jaguaquara. ♂ 108 mm.

Clypeus. Anterior edge slightly concave, narrower than thickness of cephalic horn at the base, lateral angles with pointed tooth, projecting forward, surface punctate with presence of sparse bristles. **Mandibles**. Each one with two small lateral teeth. **Pronotum**. The whole surface covered by a regular, fine, dense, yellowish pubescence. Anterior angles projecting as elongate, sharp, divergent horns, distinctly bent outwards, width at base about 9.5 mm., length from base 10.5 mm., distance between apices of anterior horns 37 mm. Medial thoracic horn longer then laterals, length 17 mm., straight, dorsal side with a glossy black line, ventral side of medial horn with plentiful fine pubescence. PL/TH ratio 2.190. **Scutellum.** Big, triangular, 8 mm. long, 9 mm. wide, impunctate, glossy, with lateral bristles. **Elytra.** Covered by a very fine, dense, regular yellowish pubescence except along sutural edge and lateral borders; EL/EW ratio 1.102. Sutural glossy stripe black, limited by some visible ridges; three ridges, or more, almost equally spaced, on each elytron visible under pubescence. Elytra in lateral view bulging, but gradually flattened towards apex. L/EL ratio 1.462, showing an elongate feature of the body. **Pygidium**. Strongly convex, with very fine, yellowish pubescence. **Abdomen**. Laterally covered with very fine, short, reddish-brown pilosity, medially glabrous only for a little area of sternites. **Legs.** Fore tibia slightly rounded inwards, the inner edge strongly dilated at apex, FL, 26 mm.. The anterior edge of protibia V-shaped. Lateral edge with three strong teeth, decreasing in length from the basal to the apical tooth, but with the basal longer than the subapical; the basal tooth more distant from the subapical tooth than the latter from apical. Basal and subapical teeth large, thick, sharp, triangular, pointing rearwards; apical tooth very reduced, pointing forwards. Inner apical spur strongly curved ventrally, distinctly longer than the apical tooth. TF, 29 mm. **Aedeagus.** Overall appearence of the parameres more massive than in *M. gyas*, subrectangular, not narrow, as showed in Tab. 2C-D. **Variation, males.** As usual, development of cephalic and thoracic horns is allometric, but in medium and small specimens of *M. typhon typhon* with shorter cephalic horn, thoracic horns remain well developed. The tooth on dorsal side of cephalic horn is always present, in major, medium and small specimens. Minor males in lateral view, often show a rounder feature of the body. **Measurements.** The variability of the examined specimens ranges as follows in mm. L: 57-85; TL: 65-119; PL: 17-24; PW: 29-40; EL: 41-57; EW: 35-50; CL: 13-38; FL: 19-26; TF: 22-30.

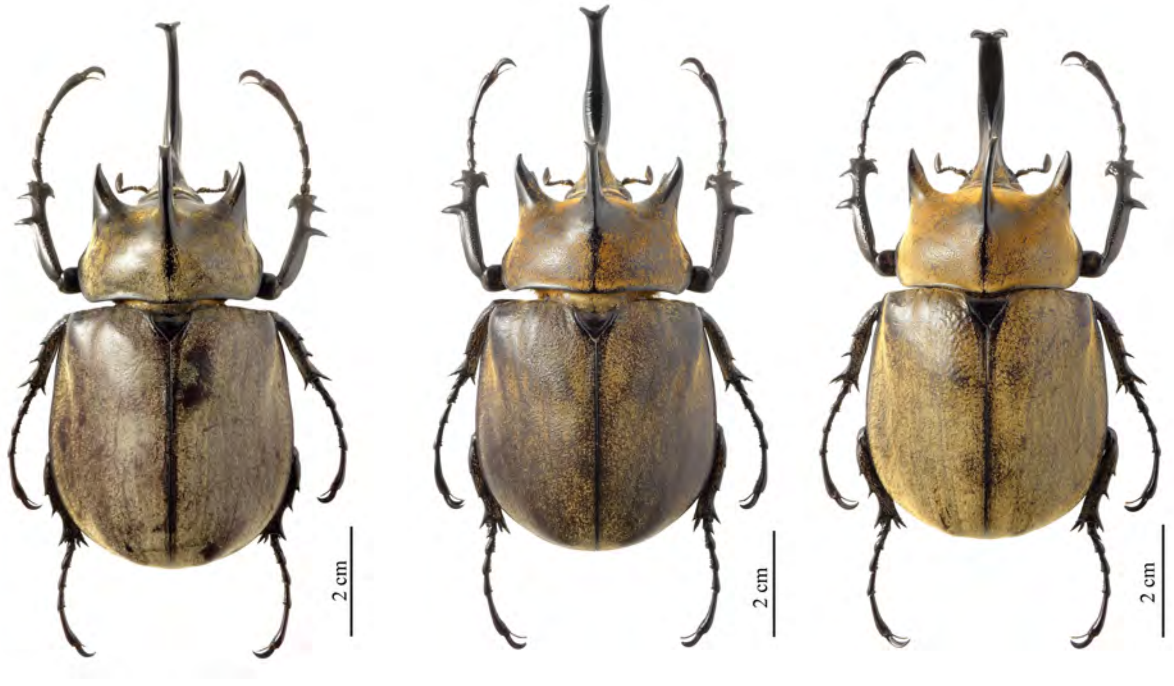

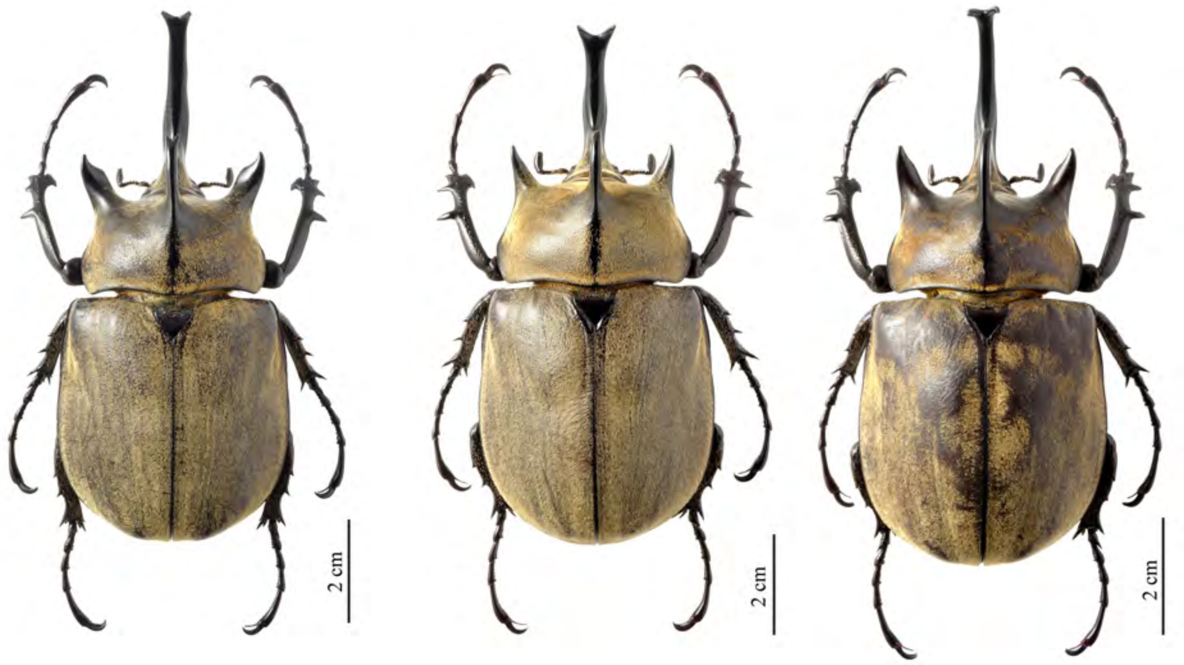

Fig. 16. *Megasoma typhon typhon* gallery from different localities. **A**: Salobrinho, Bahia, Brazil. **B**: São Paulo Province, Brazil. **C**: Arataca, Bahia, Brazil. **D**: Ipatinga, Minas Gerais, Brazil. **E**: Jaguaquara, Bahia, Brazil. **F**: Teresópolis, Rio de Janeiro, Brazil.

Female description. (Fig. 17)

Dimensions. L: 73 mm.; PL: 20 mm., PW: 32 mm.; EL: 49 mm.; EW: 41 mm. **Color.** Uniformly black; elytra covered for the 4/5 of the total surface by a yellow-brownish dense pilosity. **Head**. The middle of fronto-clypeal suture with a single tubercle. **Clypeus.** Finely punctate; anterior lateral angles projecting into a very small tooth directed forwards and a little upwards; distance between apices 3 mm.; apical edge between the angles concave. **Pronotum**. Dull, coarsely punctate-rugose, strongly convex; posterior medial carina 12 mm. long, more than ½ of total length. Anterior angles projecting, obtuse, with a blunt tip. **Scutellum**. Triangular, smooth, shiny, impunctate except for the lower apex. **Elytra.** Punctate-rugose glossy black on a dorsal, longitudinal area, at base extending for 10 mm., almost 1/5 of the EL; the sculpture is steadily fine towards the pubescent surface. The pubescent surface uniformly covered, with clearly visible longitudinal ridges, three or more ridges for each elytron, not equally spaced. Dorsal longitudinal edge of elytron and epipleure glabrous, glossy black, with very fine punctuation. **Pygidium.** In lateral view, profile concave, with very fine punctuation. Surface in basal almost smooth, with reduced greyish pubescence; in apical half with scattered, erected brown-reddish setae. **Abdomen.** Finely punctate, covered by short, brown-yellowish pilosity except for the medial central portion on sternites III-IV-V. **Legs**. Fore tibiae shorter than in the males, shorter than tarsi, 17 mm. long, fairly arcuate, with three lateral strong teeth. The basal and the subapical teeth equal in length; the apical tooth smaller. Inner side with a sligth dilatation at apex. Inner spur curved ventrally almost equal in length as apical tooth. Lateral and inner apical teeth smaller than in the males. TF length mm. 22. **Measurements of females.** The examined material riability ranges in mm. as follows. L: 47-78; PL: 12-21; PW; 20-34; EL; 29-50; EW: 26-44; FL: 11-19; TF: 16-24; HL: 5-11.

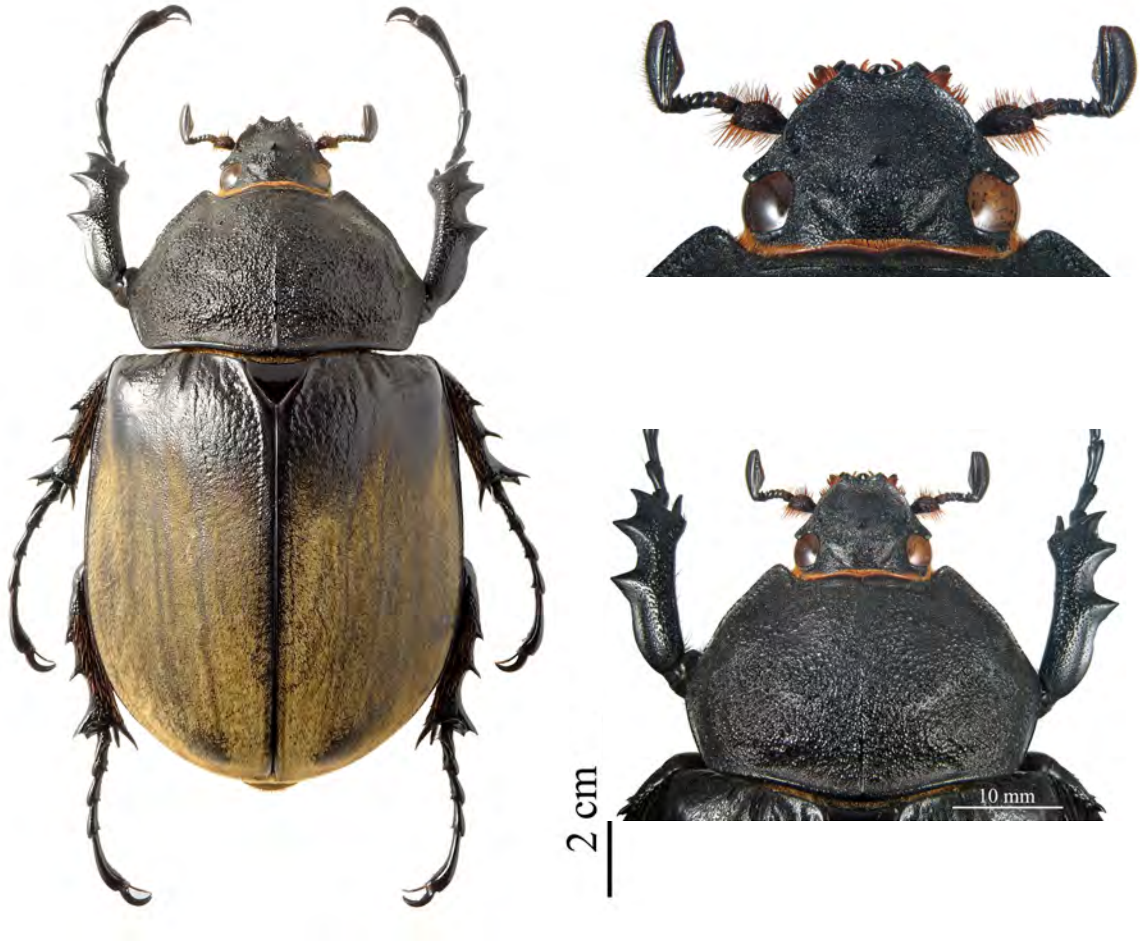

Fig. 17. Dorsal and lateral view with detail of pronotal carina of *M. typhon typhon* ♀ from Ipatinga, Minas Gerais, Brazil

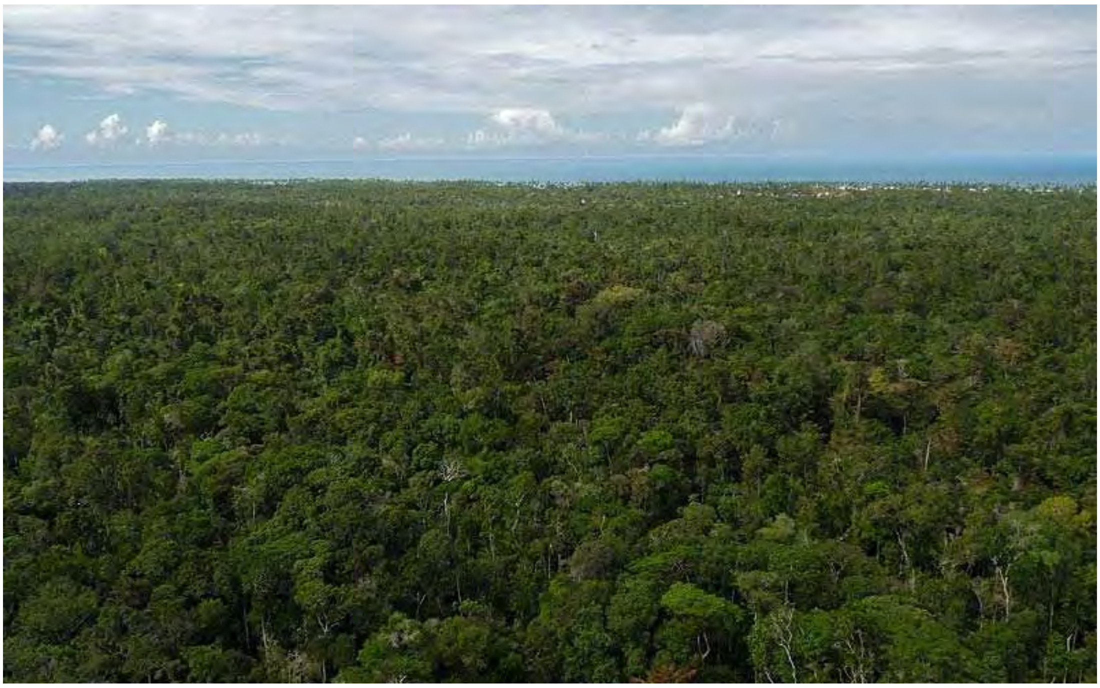

Fig. 18. View of Mata Atlantica near Ilhéus, Bahia, Brazil (www.acordameupovoblog.com)

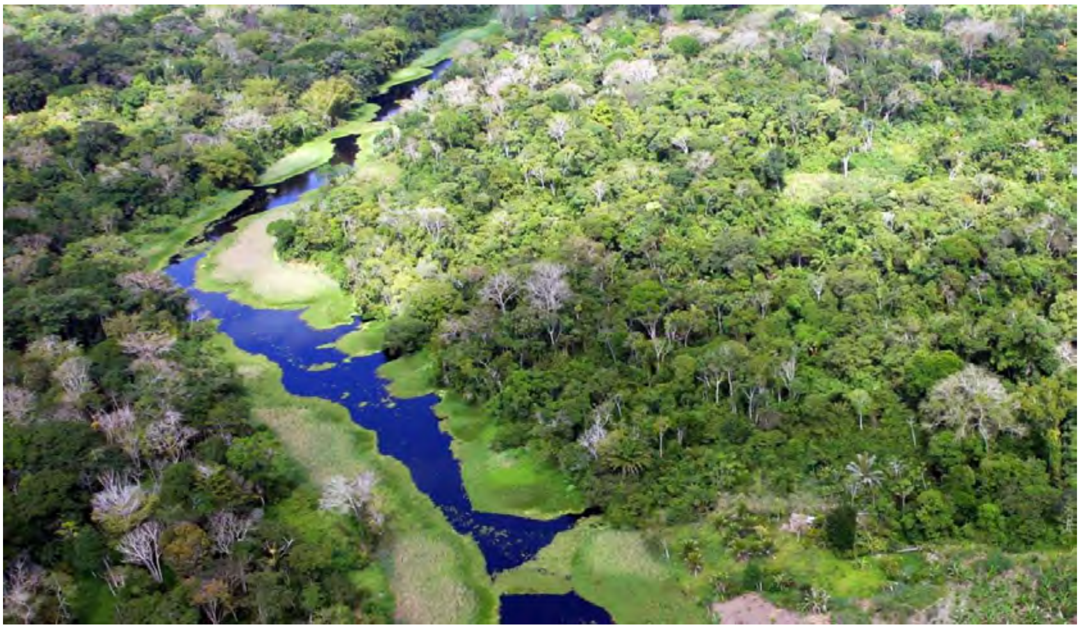

Fig. 19. View of Mata Atlantica near Itabuna, Bahia State, Brazil (www.jornalsportnews.blogspot.com)

***Megasoma* (*Megasoma*) *typhon* ssp. *prandii* Milani 2008 (n. comb.) (Fig. 20).**

Distribution. This is the southernmost subspecies, nowadays restricted to the Serra do Mar region in the northern part of the Santa Catarina state. It displays a constant distinct morphology with respect to *M. typhon typhon*, which in addition to its geographic isolation, allows us to consider this population as a distinct subspecies. **Material examined.** The typical series in MPC collection, the paratype deposited in MSNM, other specimens in private Collections and an interesting old series (dated around 1930) in the Ugo Bosia Collection, Asti, Italy. The following redescription is based on a specimen from Serra do Mar, near Rio dos Cedros, above 180mt asl caught in 2010. After this date very few specimens have been found, suggesting that the subspecies could be threatened by the reduction of its habitat.

Male diagnosis (Fig. 20).
A large *Megasoma* (size: L: 53-78, TL: 62-102; PL: 15-23; PW: 26-36; EL: 33-53; EW: 35-46; CL: 11-40; FL: 17-26; TF: 19-27), uniformly dark brown covered by a yellowish short, fine, regular, pubescence; head, including horn, consistently black. Head. Cephalic horn: long, projecting forwards and noticeably curved upwards. In dorsal view, slightly wider at the base and at the apex, but remaining almost subrectangular laterally, without a medial flattener zone. Apex always V-shaped, with divergent tips (Fig. 20). Pronotum: the whole surface covered by a regular, fine, dense, yellowish pubescence. Anterior angles projecting as elongate, sharp, weakely divergent horns. Medial thoracic horn longer then laterals, straight, dorsal side with a glossy black line. Elytra covered by a very fine, dense, regular yellowish pubescence except along sutural edge and epipleure. Elytra in lateral view not bulging, regularly flattened towards apex. Feature of the body elongate. Legs: Fore tibia slightly rounded inwards, the inner edge strongly dilated at apex. The anterior hedge of protibia V-shaped. Aedeagus as in Fig. 20c. Variation, males: the feature of the apex of cephalic horn is always V-shaped. The presence of a distinct tooth on the dorsal side of cephalic horn is always visible, also in medium and minor ♂. The feature of the body, in lateral view, in medium specimens remains almost flat, only very small specimens show sometimes a rounder body.

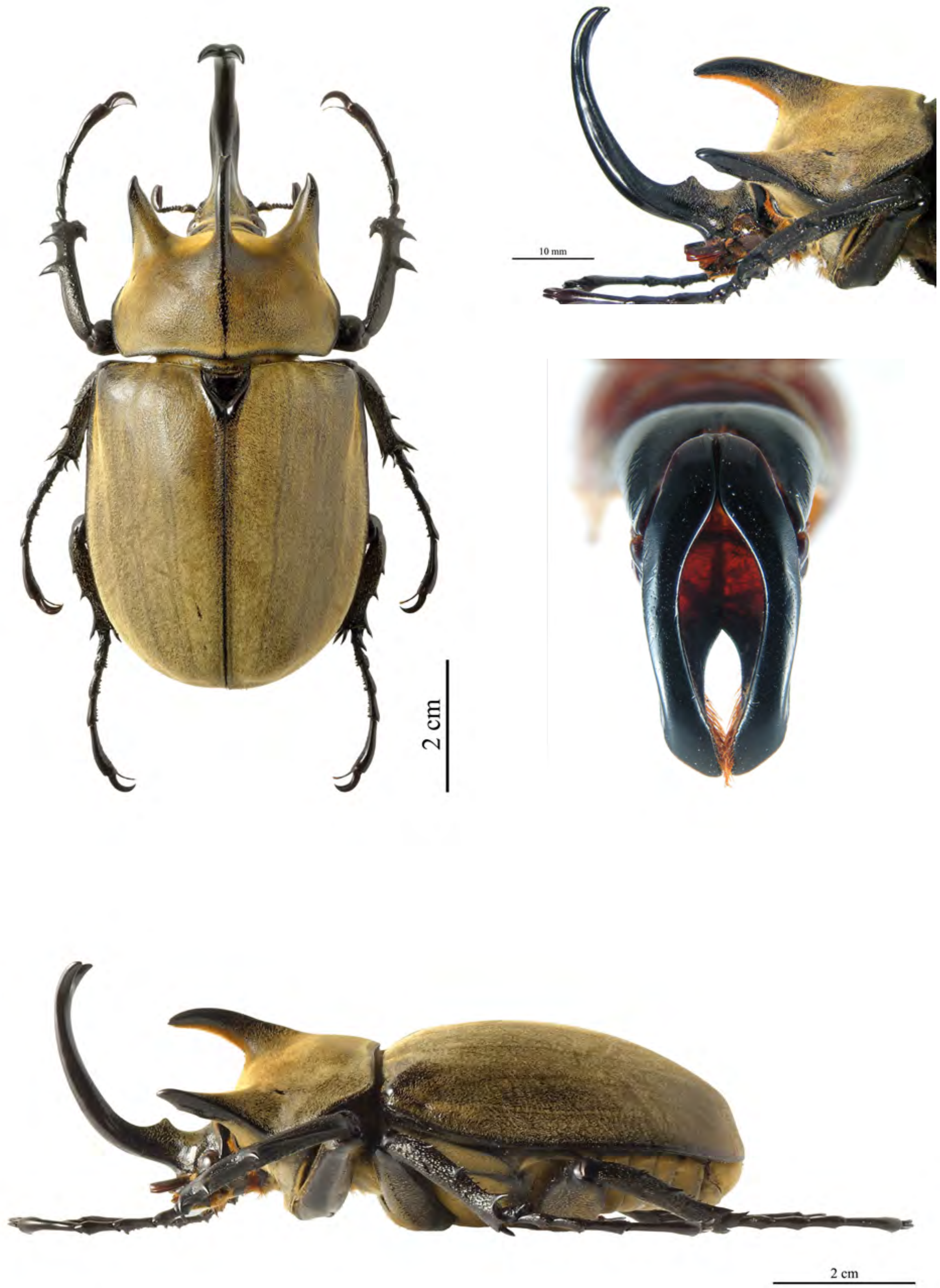

Fig. 20. Dorsal, lateral view and aedeagus of *M. typhon prandii* ♂ from Brazil, Santa Catarina, Serra do Mar.

Female diagnosis (Fig. 21).

A medium-large female of *Megasoma* (size: L: 49-65; PL: 13-18; PW: 21-29; EL: 32-43; EW: 29-36; FL: 13-19; TF: 15-20; HL: 7-9), uniformly black; elytra covered for the 5/6 of total surface by a yellow-brownish dense pilosity. Head: the middle of fronto-clypeal suture with a single tubercle. Clypeus: anterior lateral angles projecting into a very small tooth directed forwards and upwards; in this ssp. the two small teeth are distinctly more acuminate and curved upwards than in other species. Pronotum dull, except for the lateral sides, with sparse bristles. Strongly convex, coarsely punctate-rugose; with posterior medial carina flat, enlarged and smooth. Pronotum more expanded longitudinally and rounded laterally than in other species (Fig. 21). Elytra: punctate-rugose on a dorsal, longitudinal area, glossy black at base; the sculpture is steadily fine towards the pubescent surface. Pubescent surface rather sparse, grossly covered, with not clearly visible longitudinal ridges. Legs: fore tibiae shorter than tarsi, fairly arcuate, with three lateral strong teeth. On mesotibiae and metatibiae the lateral teeth evolving in evident lateral carinae (Fig. 21). These lateral spinous process extending up to 3 millimeters laterally, immediately before of the tarsi's junction.

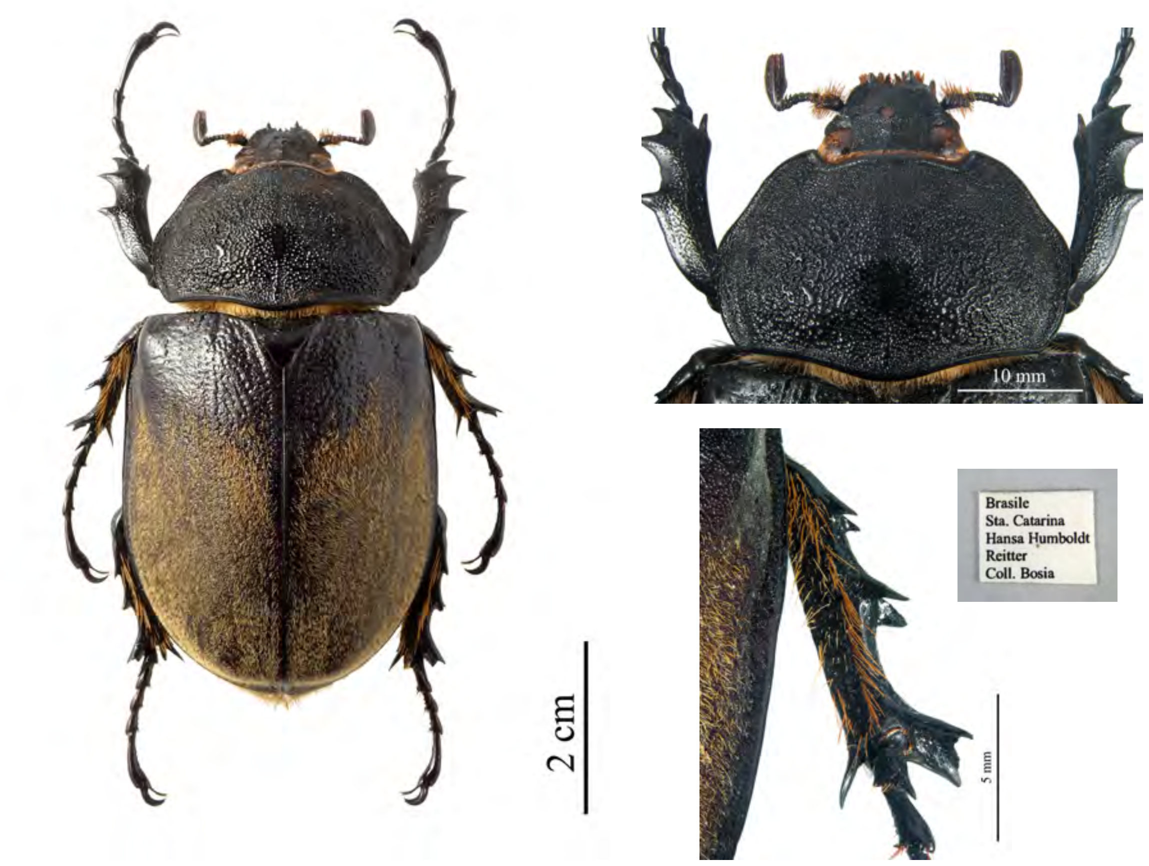

Fig. 21. Dorsal, lateral view with detail of pronotal carina and mesotibia of *M. typhon prandii* ♀ from Brazil, S. Catarina, Hansa Humboldt (now Joinville) with label.

***Megasoma* (*Megasoma*) *hyperion* sp. nov.**

Distribution. The specimens of *Megasoma* from Minas Gerais, São Paulo and Southern Bahia, thus far attributed to *Megasoma gyas rumbucheri*, actually represent a distinct and still unnamed species. Grossi et al. (2008) were the first to remark the disjunct distribution of the

alleged *rumbucheri*, see also Prandi (2016) and Santos et al. (2013). The type locality of the new species falls within the “mata seca ou de cipó” (dry forest) habitat, a crossroad of the three biomes Caatinga, Cerrado and Mata Altlântica. We have also records from Southern Bahia, where the biome gradually turns into Mata Atlântica. In that region this species is sympatic with *M. typhon typhon.* An interesting historical record (1908) has been shown in Be-Kuwa (Kobayashi 2019) from the locality of Paranaíba (Western Minas Gerais state, Brazil). The beetle is quite polymorphic, but the species-specific characters here identified are constant and allow to consider this taxon as a separate species. **Material examined.** The examined material is deposited as detailed in the list of **Paratypes**, divided as follows: 81 major ♂ specimens (>65mm.), 30 minor ♂ and 38 ♀.

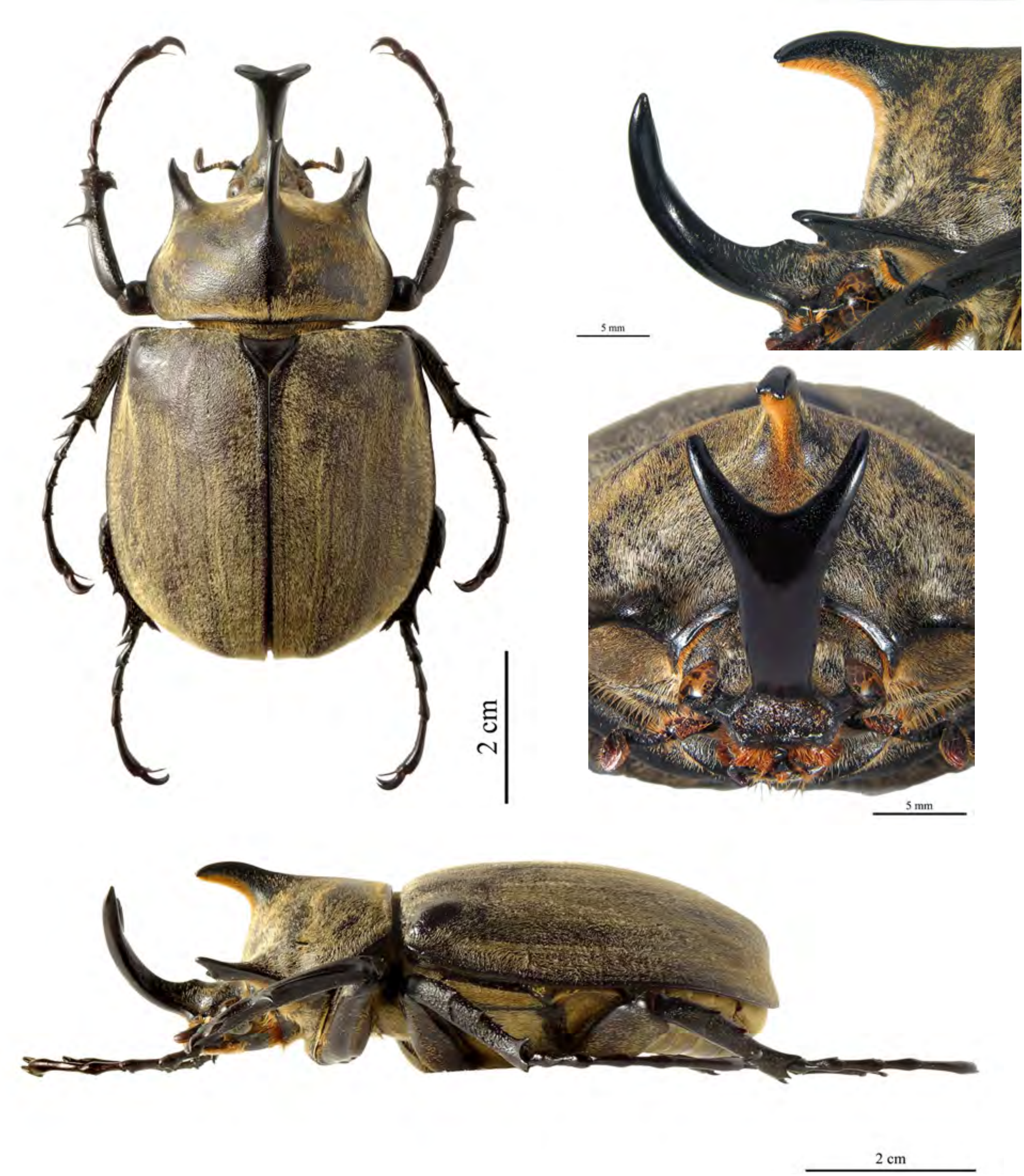

Fig. 22. Dorsal, lateral view with detail of cephalic horn of *Megasoma hyperion* sp.nov. **Holotype** from Brazil, Minas Gerais, Aguas Vermelhas. ♂ mm. 79.

Description of the Male from Brazil, Minas Gerais. (Fig. 22).

Type material: 149 specimens, 1 **Holotype** and 148 **Paratypes**. 1 **Holotype** ♂ from Brazil, Minas Gerais, Aguas Vermelhas, IV 2006 deposited in Universidade Federal Rural de Pernambuco, Recife, Brazil, CERPE. 1 Paratype ♀ from Brazil, same locality, III 2002 (Allotype), deposited in CERPE. 2 ♂, Brazil, Minas Gerais, Salinas, V 2002; same data, 2007; all deposited in CEMT. 1 ♂, Brazil, Minas Gerais, Aguas Vermelhas, V 2006, deposited in MNSM. 1 ♂, Brazil, Minas Gerais, Paracatú, VI 1981; 2 ♂ Brazil, São Paulo, Boituva, VII 1991; 1 ♂ Brazil, MG, Aguas Vermelhas, VI 1992; 1 ♂ Brazil, MG, Jaíba, V 1997; 1 ♂ Brazil, MG, Montes Claros, II 2000; 1 ♀ same locality, IV 2000; 1 ♂ Brazil, MG, Aguas Vermelhas, III 2001; 8 ♂ Brazil, MG, same locality, IV 2001; 1 ♀ same locality, same data; 5 ♂ Brazil, MG, Aguas Vermelhas, III 2002; 1 ♂ Brazil, MG, Capitólio, IV 2004; 1 ♂ 1 ♀ Brazil, MG, Salinas, IV 2005; 35 ♂ Brazil, MG, Aguas Vermelhas, IV 2006; 27 ♀ same locality, V 2006; 18 ♂ same locality, IV 2013; all in Collection E.J. Grossi, Nova Friburgo, RJ, Brazil. 3 ♂ Brazil, MG, Aguas Vermelhas, XII 1994; XII 1994; IV 2014 in Collection K. Kobayashi, Tokyo, Japan. 11 ♂ 1 ♀ Brazil, MG, Aguas vermelhas, IV 2006; 1 ♂ Brazil, MG, Aguas Vermelhas, III 2013; 1 ♂ Brazil, São Paulo, Boituva, VII 1991; 3 ♂ Brazil, MG, Aguas Vermelhas, IV 2013; 5 ♂ Brazil, Aguas Vermelhas, V 2006; 1 ♂ Brazil, MG, Aguas Vermelhas, IV 2005; 1 ♂ 1 ♀ Brazil, MG, Aguas Vermelhas, V 2005; 1 ♀ Brazil, MG, Aguas Vermelhas, V 2008; 2 ♂ Brazil, MG, Aguas Vermelhas, VI 2014; 2 ♂ Brazil, MG, Aguas Vermelhas, III 2016; 7 ♂ Brazil, MG, Aguas Vermelhas, III 2018; all in collection M. Prandi, Salo', Italia.

Measurements of Paratypes. L: mm. 45-71 (mm. 70 in holotype, TL mm. 79); TL: mm. 52-94; PL: mm. 14-24 (mm. 18 in hol.), PW: mm. 22-37 (mm. 32 in hol.); EL: mm. 34-52 (mm. 43 in hol.), EW: mm. 14-47 (mm. 42 in hol.); CL: mm. 5-25 (mm. 22 in hol.); TH: mm. 4-9 (mm. 8 in hol.); FL: mm. 14-25 (mm. 21 in hol.); TF: 18-26 (mm. 24 in hol.). **Color.** Uniformly dark ebony brown covered by a sometimes dense, sometimes sparse rough pilosity; pubescence that turns from the greyish to the yellowish-brown or reddish-brown colour. Head, including horn, consistently black; base of the horn towards pronotum with sparse bristles. Tip of pronotal horns, sutural and lateral edges of elytrae and thorax loustrous black as in legs. **Head. Cephalic horn:** short, projecting forwards and curved upwards. Often in shape of a shovel, including the holotype, sometimes straighter in other paratypes. In dorsal view, narrower at the base, 3 mm., gradually broadened up to a maximum of 7 mm., towards the apex. Apex distinctly forked, always V-shaped, either in small or in large specimens; distance between tips 10 mm. Sides bordered with a weak rim from base to apex, rim detectable on total length. Dorsal side at the base with a small but evident triangular tooth. In lateral view, apex of the tooth blunt, projecting upwards; height of the tooth from base, 1 mm. **Clypeus.** Anterior edge slightly concave, less concave than in *M. gyas*, broader than width of cephalic horn at the base, lateral angles with a small tooth, projecting forward, surface punctate with sparse bristles. **Mandibles** each with two small lateral teeth. **In ventral view**, interocular minimum width (IW) 4 mm., transverse eye diameter (TE) 4.5 mm., IW/TE ratio 1.154. Antennal club, in dorsal view, 3.8 mm. of length. **Pronotum**. Completely covered by rough pubescence. Anterior angles projecting as sharp, elongate, parallel horns, slightly bent outwards; width at base about 4.9 mm., length 8 mm., distance between apices of anterior horns 21 mm. Medial thoracic horn longer then laterals; sickle-shaped in holotype, rather elongate in other specimens, 11 mm. of length. PL/TH ratio 2.250. L/PL ratio 3.888, higher than in *M. gyas.* **Scutellum.** Subtriangular, in the holotype 5 mm. long, 7 mm. wide, largely coarsely punctate, lateral edges and lower apex smooth. **Elytra.** Covered by a rough pubescence, except for a black glossy punctuation around scutellum, along epipleure and longitudinal elytral suture; EL/EW ratio 1.023. Sutural punctate black stripe limited by very fine, visible ridges, well detectables under pubescence; three or more other similar ridges,

almost equally spaced, on each elytron. Elytra in lateral view not bulging, with a flat feature declining towards apex. Small specimens show an accentuated rounder body. L/EL ratio 1.628, significantly higher than in *M. gyas,* showing a bulker body with shorter elytra: elytra in *M. hyperion* sp. nov. are shorter and broader. This fact gives to *M. hyperion* sp.nov. an obviously "squared" look. **Pygidium**. Convex, covered by a yellowish pubescence. **Abdomen**. Laterally covered with very fine, short, yellowish-brown pilosity, medially on sternites III-IV-V almost glabrous. **Legs.** Fore tibia almost straight, the inner edge rather dilated inwards at apex, 21 mm. length. The anterior edge V-shaped in all specimens, just over the first tarsomere. External sides of tibiae with three teeth, decreasing in length from the basal to the apical tooth; the basal tooth more distant from the subapical than the latter from apical. Basal and subapical teeth large, sharp, tringular, pointing rearwards; apical tooth sharp, pointing forwards. Inner apical spur strongly curved ventrally, as long as the basal tooth. Fore tarsus 24 mm. of length. Mesotibia and metatibia with three very pointed teeth increasing in length from the basal to the apical; first tarsomere in middle and hind tarsi very acute. **Aedeagus.** Intermediate between *M. gyas* and *M. typhon*, more massive than the former, less massive and more rounded than the latter, as show in Tab. 2E-F. **Bionomics.** Very few are the informations on the beetle's behaviour. The beetle flies usually from 9-10 p.m. till 2 hours in the morning and it is attracted by white mercury's lights, the number of ♂ being the more frequent (E. J. Grossi, personal comunication, 2019). **Etymology.** Noun in apposition. Following the exemple of Jablonsky who chose the name of a Titan, Gyas, we also draw to the greek mythology and choose the name of another Titan, son of Uran (the sky) and Gea (the earth): Hyperion.

Variation of males. Overall morphology quite homogeneus. Proportionally, in CL, the measured difference is slight. The shape of cephalic horn shows the most interesting variability, e.g. more elongate VS. more squared; triangular shape VS. subrectangular, even if all the paratypes maintain the same V-shaped apex with regular/short tips.

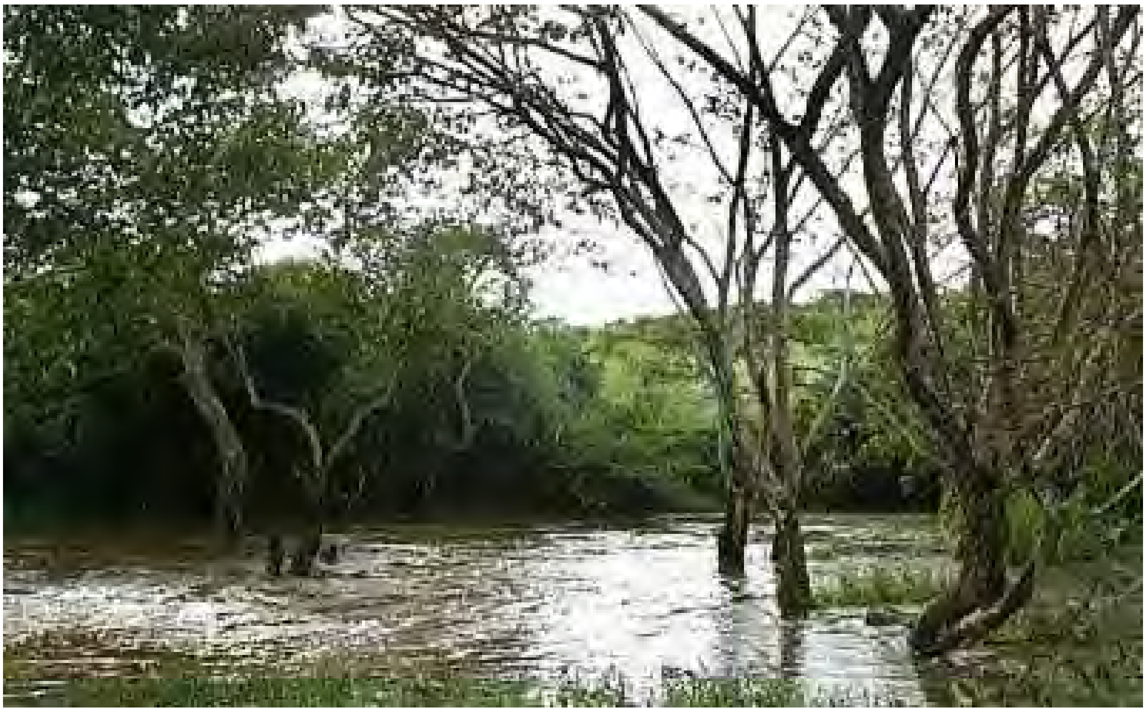

Fig. 23. View of Aguas Vermelhas, Rio Mosquito, Minas Gerais, Brazil. (www.minas-gerais-brasil.blogspot.com)

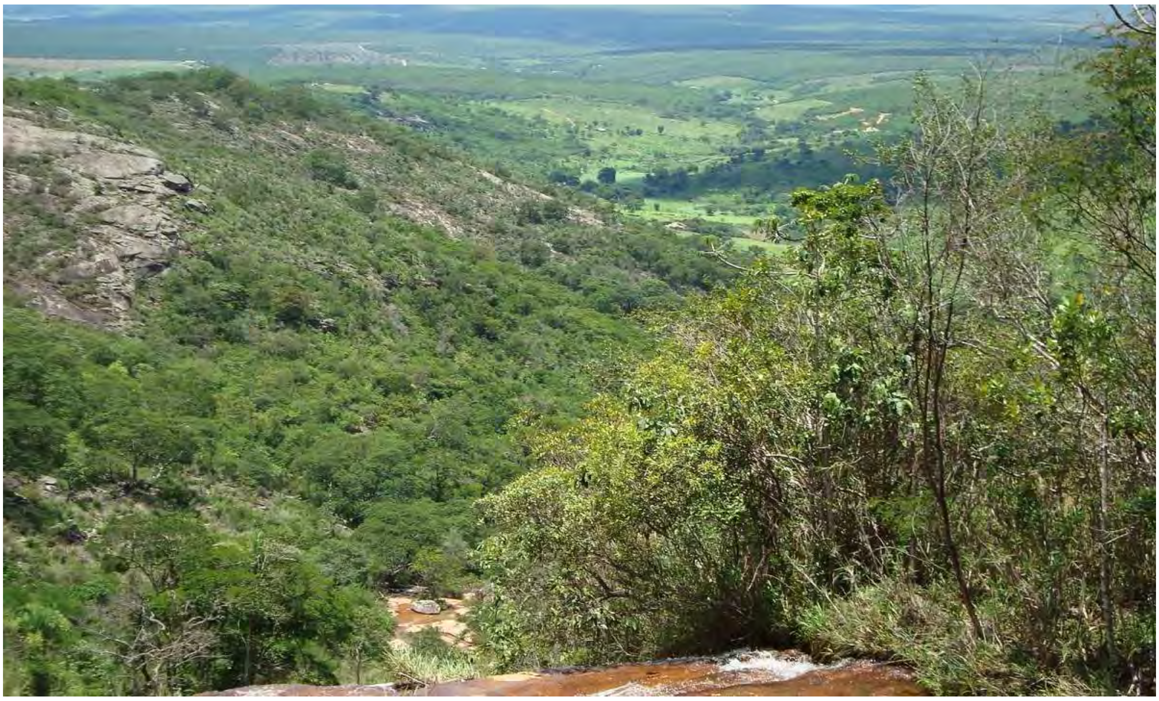

Fig. 24. View of Aguas Vermelhas, Serra do Anastácio, Minas Gerais, Brazil with enclaves of Cerrado and Caatinga biomes. (www.minas-gerais-brasil.blogspot.com)

Description of the female from Brazil, Minas Gerais (Fig. 25).

Dimensions. L, in **Allotype**: mm. 64; PL: mm. 18; PW: mm. 27; EL: mm. 42; EW: mm. 38; HL: mm. 8. **Color.** Uniformly black; elytra with 3/4 of its surface covered by yellowish-brown dense recumbent pilosity. **Head**. The middle of fronto-clypeal suture with a single tubercle. In ventral view, inter-ocular distance length 3.4 mm.; transverse eye diameter width 3.5 mm. **Clypeus.** Finely punctate; anterior lateral angles projecting into a tooth directed forwards and upwards; distance between tips 2.5 mm.; apical edge between the angles concave. **Pronotum**. Dull, coarsely punctate-rugose, strongly convex; posterior medial carina 11 mm. long, more than ½ of total length. Anterior angles projecting, obtuse, with blunt tips. **Scutellum**. Triangular, smooth, shiny, impunctate. **Elytra.** Surface glossy black, rugo punctate at anterior region, near base coarser; punctate surface extending for 12 mm. in length, almost 1/5 of L. Elytral pilosity very uniform, yellowish-brown, with easy detectable longitudinal ridges, three or more for each elytron, almost equidistant. Dorsal longitudinal and lateral edges glossy black, vith very fine punctuation. **Pygidium.** In lateral view, concave, with very fine punctuation. Surface in basal half covered with short, fine, greyish pubescence; in apical half with scattered, erected brown-yellowish setae. **Abdomen.** Sternites very finely punctate, covered by short, yellowish-brown pilosity, except for a small central portion in the middle of sternites III-IV-V. **Legs**. Protibiae shorter than tarsi, shorter than in the males, tarsi shorter too; FL mm. 17, TF mm. 20. External sides with three strong teeth almost equidistant. The basal and the subapical teeth almost equal in length; the apical tooth smaller. Inner side without a strong dilatate apex. Inner spur curved ventrally and shorter than apical tooth. On mesotibiae and metatibiae three lateral sharp teeth, with the subapical and the apical weakely evolving in lateral carinae, with presence of basal embrional spinous processes. **Measurements of female paratypes.** The variability of examined paratypes ranges in mm. as follows. L: 48-71; PL: 14-20; PW: 21-33; EL: 32-48; EW: 27-38; FL: 11-17; TF: 16-20; HL: 6-8.

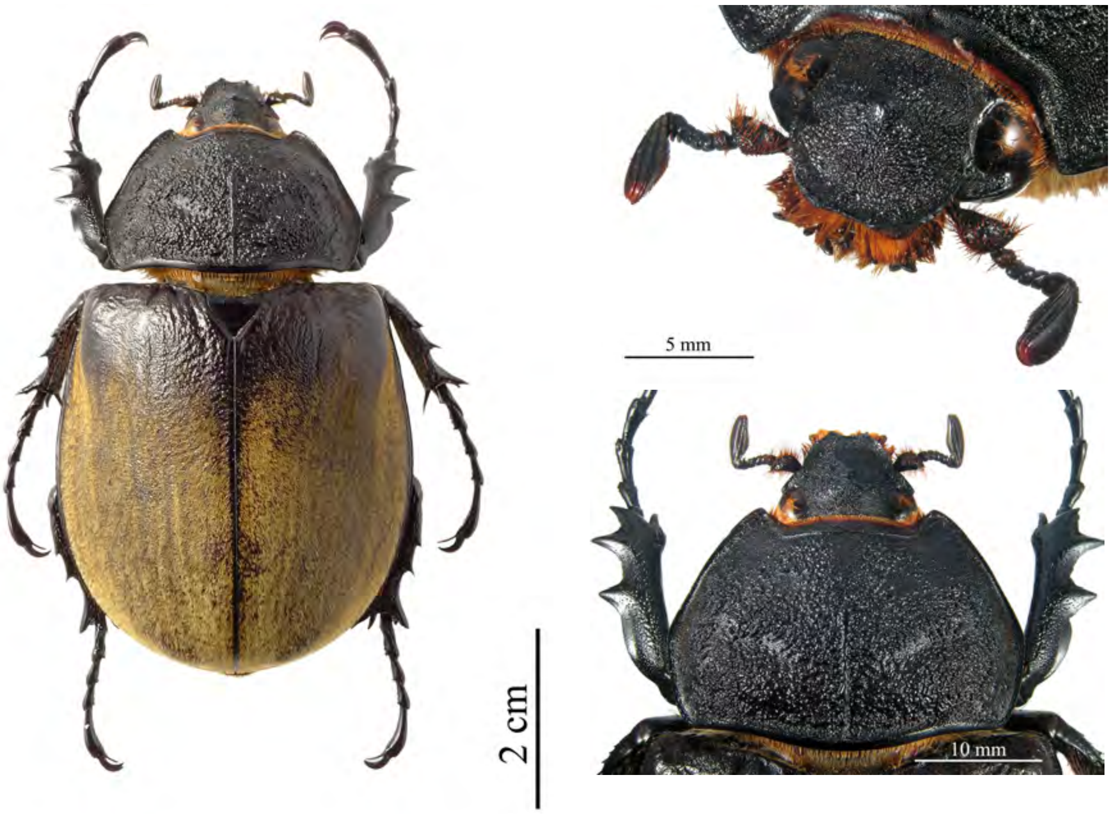

Fig. 25. Dorsal and lateral view with detail of single head tubercle and pronotal carina of *Megasoma hyperion* sp. nov. from Brazil, MG, Aguas Vermelhas. **Allotype ♀**.

Comparative notes

M. hyperion sp. nov. is here compared with *M. gyas*. Brief scientific notes are given also on the other related species of the complex *gyas-typhon*, such as the pubescent Southamerican taxa *M. joergenseni* Bruch, 1910, *M. joergenseni* ssp. *peynai* Nagai, 2003, *M. anubis* (Chevrolat *in* Guerin, 1836) and the "naked-*typhon*" *M. hermes* Prandi, 2016.

***M. gyas Vs. M. hyperion* sp. nov. - confrontation**

M. gyas	***M. hyperion* sp. nov.**
Cephalic horn apex U-shaped with long tips	Cephalic horn V-shaped with regular/short tips
Cephalic horn dorsal triangle tooth minimum or absent	Cephalic horn dorsal triangle tooth present
Habitus elongated	Habitus more squared (elytra shorter and wider)
Aedeagus thin, smaller and elongate (Fig.)	Aedeagus bulkier (Fig.)
Double female head tubercle	Single female head tubercle

Related Southamerican taxa

***Megasoma (Lycophontes) joergenseni joergenseni* (Bruch, 1910).**

The subgenus *Lycophontes,* of the Genus *Megasoma*, is a group comprised of small sized taxa. *Megasoma jorgenseni jorgenseni* is a completely-pubescent taxon, occurring in Central-Northern Argentina and Southeastern Bolivia. The variability of the total body length ranges usually from 30 to 40 mm. (Figs 26A-B, courtesy Mushisha). The original type of Bruch (San

Luis, Mendoza) up to now considered lost, was recently found by the Argentinian Entomologist F. C. Penco in a private collection, and now deposited in the Museum of the University of La Plata.

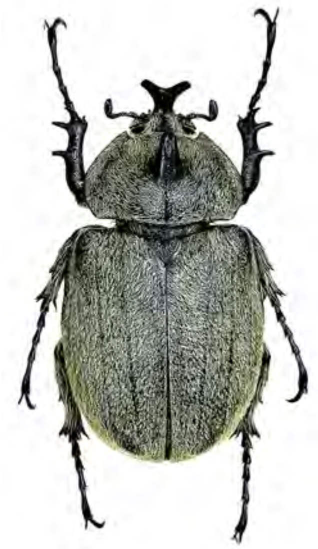
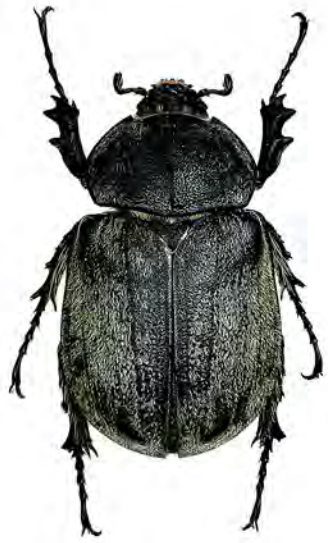

Fig. 26A **Fig. 26B**

Megasoma (Lycophontes) joergenseni penyai **Nagai, 2003.**
The subspecies *penyai* is restricted to an area in Central-Western Paraguay (Holotype from Loma Plata, Chaco). The main distinctive characters differentiating it from the subspecies *joergenseni* are found mainly in the smaller medial thoracic horn and in the denser pubescence, giving a more brownish color. It is also usually smaller than ssp. *joergenseni.* (Figs 27A-B, courtesy Mushisha).

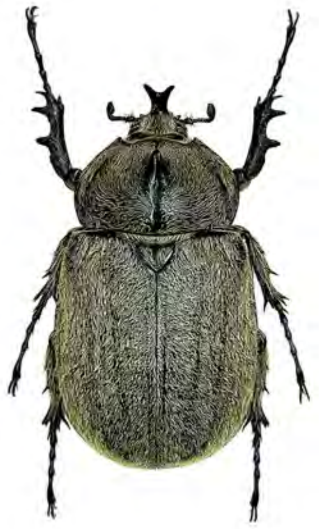
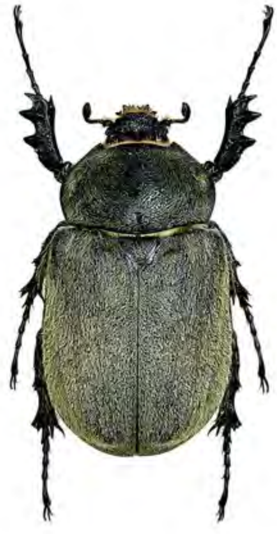

Fig. 27A **Fig. 27B**

Megasoma **(*Megasoma*) *anubis*** **(Chevrolat *in* Guerin, 1836).**
syn.: *Scarabaeus hector,* Gory, 1836
syn.: *Megalosoma theseus,* Laporte, 1840
This is another completely-pubescent South American taxon belonging to the Genus *Megasoma* . It occurs in Northeastern Argentina (Misiones region), Southeastern Paraguay

and in Eastern and Southern Brazil (Espírito Santo, Rio de Janeiro, Paraná, Santa Catarina and Rio Grande do Sul states). It is interesting to note that in the first two Brazilian States, namely in the region of Teresópolis, RJ, it is syntopic with *Megasoma typhon typhon.* The distinctive characters are the cephalic horn and the sickle-shaped thoracic horn both are short with a very wide bifurcated apex. Total body length ranges from 50 to 90 mm. (Figs 28A-B), courtesy Mushisha).

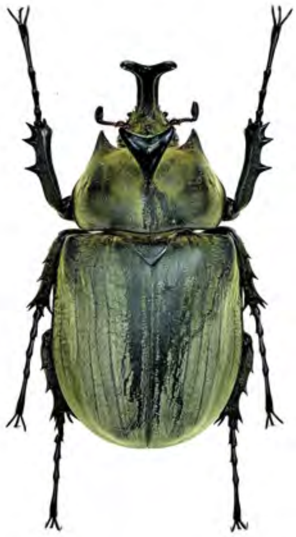

Fig. 28A

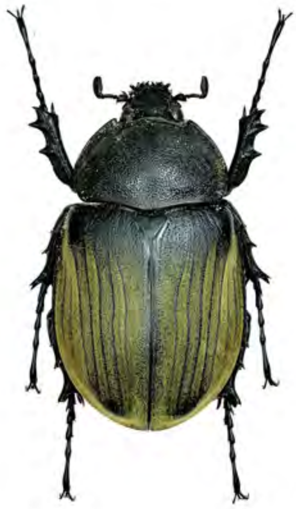

Fig. 28B

***Megasoma* (*Megasoma*) *hermes* Prandi, 2016.**

The distribution range of this species (Northern Brazil, border with Venezuela and Guyanas) represents the northernmost distribution compared to the aforementioned species and subspecies. It is likely that Endroedi's (1977) and Morón's (2005) claims on the alleged presence of *M. gyas* in Suriname and Guyana, refer to this species. The validity of this taxon has been recently confirmed by further findings from Venezuela, at the border with Brazil (see Kobayashi 2019). A large specimen of *M. hermes* is deposited in the Collection of MSNM. It is a completely glabrous *Megasoma* on its dorsal side. Ventrally it bears sometimes a very poor pubescence, mainly on female specimens. The variability of total body length ranges from 68 to 105 mm. (Figs 29A-B, courtesy Mushisha). Only ten specimens have been collected thus far.

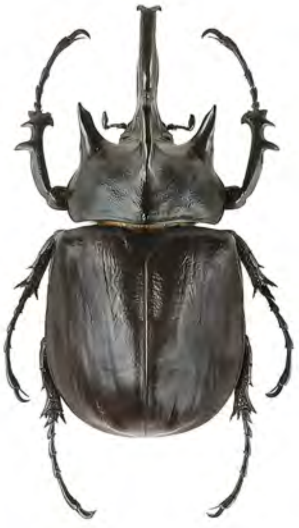

Fig. 29A

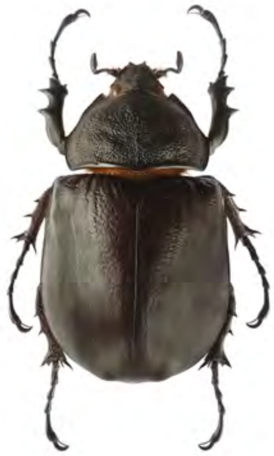

Fig. 29B

Identification keys for major males and females of the *Megasoma* (*Megasoma*) *gyas* species group. Identification of minor males requires the examination of the aedeagus.

Males:

1. Dorsum covered by pubescence, pronotum three-horned ....***gyas*** species group 2

- Different combination of characters...other ***Megasoma*** species

2. Cephalic horn wide and short (< 30 mm.)...3

- Cephalic horn elongate, medium to long (> 30 mm.)..4

3. Cephalic horn with apex U-shaped with long tips.......................................***Megasoma gyas***

- Cephalic horn with apex V-shaped with short tips..........................***Megasoma hyperion* n. sp.**

4. Cephalic horn medium to long, straigth..***Megasoma typhon typhon***

- Cephalic horn long, curved upwards and backwards......................***Megasoma typhon prandii***

Females:

1. Female with a single cephalic tubercle..2

- Female with two cephalic tubercles...***Megasoma gyas***

2. Sides of pronotum rounded...***Megasoma typhon prandii***

- Sides of pronotum subtrapezoidal................................***M. typhon typhon*, *M. hyperion* n. sp.**

Fig. 30. Areas of distribution

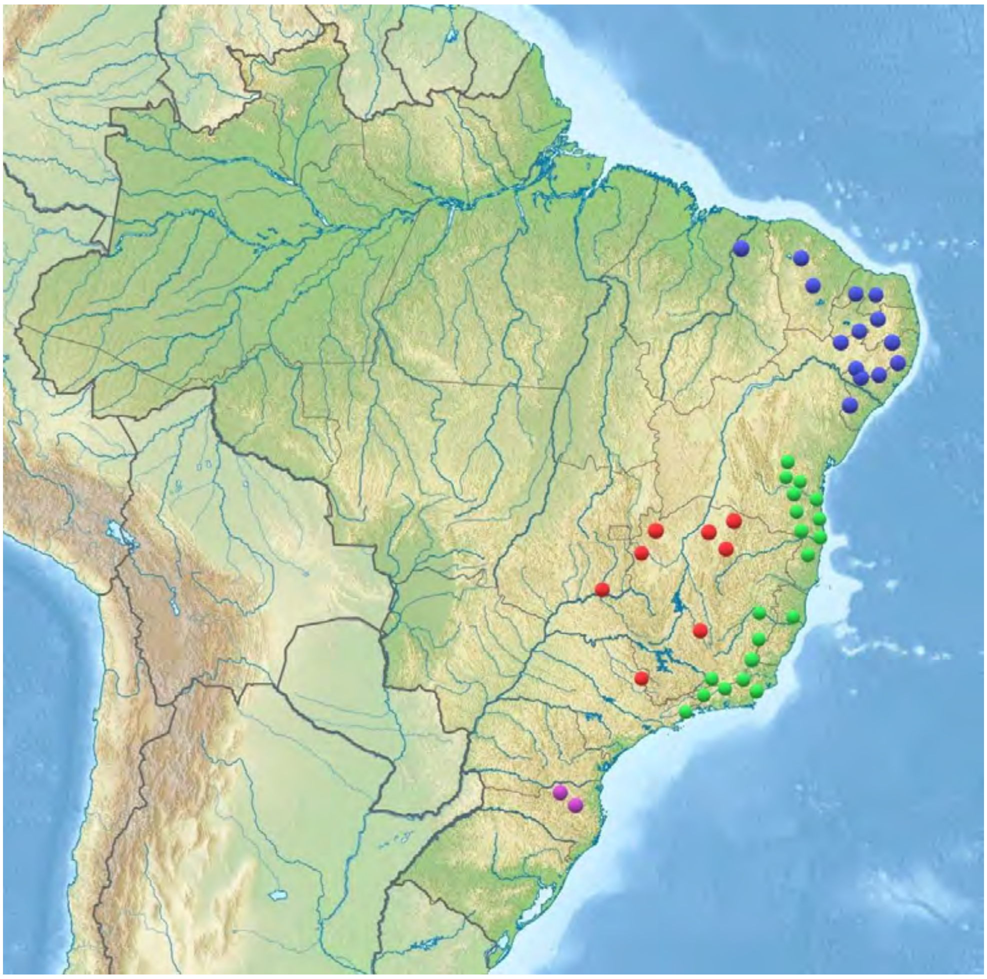

Megasoma gyas *Megasoma typhon typhon* *Megasoma typhon prandii* *Megasoma hyperion*

Table 1: list of Megasoma species (from Prandi 2016, updated)

M. actaeon (Linnaeus, 1758)	Venezuela, Guyanas, Brazil (AM, MA, PA, AP)
M. anubis (Chevrolat *in* Guerin, 1836)	NE Argentina, SE Paraguay, Brazil (ES, RJ, PR, SC, RS)
M. cedrosa Hardy, 1972	North of Baja California, Mexico
M. elephas* ssp. *elephas (Fabricius, 1775)	Veracruz, Mexico to Costa Rica
M. elephas* ssp. *iijimai Nagai, 2003	Colombia, Ecuador and Venezuela to Panama
M. fujitai Nagai, 2003	Brazil, (MT, GO, TO)
M. gyas (Jablonsky *in* Herbst, 1785)	Northeastern Brazil: Caatinga Region
M. hermes Prandi, 2016	Roraima, Brazil, S-Venezuela to French Guiana
***M. hyperion* sp. nov.**	Brazil (MG, SP, South BA)
M. janus* ssp. *janus (Felsche, 1906)	E-Paraguay, S-Brazil (São Paulo)
M. janus* ssp. *argentinum Höhne, 1923	N-Argentina, W-Paraguay, S-Bolivia, SW-Brazil (W-Mato Grosso do Sul, Central and S-Mato Grosso): American Gran Chaco region
M. joergenseni* ssp. *joergenseni Bruch,1910	CN Argentina, SE Bolivia
M. joergenseni* ssp. *penyai Nagai, 2003	CW Paraguay
M. lecontei Hardy, 1972	Baja California sur, Mexico
M. mars (Reiche, 1852)	E-Ecuador, SE-Colombia, NE-Peru, Brazil (RO, AM, PA)
M. nogueirai Morón, 2005	Sinaloa, Mexico
M. occidentale Bolivar, Pieltain, Jimenez-Asua & Martinez, 1963	Nayarit to Oaxaca, Mexico
M. pachecoi Cartwright, 1963	South Sonora, Mexico
M. punctulatum Cartwright, 1952	Arizona, U.S.A
M. ramirezorum Silvestre & Arnaud, 2002	Peru, Colombia, Ecuador to Panama
M. rex Prandi, 2018	Bolivia, Peru, Ecuador, Colombia, Brazil (Rondônia and North Mato Grosso)
M. sleeperi Hardy, 1972	South California, U.S.A
M. svobodaorum Krajcik 2009	Bolivia
M. thersites LeConte, 1861	South of Baja California, Mexico
M. typhon* ssp. *typhon (Olivier, 1789)	Brazil, (BA, ES, RJ, MG, SP)
M. typhon* ssp. *prandii Milani, 2008	Santa Catarina, Brazil
M. vazdemelloi Prandi, 2018	Mato Grosso, Brazil
M. vogti Cartwright, 1963	South Texas, U.S.A. to Nuevo Tamaulipas, Mexico

Table 2 : aedeagi

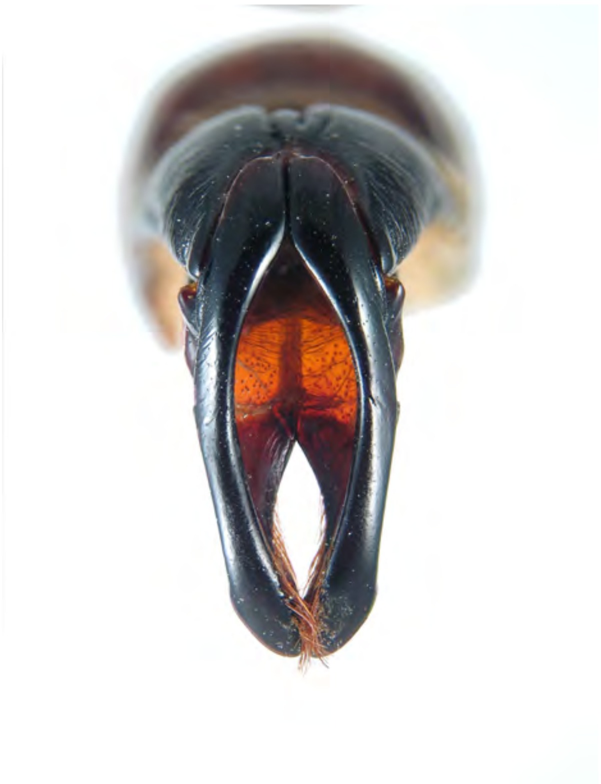

Table 2A

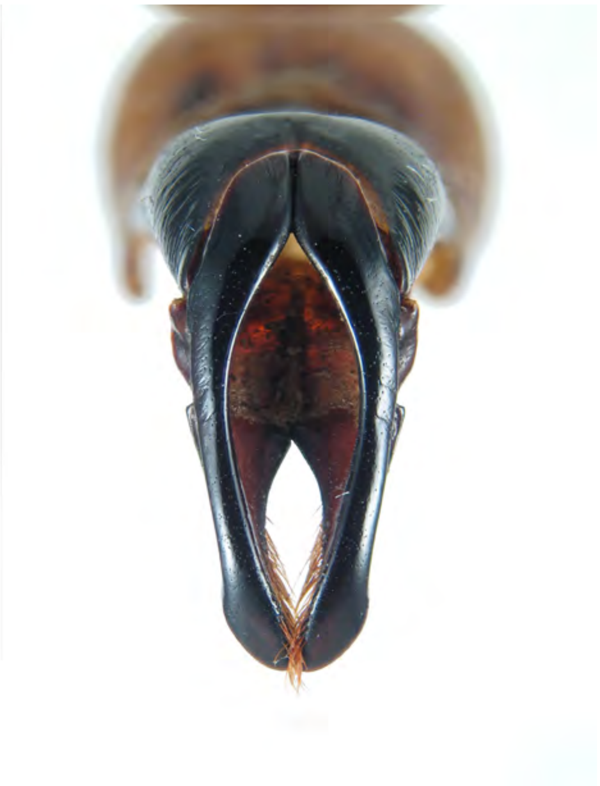

Table 2B

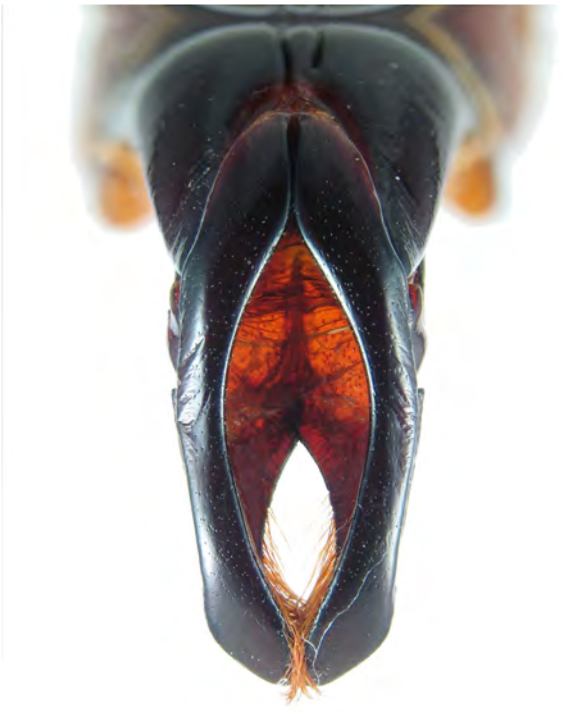

Table 2c

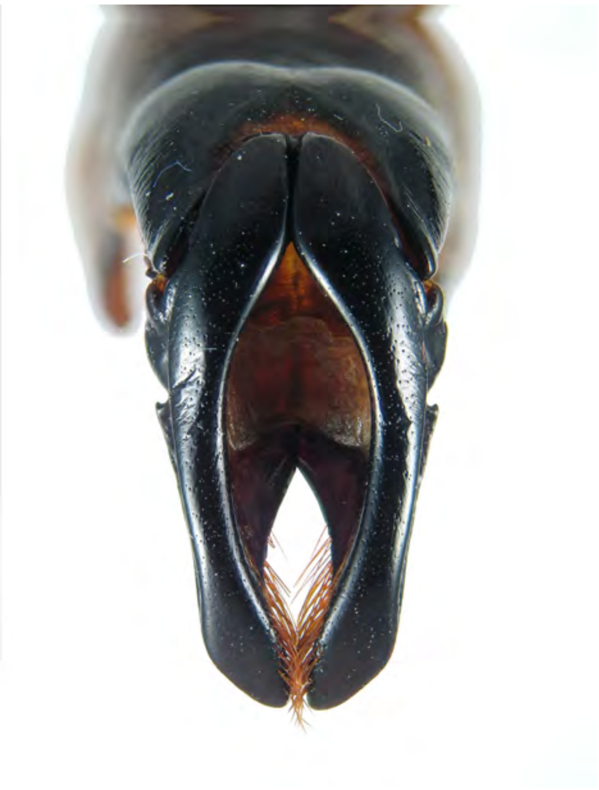

Table 2d

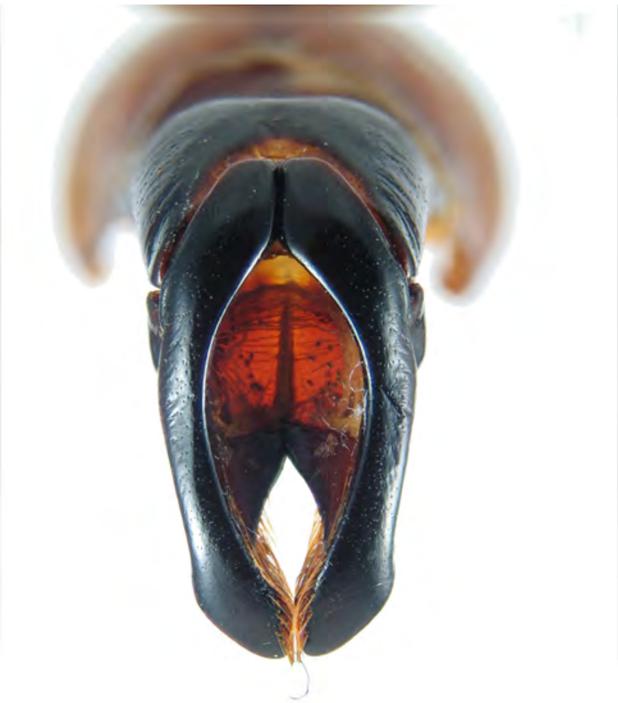

Table 2E

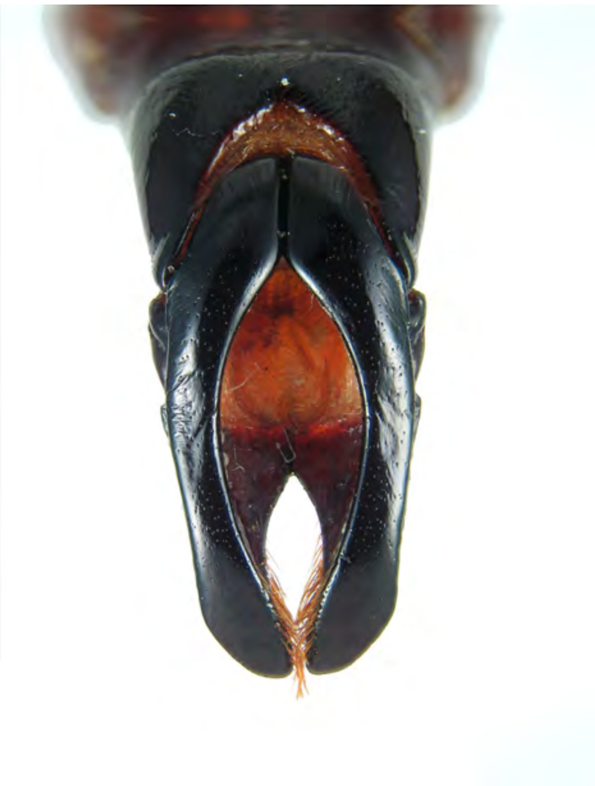

Table 2F

Table 2. A: Frontal view of aedeagus of *M. gyas* from Custódia, Pernambuco, Brazil; **B:** Frontal view of aedeagus *M. gyas* from Sergipe, Brazil; **C:** F. view of aedeagus of *M. typhon typhon* from Jaguaquara, Bahia, Brazil; **D:** F. view of aedeagus of *M. typhon typhon* from Ipatinga, Minas Gerais, Brazil; **E and F:** F. view of aedeagus of *M. hyperion* sp. nov. from Aguas Vermelhas, Minas Gerais, Brazil.

Acknowledgements
We would like to thank Michele Zilioli (MSNM) for all the photographs of habitus and aedeagi included in the work, except for photos Figs 26A-B, 27A-B, 28A-B and 29A-B which are kind courtesy of Mushisha (Japan). Thanks to Alberto Ballerio for the revision of the paper, Fabrizio Rigato (MSNM) for allowing to use the facilities of MSNM, all the Curators included in the list of consulted Museums and Institutions and Kazuho Kobayashi for his help. Thanks to Editors of sites mentioned in the pictures taken from the web. Special thanks also to Everardo J. Grossi for his precious suggestions and for kindly allowing to examine his collection. Fernando Z. Vaz-de-Mello is a CNPq fellow, and parts of this work were funded by CNPq (Conselho Nacional de Desenvolvimento Cientifico e Tecnologico, Brasil, processes number 304925/2010-1, 302997/2013-0, 405697/2013-9, 484035/2013-4 and 202327/2013-2) and FAPEMAT (Fundacao de Amparo à Pesquisa do Estado de Mato Grosso).

References

Anderson C (2019) The Old Indies at the French Court. Early Modern Low Countries 3: 32-59. https://doi.org: 10.18352/emlc.89

Antunes AZ, Rapp de Eston M, Rodrigues dos Santos AM (2007) O Escaravellho *Megasoma gyas* (Herbst, 1775), Espécie Ameaçada de Extinção, No Parque Estadual Carlos Botelho, Sete Barras – SP. Revista do Instituto Florestal São Paulo 19: 129-135.

Beserra Nobre CE, Bezerra Souza T, Vieira Nunes R, Avello Nicola P, Machado Pereira LC (2014) A new distribution record of the threatened *Megasoma gyas rumbucheri* Fisher, 1968 (Coleoptera: Scarabaeidae: Dynastinae) Northward to the Rio São Francisco, Pernambuco, Brazil. The Coleopterist Bulletin 68: 762-764. https://doi/10.1649/0010-065X-68.4.762

Boeseman M, Holthuis LB, Hoogmoed MS, Smeenk C (1990) Seventeenth century drawings of Brazilian animals in Leningrad. Zoologische Verhandelingen 267: 1-189.

Bousquet Y (2016) Litteratura Coleopterologica (1758-1900): a guide to selected books related to the taxonomy of Coleoptera with publication dates and notes. Zookeys 583: 1-776. https://doi.10.3897/zookeys.583.7084

Burmeister H (1847) Handbuch der Entomologie, Vol.V, Coleoptera Lamellicornia, Xylophila et Pectinicornia. G. Reimer, Berlin, 584pp. https://doi.org/10.5962/bhl.title.8135

Corrêa do Lago B (2010) Frans Post e o Brasil Holandés na coleção do Instituto Ricardo Brennand. Catalogo da Exposiçao Permanente. Instituto Ricardo Brennand, Recife, 137pp.

Costa Lima AM da (1953) Insetos do Brasil, 8 Tomo, Coleópteros, 2 Parte. Escola Nacional de Agronomia, Série Didactica, 10. Rio de Janeiro, 372pp.

Coutinho LM (2016) Biomas Brasileiros. Oficina de Textos, São Paulo, 124pp.

Dantas Silva L (2005) 1630-1654, Holandéses em Pernambuco: 1630-1654. 2 Edição, Revista e muito ampliada. Instituto Ricardo Brennand, Recife, 318pp.

Endrödi S (1977) Monographie der Dynastinae. (Coleoptera) 6. Tribus Dynastini II. Acta Zoologica Academiae Scientiarum Hungaricae 23: 37-86.

Endrödi S (1985) The Dynastinae of the World. Dr. W. Junk Publisher, Dordrecht, 800pp.

Felsche C (1906) Synonymische Bemerkungen über einige Scarabaeiden aus der Tribus der Dynastini und Beschreibung einer neuen Art. Deutsche Entomologische Zeitschrift, Berlin. Heft II. 349-352.

Fischer H (1968) Zwei neue *Megasoma*-Arten aus Amerika. Bericht der Naturforschenden Gesellschaft Augsburg 022: 137-142.

Françoso M (2012) “Dressed like an Amazon”: The transatlantic trajectory of a red feather coat. In: Hill K. (Ed.) Museums and Biographies: Stories, Objects, Identities. Boydell and Brewer, London: 187-199.

Fuessly JK (1778) Magazin fur die Liebhaber der Entomologie. Bey dem Herausgeber, und bey Heinrich Steiner und Comp. Zurich und Winterthur, 300pp.

Goeze JAE (1777) Entomologische Beytrage zu des Ritter Linné zwölften ausgabe des Natursystems. Erster theil. Bey Weidmanns Erben und Reich, Leipzig, 736pp. https://doi.org/10.5962/bhl.title.45974

Grossi EJ, Vaz-de-Mello F, Coelho Grossi P (2008) In Machado ABM, Drumond GM, Paglia AP (Eds), Livro Vermelho da Fauna Brasileira Ameaçada de Extinçao. Ministerio do Meio Ambiente, Secretaria de Biodiversidade e Florestas. Departamento de Conservaçao e Biodiversidade. Biodiversidade 19, Brasilia, DF. pp.364-367.

Hagen HA (1857) Nachricht uber das Kaferwerk von Joh. Euseb Voet. Entomologischer Verein zu Stettin Zeitung 18: 405-409.

Harold E von, Gemminger M (1868) Catalogus Coleopterorum hucusque descriptorum synonymicus et systematicus. Monachii, Sumptu E. H. Gummi. E. Deyrolle fils, Paris. 424pp. https://doi.org/10.5962/bhl.title.9089

Harold E von (1871) Berichtigungen und Zusatze zum Catalogus Coleopterorum synonymicus et systematicus. Coleopterologische Hefte. Munchen 8: 117-123.

Huang JP (2107) The Hercules Beetles (Subgenus Dynastes, Genus Dynastes, Dynastidae): a revisionary study based on the integration of molecular, morphological, ecological, and geographic analyses. Miscellaneous Publications, Museum of Zoology, University of Michigan 206: 1-31

Jablonsky CG (1785) Natursystem aller bekannten in-und auslandischen Insekten, als eine Forsetzung der von Buffonschen Naturgeschichte. Nach der System des Ritters Carl von Linné bearbeitet. Der Kafer erster Theil. Mit sechs illuminirten Kupfertafeln. Joachim Pauli Buchhandler, Berlin, 310pp. https://doi.org/10.3931/e-rara-40522

Jablonsky CG, Herbst JFW (1789) Natursystem aller bekannten in-und ausländischen Insekten als eine Forsetzung der von Büffonschen Naturgeschichte: Nach dem System des Ritters Carl von Linné und Fabricius zu bearbeitet von Carl Gustav Jablonsky und fortgesetzt von Johann Friederich Wilhelm Herbst. Der Käfer zweyter Theil. Mit siebzehn illuminierten Kupfertafeln. Joachim Pauli, Berlin, 330pp. https://doi.org/10.5962/bhl.title.156919

Kobayashi K (2019) A synopsis of the genus *Megasoma* (Coleoptera: Dynastinae) in North and South America. Be-Kuwa 70 : 8-47.

Kirby W, Spence W (1822) An Introduction to Entomology, or, Elements of the natural history of insects: with plates. Printed for Longman, Hurst, Rees, Orme and Brown, London, 518pp. https://doi.org/10.5962/bhl.title.65745

Kirby W (1825) A description of such Genera and Species of Insects, alluded to in the "Introduction to Entomology" of Messrs. Kirby and Spence, as appear not to have been before sufficiently noticed or described. The Transactions of the Linnean society Society of London, Volume XIV, Part the Third. Richard Taylor, London, pp. 563-572. https://doi:10.1111/j.1095-8339.1823.tb00103.x

Krell FT (2012) On nomenclature and synonymy of *Trichius rosaceus.* Zootaxa 3278: 61-68. dx.doi.org/10.11646/zootaxa.3278.1.3

Lachaume G (1985) Dynastini,1 : *Dynastes – Megasoma – Golopha*. Les Coléoptères du Monde 5. Sciences Nat., Compiègne, France, 85pp.

Linnaeus C (1758) Systema naturae per regna tria naturae, secundum classes, ordines, genera, species, cum characteribus, differentiis, synonymis, locis, Tomus I. Editiodecima, reformata. Laurentii Salvii. Holmiae, 824pp. https://doi.org/10.5962/bhl.title 542

Luzzi JR, Tagliatti TT, Barbosa BC (2106) Ocorrência de *Megasoma gyas gyas* (Herbst, 1785) (Coleoptera: Scarabaeidae: Dynastinae) em perímetro urbano. Entomotropica 31: 60-63.

Marcgrav de Liebstad G (1648) Historiae naturalium Brasiliae, libri octo. Auspicio et beneficio I. Mavritii (...). Lugnum Bataviorum, apud Franciscum Hackium, Amstelodami, 295pp. https://doi.org/10.5962/bhl.title.565

Milani L (2008) Una nuova sottospecie di Megasoma gyas Jablonsky in Herbst (Coleoptera: Scarabaeidae: Dynastinae) da Santa Catarina, Brasile. Giornale Italiano di Entomologia 12: 119-133

Morón MA (2005) A new species of Megasoma Kirby (Coleoptera: Scarabaedidae: Dynastinae) From Sinaloa, Mexico.Zootaxa, 1037: 29-36. doi.org./10.11646/zootaxa.1037.1.3

Nagai S (2003) Four new subspecies of the genus Megasoma (Coleoptera: Scarabaeidae) from South America. Gekkan-Mushi, (394): 35-39.

Olivier GA (1789) Entomologie ou Histoire Naturelle des Insectes, avec leurs characters génériques et spécifiques, leur description, leur synonyme et leur figure enluminée. Coléoptéres. Vol. I. Baudouin, Paris, 433pp. https://doi.org/10.5962/bhl.title.49479

Olivier GA, Bruguière JG, Lamarck JBPA de M de (1792) Journal d'Histoire Naturelle. T. 1. 4. Imprimerie du Cercle Social, Paris, 480pp.

Ossenbach C (2017) Precursors of the botanical exploration of South America. Wilhem Piso (1611-1678) and George Marcgrave (1610-1644). Lankesteriana 17: 93-103. http://dx.doi.org/10.15517/lankv16i3.27098

Prandi M (2016) Una nuova specie di Megasoma Kirby dal Brasile (Coleoptera, Scarabaeidae, Dynastinae) – Giornale italiano di Entomologia 14: 525-584.

Prandi M (2018a) Synopsis of the species *Megasoma actaeon* (Linnaeus, 1758) (Coleoptera, Scarabaeidae, Dynastinae). Part I. Splitting *actaeon* beetles. Gekkan-Mushi 571: 2-12.

Prandi M (2018b) Synopsis of the species *Megasoma actaeon* (Linnaeus, 1758) (Coleoptera, Scarabaeidae, Dynastinae). Part II. Describing new species. Gekkan-Mushi 574: 49-53.

Prandi M (2019) Synopsis of the species *Megasoma actaeon* (Linnaeus, 1758) (Coleoptera, Scarabaeidae, Dynastinae). Part. III. Reviewing taxa. Gekkan-Mushi 575: 1-5.

Ratcliffe BC (2003) The Dynastinae Scarab Beetles of Costa Rica and Panama (Coleoptera: Scarabaeidae: Dynastinae). Bulletin of the University of Nebraska State Museum 16: 1-506.

Rumbucher K (1991) Studie uber die Variationsbreite von *Megasoma gyas* Herbst 1785 (Coleoptera: Scarabaeidae: Dynastinae). Mit 3 Abbildungen. Entomologische Zeitschrift. Verenigt mit Entomologische Rundschau. 101 Jahrgang 9: 158-161

Santos WE, Alves ACF, Farias RCAP, Creão-Duarte AJ (2013) *Megasoma gyas rumbucheri* Fischer, 1968 (Coleoptera: Scarabaeidae: Dynastinae): first record from a Conservation Unit in Brazil. Entomotropica 28: 233-235.

Sick H (1997) Ornitologia Brasileira 2ed. Editora Nova Fronteira, Rio de Janeiro, 862pp.

Shimizu T (2015) Rhinoceros Beetles of the World. 1 – The new World – Handbook. Series of Insects 6. Mushi-Sha, Tokyo 124pp.

Teixeira DM (1995) Brasil Holandés: Miscellanea Cleyeri, Libri Principis & Theatrum rerum naturalium Brasiliae. V. 5. Index, Rio de Janeiro, 983pp.

Teixeira Leite JR (2014) Arte & arquitetura no Brasil Holandés (1624-1654). Ed. Cepe, Recife, 358pp.

Vasconcellos A, Andreazze R, Almeida AM, Araujo HFP, Oliveira ES, Oliveira U (2010) Seasonality of Insects in the semi-arid Caatinga of Northwestern Brazil – Revista Brasileira de Entomologia 54: 471-476. https://doi.org/10.1590/50035-56262010000300019

Voets JE (1782) Kaferwerk.: Uebersetzt und mit einigen Anmerkungen begleitet. Verlegt von Valentin Bischoff, Nürnberg, 64pp. https://doi.org/10.5962/bhl.title.152190

Voets JE, Panzer GWF (1785) Beschreibungen und Abbildungen hartschaalichter Insekten Coleoptera Linné. Aus dem Original getreu ubersetzt, mit der in selbigem fehlenden Synonymie und bestandigen Commentar versehen von Dr. Georg Wolfgang Franz Panzer. Valentin Bischoff, Nürnberg, 144pp. XLVIII Tab. 134pp. https://doi./10.5962/bhl.title.16107

Voets JE, Bakhuysen G (1806) Catalogus systematicus Coleopterorum. Catalogue systématique des Coléoptères. Sistematische naamlijst van dat geslacht van insecten dat men torren noemt. Bakhuysen, Haag, 111pp. http://doi.org/10.5962/bhl.title16105

Vorst O (2008) On the publication date of Herbst's "Natursystem aller bekannten in-und auslandischen Insekten", Der Kafer IV Theil. The Coleopterist Bulletin 62: 185-188.

Weber F (1801) Observaciones entomologicae, continents novorum quae condidit gererum characters, et nuper detectarum specierum descriptions. Impensis Bibliopolii Academici Novi, Kiliae, 116pp. https://doi.org/10.5962/bhl.title.8639

www.france-pittoresque.com. 1er octobre 1814: mort de l'entomologiste et voyageur Guillaume-Antoine Olivier – Histoire de France et Patrimoine.
